# Iron accumulation induces oxidative stress, while depressing inflammatory polarization in human iPSC-derived microglia

**DOI:** 10.1016/j.stemcr.2022.04.006

**Published:** 2022-05-05

**Authors:** Boyd Kenkhuis, Michelle van Eekeren, David A. Parfitt, Yavuz Ariyurek, Poulomi Banerjee, Josef Priller, Louise van der Weerd, Willeke M.C. van Roon-Mom

**Affiliations:** 1 Department of Human Genetics, Leiden University Medical Center, Postzone S4-0P, P.O. Box 9600, 2300RC Leiden, the Netherlands; 2 Centre for Clinical Brain Sciences, University of Edinburgh, Edinburgh, UK; 3 UK Dementia Research Institute at University of Edinburgh, Edinburgh, UK; 4 Leiden Genome Technology Center, Leiden University Medical Center, Leiden, the Netherlands; 5 Department of Psychiatry and Psychotherapy, School of Medicine, Technical University of Munich, Munich, Germany; 6 Neuropsychiatry and Laboratory of Molecular Psychiatry, Charité, Universitätsmedizin Berlin, BIH and DZNE, Berlin, Germany; 7 Department of Radiology, Leiden University Medical Center, Leiden, the Netherlands

**Keywords:** microglia, iron, induced pluripotent stem cells, oxidative stress, neurodegenerative diseases, neuroinflammation

## Abstract

Iron accumulation in microglia has been observed in Alzheimer’s disease and other neurodegenerative disorders and is thought to contribute to disease progression through various mechanisms, including neuroinflammation. To study this interaction, we treated human induced pluripotent stem cell-derived microglia (iPSC-MG) with iron, in combination with inflammatory stimuli such as interferon gamma (IFN-γ) and amyloid β. Both IFN-γ and iron treatment increased labile iron levels, but only iron treatment led to a consistent increase of ferritin levels, reflecting long-term iron storage. Therefore, in iPSC-MG, ferritin appeared to be regulated by iron revels rather than inflammation. Further investigation showed that while IFN-γ induced pro-inflammatory activation, iron treatment dampened both classic pro- and anti-inflammatory activation on a transcriptomic level. Notably, iron-loaded microglia showed strong upregulation of cellular stress response pathways, the *NRF2* pathway, and other oxidative stress pathways. Functionally, iPSC-MG exhibited altered phagocytosis and impaired mitochondrial metabolism following iron treatment. Collectively, these data suggest that in MG, in contrast to current hypotheses, iron treatment does not result in pro-inflammatory activation, but rather dampens it and induces oxidative stress.

## Highlights

• In iPSC-microglia, iron rather than inflammatory activation induces ferritin expression
• Excessive iron dampens both classic pro- and anti-inflammatory activation
• Iron uptake leads to upregulation of NRF2 and other oxidative stress pathways
• Phagocytosis and mitochondrial metabolism are altered following iron uptake

## Graphical abstract

**Figure:**
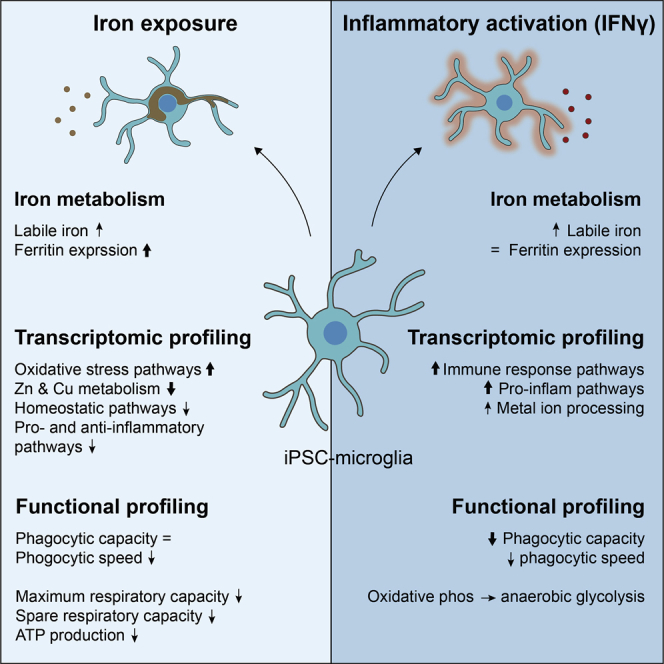

## Teaser

In this article, Kenkhuis and colleagues exposed iPSC-derived microglia to iron and inflammatory stimuli and showed that exclusively iron induced increased ferritin expression. Moreover, excessive iron uptake resulted in dampened pro- and anti-inflammatory activation, but upregulation of oxidative stress pathways. Finally, phagocytosis and mitochondrial metabolism were altered following iron uptake in iPSC-derived microglia.

## Introduction

Iron accumulation is a feature of many different neurodegenerative disorders (NDs) including Alzheimer’s disease (AD), Parkinson’s disease (PD), Huntington’s disease (HD), and multiple sclerosis (MS) (Bulk et al., 2020; Damulina et al., 2020; Popescu et al., 2017; Wang et al., 2016; Ward et al., 2014). Although iron is an essential element for processes such as myelination, neurotransmitter synthesis, and oxidative metabolism (Rouault, 2013), excessive iron is hypothesized to partake in Fenton’s reaction, resulting in an accumulation of toxic reactive oxygen species (ROS) (Smith et al., 1997). Changes in both iron levels and distribution are specifically identified in disease-affected areas of the brain, and excess iron is found to accumulate both extra- and intracellularly. One cell type that has been shown to accumulate iron across different neurological conditions is microglia (Bagnato et al., 2011; Kenkhuis et al., 2021).

Microglia are the resident innate immune cells of the brain and are known to be essential for both brain development and brain plasticity (Paolicelli et al., 2011). As macrophages of the brain, they are also the first responders to changes in brain homeostasis (Prinz et al., 2019). In recent years, microglia have increasingly been implicated in NDs (Salter and Stevens, 2017), primarily in AD. Genome-wide association studies (GWAS) identified the majority of AD risk loci to be primarily or even exclusively expressed by microglia (Efthymiou and Goate, 2017). With regard to iron, microglia are considered the primary cells to sequester excessive amounts of iron in response to an acute insult such as observed in MS lesions (Bagnato et al., 2011). In addition, microglial iron accumulation was also identified in dystrophic microglia surrounding the pathological amyloid β (Aβ) plaques in AD, which was also accompanied by increased expression of the iron-storage protein ferritin (Kenkhuis et al., 2021). Ferritin-positive microglia have been observed repeatedly not only in AD but also in HD and PD (Jellinger et al., 1990; Lopes et al., 2008; Simmons et al., 2007). Moreover, signatures of altered iron metabolism were identified in disease-associated microglia (DAM) in APP/PS1 mice (Keren-Shaul et al., 2017), in human post-mortem AD tissue (Mathys et al., 2019), and in MG from normal-appearing gray matter in MS (van der Poel et al., 2019).

Although there is ample evidence that disrupted microglial iron homeostasis could contribute to disease, there are still several remaining questions. First, as both iron accumulation and neuroinflammation are observed concurrently in disease, it is still unclear whether the iron accumulation and ferritin expression observed in microglia in different NDs are a consequence of increased iron concentrations and/or iron depositions, a result of inflammatory activation, or a combination of both. Evidence from murine microglia and macrophages does suggest that pro-inflammatory activation promotes labile iron uptake both *in vitro* and *in vivo*, but it does not induce protein expression of light-chain ferritin, which is responsible for long-term iron storage (Holland et al., 2018; McCarthy et al., 2018). Second, it is unknown how iron affects microglial activation and function. Iron was found to be capable of potentiating pro-inflammatory interleukin (IL)-1β secretion induced by Aβ via nuclear factor (NF)-κB pathway activation in murine microglia (Nnah et al., 2020), and non-ferritin stored labile iron induced IL-1β production in peripheral mononuclear cells (Nakamura et al., 2016). Conversely, others found that iron decreases polarization toward M1 macrophages and inhibits the pro-inflammatory response (Agoro et al., 2018; Gan et al., 2017). Nonetheless, all of these data were obtained for murine microglia or even murine macrophages, and further investigation using human-derived microglia-like cells is warranted, considering there are significant differences between human and murine microglia and macrophages (Smith and Dragunow, 2014).

In this study, we used human induced pluripotent stem cell-derived microglia (iPSC-MG) to examine the effect of increased iron levels on Mmicroglia under normal and inflammatory conditions. Although iPSC-MG do not fully recapitulate the complete transcriptomic, morphological, and functional profile of microglia in the human brain, they are considered suitable to study the mechanisms involved in the processing of external stimuli. We treated iPSC-MG with the iron compound ferric citrate (FC) with or without the pro-inflammatory type II class cytokine interferon gamma (IFN-γ) or Aβ. Increasing concentrations of iron led to consistent increases in intracellular labile iron and ferritin expression. Treatment with IFN-γ only increased intracellular labile iron but not ferritin expression, while Aβ treatment had no effect on either ferritin expression or intracellular labile iron concentrations. On a transcriptomic level, iron-treated microglia showed disparate activation from the IFN-γ-induced pro-inflammatory pattern; iron even inhibited the activation of the NF-κB pathway, instead inducing cellular detoxification and oxidative stress. In addition, functional changes in both phagocytosis and mitochondrial metabolism were observed in response to iron treatment. Our study shows that high iron levels induce iron sequestration and ferritin storage in human iPSC-MG, which causes oxidative stress without polarization toward classic pro- or anti-inflammatory activation.

## Results

### Generation and characterization of hiPSC-derived MG-like cells

**Figure 1:**
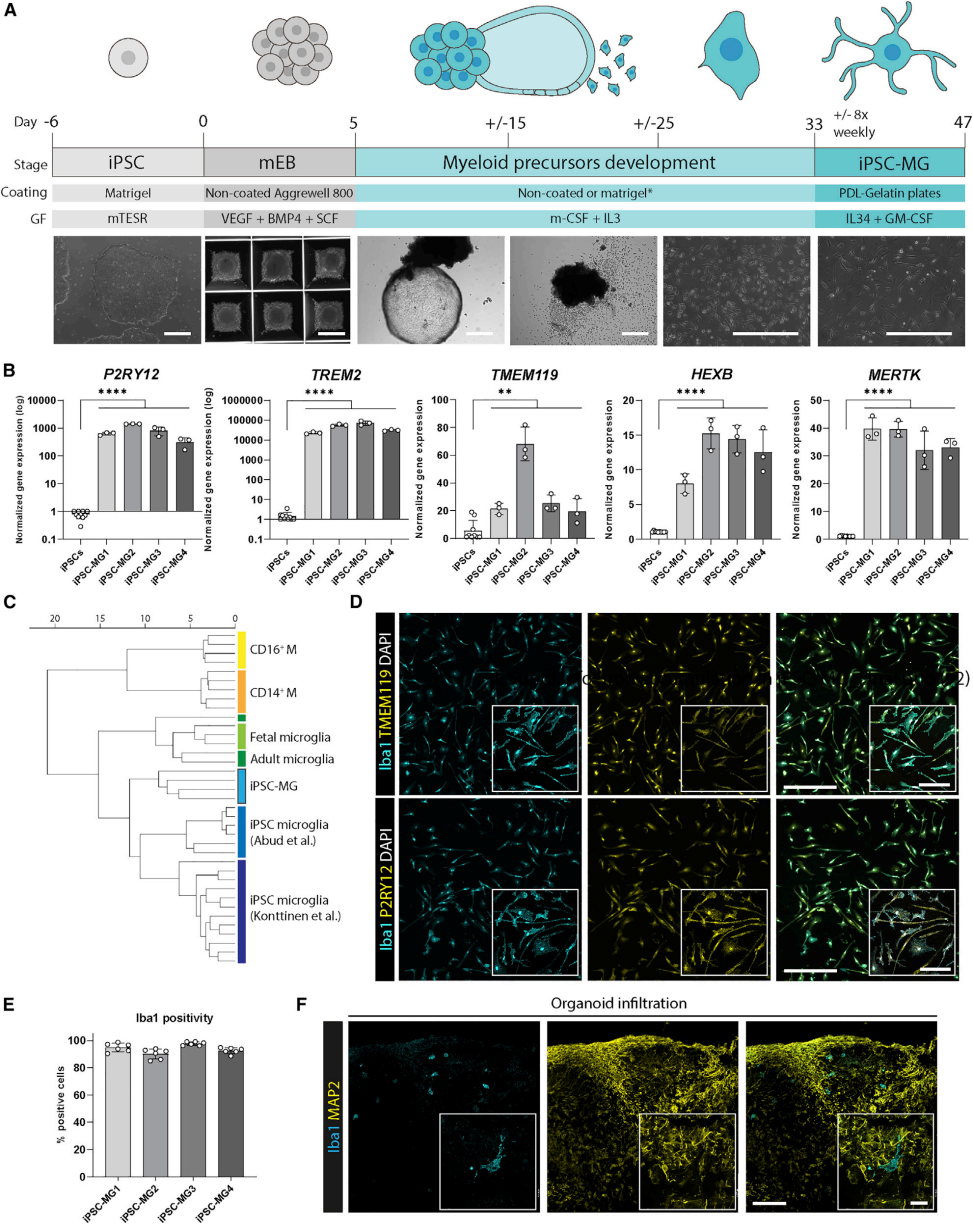
Generation and characterization of human iPSC-derived microglia (iPSC-MG) (A) Schematic representation of the differentiation protocol (supplemental experimental procedures). Scale bar, 500 μm. ^∗^Coating dependent on iPSC-line. (B) iPSC-MG showed significant upregulation of microglia signature genes P2RY12, *TREM2*, *TMEM119*, *HEXB*, and *MERTK* via qPCR (n = 12, 3 independent experiments for 4 iPSC lines; Student’s independent t test). (C) Hierarchical clustering of RNA-seq data shows iPSC-MG to cluster with other published iPSC-derived microglia and distinct from CD14^+^ and CD16^+^ monocytes (M). (D) Using immunofluorescent staining, the majority of cells were positive for Iba1, P2RY12, and TMEM119. Scale bar, 400 μm; inset scale bar, 100 μm. (E) Quantification of (D). (F) Functionally, iPSC-MG could infiltrate and integrate in human brain organoids (representative images, n = 3, 1–2 independent experiments for 2 iPSC lines). Scale bar, 100 μm; inset scale bar, 20 μm. All of the bar graphs indicate means ± SDs. ^∗^p < 0.05, ^∗∗^p < 0.01, ^∗∗∗^p < 0.001, ^∗∗∗∗^p < 0.0001.

We adapted a protocol by Haenseler et al. (2017) to differentiate human iPSCs into iPSC-MG. The generation of embryoid bodies (EBs) from iPSCs and successful patterning toward mesodermal lineage is shown in Figure 1A. Subsequently, mesodermal EBs (mEBs) were plated in media containing macrophage colony-stimulating factor (m-CSF) and IL-3 to induce the development of erythroid myeloid precursors. In the final step, myeloid precursors were replated and matured into iPSC-MG with the CSF1R ligand IL-34 and granulocyte macrophage (GM)-CSF. Morphological changes were assessed for each stage, and the finally generated iPSC-MG were assessed for expression of core MG markers on both gene and protein levels (Figures 1B–1E). Because of the importance of the *APOE* genotype for microglial function (Lin et al., 2018), iPSC-lines were genotyped for *APOE*, and two lines with the *APOE3/3* genotype and two with the *APOE3/4* genotype were selected (Figure S1A; Table S1). All of the results were compared for the *APOE* allele, but for none of the assays described in the following sections differences were observed depending on the *APOE* allele. Using quantitative real-time PCR (qPCR), iPSC-MG showed a significant upregulation of between 1,000- and 10,000-fold for the core MG signature genes P2RY12 and *TREM2*, and a 10- to 60-fold increase in *TMEM119, HEXB*, *and MERTK* mRNA expression (Butovsky et al., 2014) (Figure 1B). Furthermore, comparison of RNA sequencing (RNA-seq) gene expression profiles using microglia and macrophage genes with the published datasets GSE89189 (Abud et al., 2017) and GSE135707 (Konttinen et al., 2019) revealed that our iPSC-MG hierarchically cluster together with other generated iPSC-MG, but are distinct from blood-derived CD14^+^ and CD16^+^ monocytes (Figure 1C). Using immunofluorescence, iPSC-MG showed clear expression of TMEM119, P2Y12, and Iba1 (Figure 1D). Quantification showed that 85%–99% of cells were positive for Iba1 in each of the 4 iPSC lines (Figure 1E). Finally, we tested the engraftment capacity of our generated iPSC-MG into human brain organoids, as has been demonstrated previously (Abud et al., 2017; Konttinen et al., 2019). We found that iPSC-MG infiltrated the organoids within 24 h after addition to the media, and remained viable close to microtubule-associated protein 2 (MAP2)^+^ neurons (Figure 1F).

### Iron loading in iPSC-MG occurs in response to increased iron concentrations but not inflammatory stimuli

**Figure 2:**
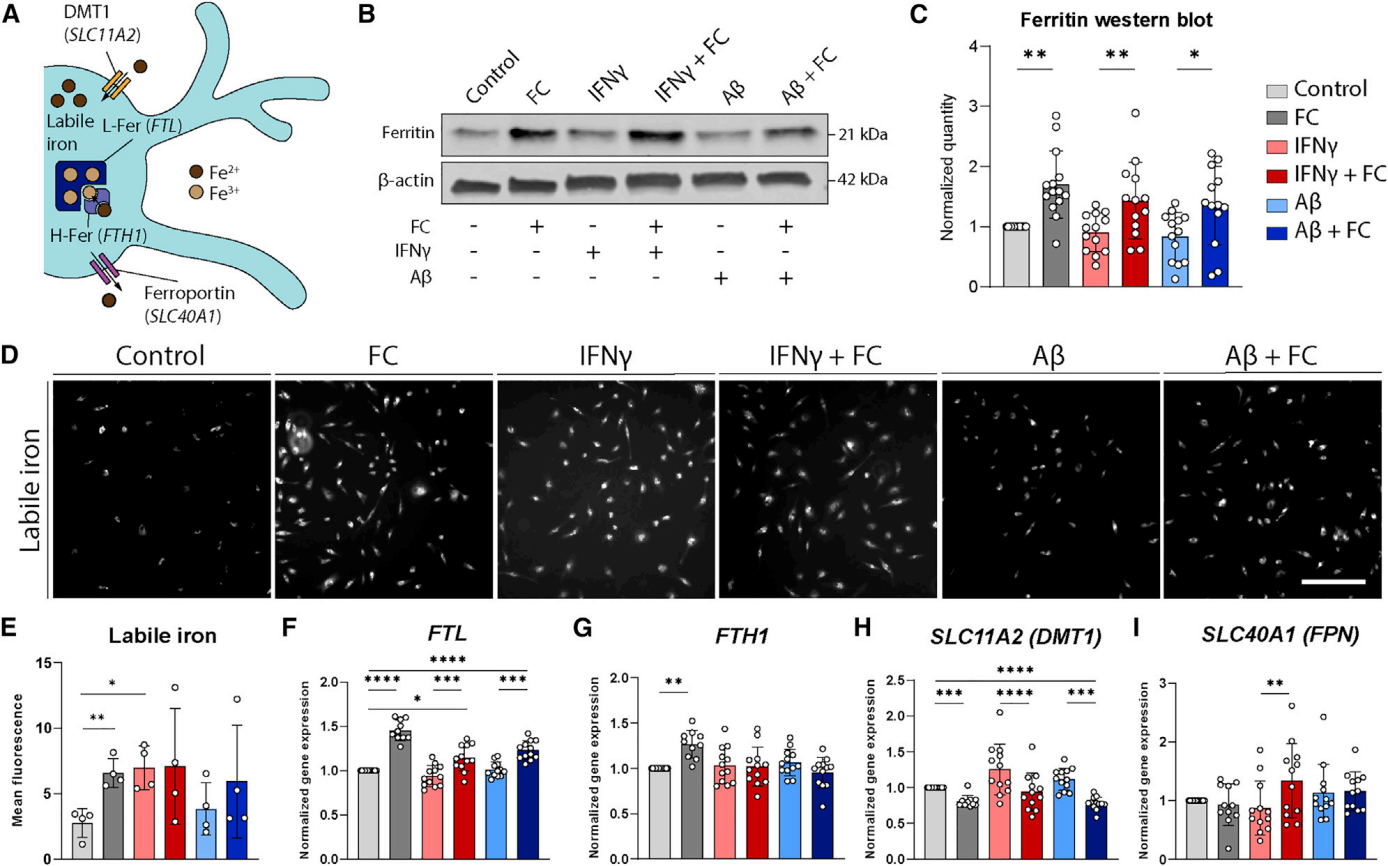
Increased iron concentrations, but not inflammatory activation, lead to microglial iron loading (A) Schematic representation of iron metabolism in MG. Ferrous iron (Fe^2+^) can be taken up by the cell via DMT1 (encoded by *SLC11A2*) and enter the labile iron pool. From here, ferrous iron can be oxidized into ferric iron (Fe^3+^) via ferritin heavy chains (H-Fer, encoded by *FTH1*) and stored in ferritin light chains (L-Fer, encoded by *FTL*). Iron can be transported out of the MG via ferroportin (FPN, encoded by *SLC40A1*). (B) Treatment of human iPSC-MG with 6 different conditions: control, ferric citrate (FC), interferon gamma (IFN-γ), IFN-γ + FC, amyloid β (Aβ), and Aβ + FC. Only FC treatment induces the increased expression of ferritin on western blot. (C) Quantification of (B). (D) Representative images of live cell imaging of labile iron showing increased cytosolic iron after exposure to FC, IFN-γ, or IFN-γ + FC. (E) Quantification of fluorescent intensity in (D) (n = 4, 2 independent experiments for 2 iPSC lines). (F–I) qPCR analysis of iron-metabolism genes shows upregulation of genes *FTL* and *FTH1,* responsible for iron storage; downregulation of *SLC11A2*, coding the iron-importer DMT1; and no difference in *SLC40A1*, coding the iron-exporter FPN, following FC exposure. Neither IFN-γ nor Aβ induces significant changes in iron-metabolism genes. (B) and (F–I) n = 12–14 per condition, 2–4 independent experiments for 4 iPSC lines. Statistical analysis was performed using a mixed-effects model with Geisser-Greenhouse correction and Sidak post hoc correction. Scale bar, 200 μm. All of the bar graphs indicate means ± SDs. ^∗^p < 0.05, ^∗∗^p < 0.01, ^∗∗∗^p < 0.001, ^∗∗∗∗^p < 0.0001.

Following the characterization of our iPSC-MG model, we wanted to assess whether iron accumulation and increased ferritin expression are a consequence of exposure to an increased concentration of iron, a result of inflammatory activation, or a combination of both. In murine microglia, ferritin expression was found to increase following inflammatory stimulation with IFN-γ and/or Aβ (McIntosh et al., 2019). Therefore, we treated our iPSC-MG with an increasing concentration of iron, the inflammatory stimulus IFN-γ, Aβ (1–42), or a combination of iron with IFN-γ or Aβ. Both ferric ammonium citrate (FAC) and FC induced considerable ferritin upregulation (Figure S1B), but we chose FC for further experiments, considering that citrate is also a physiological chelator of ferrous iron (Fe^2+^) in the human brain (Ward et al., 2014). There are multiple genes and proteins involved, a schematic representation of which can be found in Figure 2A. We assessed levels of the iron storage protein ferritin and found a significant increase solely following stimulation with FC and independent of inflammatory activation with IFN-γ or Aβ (Figures 2B and 2C). In addition, using an iron-specific fluorescent tag that assesses non-ferritin bound labile iron, we found both FC and IFN-γ treatment to increase the uptake of iron into the cytosol of iPSC-MG (Figures 2D and 2E). Finally, we assessed the response of the different iron-metabolism genes, responsible for the maintenance of homeostatic iron concentrations. *FTL* mRNA expression was in line with the already observed increase in ferritin protein expression following FC treatment (Figure 2F). We were surprised to find that *FTH1* mRNA expression was only upregulated after FC treatment, but not in combination with IFN-γ or Aβ (Figure 2G). Gene expression of *SLC11A2* (import) was downregulated following FC treatment, likely to prevent further uptake of iron, but slightly upregulated after IFN-γ exposure (Figure 2H). Expression of the transferrin receptor, the alternative importer to *SLC11A2*, could not be detected in our iPSC-MG. Finally, the gene expression of *SLC40A1* (export) was increased only after a combined treatment with FC and IFN-γ, although expression levels varied greatly (Figure 2I). These results show that both FC and the inflammatory stimulus IFN-γ induce an influx of iron into the cytosol of iPSC-MG. However, only FC induces ferritin expression and long-term iron storage.

### Iron loading induces iPSC-MG activation

**Figure 3:**
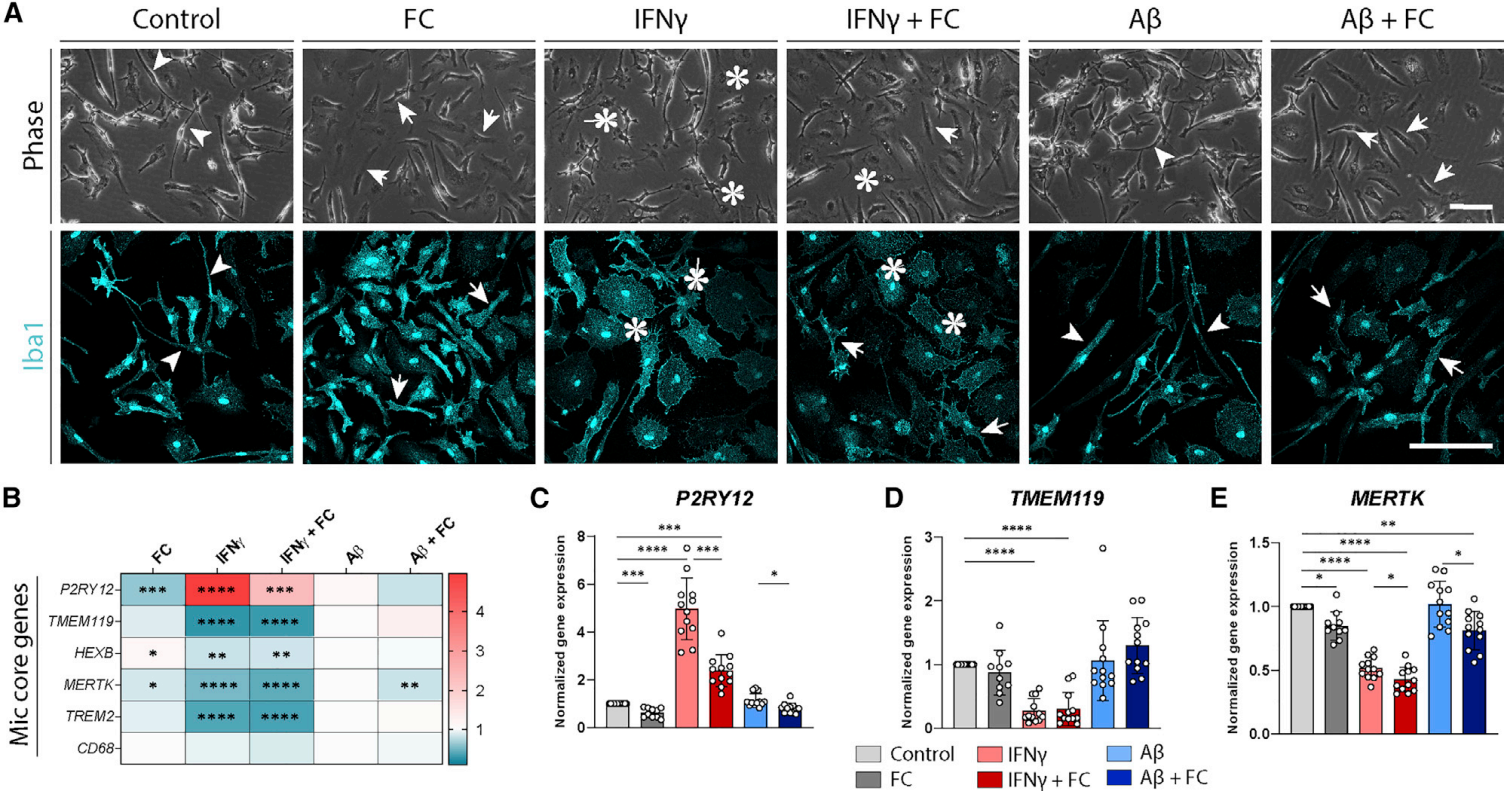
Morphological and transcriptomic activation following iron loading (A) Representative phase and Iba1 immunohistochemical images of human iPSC-derived microglia, showing activation following FC and IFN-γ treatment (total phase images assessed n = 32 per condition, 8 independent differentiations from 4 lines). (B–E) qPCR evaluation of microglia core genes. After IFN-γ treatment, P2RY12 showed strong upregulation, while *TMEM119* and *MERTK* showed strong downregulation. FC induced downregulation of P2RY12 and *MERTK*, albeit less than IFN-γ (heatmap, fold change normalized to control). n = 12 per condition, 3 independent experiments for 4 iPSC lines. Statistical analysis was performed using a mixed-effects model with Geisser-Greenhouse correction for matched data and Sidak post hoc correction. Scale bars, 100 μm. All of the bar graphs indicate means ± SDs. ^∗^p < 0.05, ^∗∗^p < 0.01, ^∗∗∗^p < 0.001, ^∗∗∗∗^p < 0.0001.

Following the characterization of the iron-loading properties of iPSC-MG, we assessed the activation patterns of these cells. We assessed the morphological appearance of iPSC-MG by phase images and after immunohistochemical staining with the actin-cross-linking protein Iba1. IPSC-MG under control conditions showed small cell bodies with long, irregular cell processes (Figure 3A, arrowheads). After FC treatment, the long cell processes were partially retracted and the soma size increased (arrows), although not as much as after IFN-γ stimulation (asterisks). In addition to the increased soma size, IFN-γ stimulation induced a star-like morphology of cells with many shorter processes (asterisks and arrows). Exposure to Aβ induced few morphological alterations compared to control treatment (arrowheads). Next, we assessed the expression of core signature microglia genes, found to be downregulated following microglial activation in AD (Krasemann et al., 2017). Following IFN-γ exposure, all of the genes were found to be downregulated, except for *P2RY12,* which was upregulated both on qPCR (Figure 3B) and immunofluorescence (Figure S1F). Compared to IFN-γ, the gene expression signature was not as strong after FC treatment. FC induced no effect on *TMEM119*, but induced a significant downregulation of the homeostatic gene P2RY12 in all FC^+^ conditions (Figures 3C–3E). In line with the morphological evaluation, Aβ did not induce any significant transcriptomic changes in iPSC-MG (Figure 3B). All in all, iron loading induced the activation of iPSC-MG on both morphological and transcriptomic levels, although disparate from activation following IFN-γ treatment.

### Iron loading depresses both pro- and anti-inflammatory activation patterns in iPSC-MG

**Figure 4:**
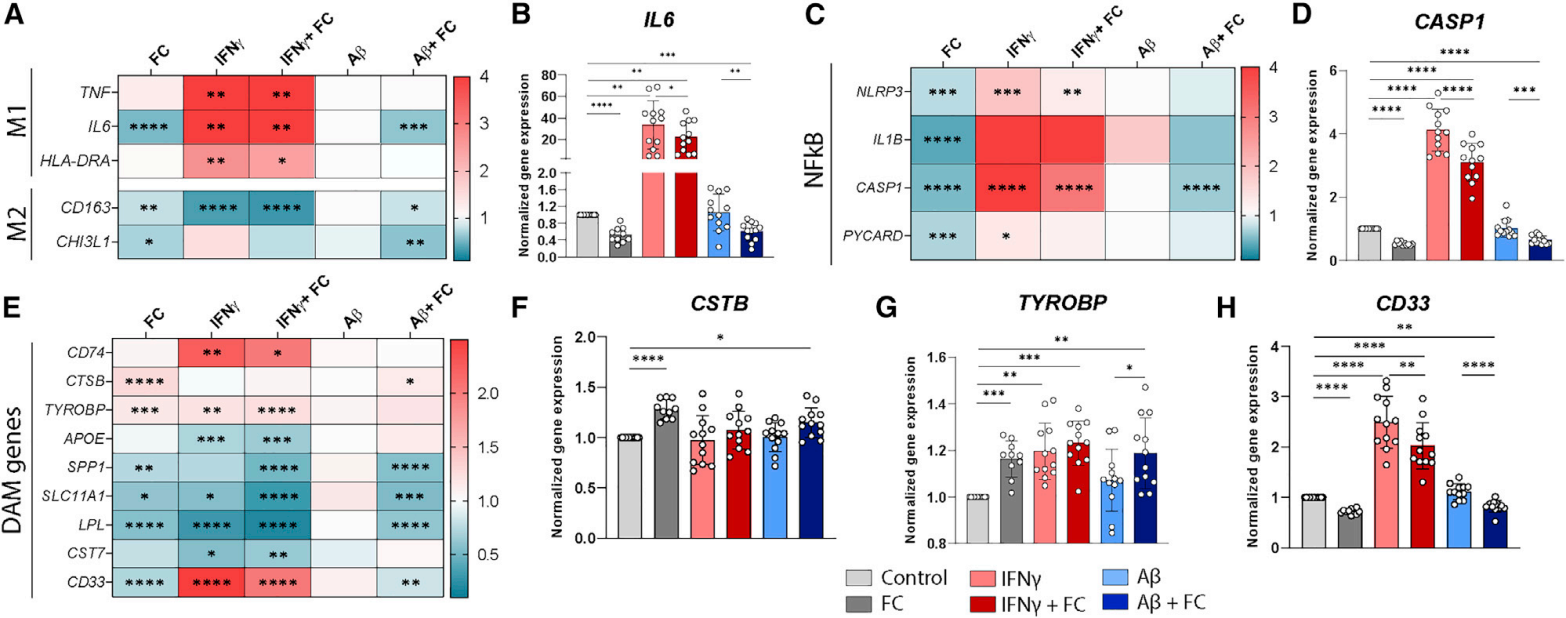
Iron treatment dampens pro- and anti-inflammatory activation (A) Heatmap of changes in expression of classic M1 and M2 genes assessed with qPCR showed classic M1 genes upregulated by IFN-γ, but no change or downregulation of both M1 (*TNF*, *IL6* , and *HLA-DRA*) and M2 genes (*CD163* and *CHI3L1)* after FC treatment. Aβ showed no significant changes. (B) Bar graph of *IL6.* (C) FC treatment also leads to downregulation of genes involved in NF-κB pathway *NLRP3*, *IL1B*, *CASP1* and *PYCARD*, whereas IFN-γ leads to significant upregulation of this immune response-regulating pathway. (D) Bar graph of *CASP1.* (E) Both IFN-γ and FC exposure, or a combination of the two, leads to mixed results in up- and downregulation of core DAM genes. (F–H) *CTSB* and *TYROBP* are significantly upregulated following FC exposure (F and G), whereas *SPP1*, *SLC11A1*, *LPL*, and *CD33* (H) are significantly downregulated. *APOE*, *CD74*, and *CST7* are affected by IFN-γ treatment. n = 12 per condition, 3 independent experiments for 4 iPSC lines. Statistical analysis was performed using a mixed-effects model with Geisser-Greenhouse correction for matched data and Sidak post hoc correction. All of the bar graphs indicate means ± SDs. ^∗^p < 0.05, ^∗∗^p < 0.01, ^∗∗∗^p < 0.001, ^∗∗∗∗^p < 0.0001.

In murine models, there have been conflicting reports of iron either inducing pro-inflammatory activation via the NF-κB pathway (Nnah et al., 2020) or inhibiting the pro-inflammatory response (Agoro et al., 2018; Gan et al., 2017). Therefore, using targeted gene expression profiling, we examined genes associated with the classic paradigm of pro-inflammatory (M1) and anti-inflammatory (M2) MG and the NF-κB pathway, with downstream NLR family pyrin domain containing 3 (NLRP3) inflammasome activation. As expected, IFN-γ induced a strong pro-inflammatory transcriptional signature, with increased gene expression of M1 markers *TNF*, *IL6*, *and HLA-DRA* and decreased *CD163* mRNA expression (Figures 4A and 4B). Conversely, iron treatment depressed both pro- and anti-inflammatory profiles, with significant downregulation of *IL6*, *CD163*, and *CHI3L1* mRNA expression (Figures 4A and 4B). This effect was also observed when FC was added in combination with IFN-γ or Aβ. Similarly, individual genes of the NF-κB pathway (*NLRP3*, *IL1B*, *CASP1*, PYCARD) were upregulated following IFN-γ, but decreased after FC exposure (Figures 4C and 4D). Since there was evidence of iron dyshomeostasis in the microglial DAM signature and the iron-metabolism genes *FTL* and *FTH1* have been considered as core genes in DAM MG (Keren-Shaul et al., 2017; Mathys et al., 2019), we explored whether FC treatment would affect DAM signature genes in iPSC-MG. We selected 8 upregulated genes (*CD74*, *CTSB*, *TYROBP*, *APOE*, *SPP1*, *SLC11A1*, *LPL*, *CST7*) and 3 downregulated genes (*CD33* and previously shown P2RY12 and *TMEM119*) of this DAM signature. None of our different treatments resulted in consistent gene expression profiles resembling DAM (Figure 4E). Interestingly, exclusively FC induced expression of the *CTSB* and *TYROBP* genes (Figures 4F and 4G), both relevant in AD. While some overlap with changes in the expression of DAM genes was present (*CTSB*, *TREM2*, *CD33*, *P2RY12),* iron treatment did not lead to the characteristic DAM signature. Bar graphs of all of the genes displayed in the heatmaps can be found in Figure S2.

### Transcriptomic analysis shows oxidative stress in iron-loaded iPSC-MG

**Figure 5:**
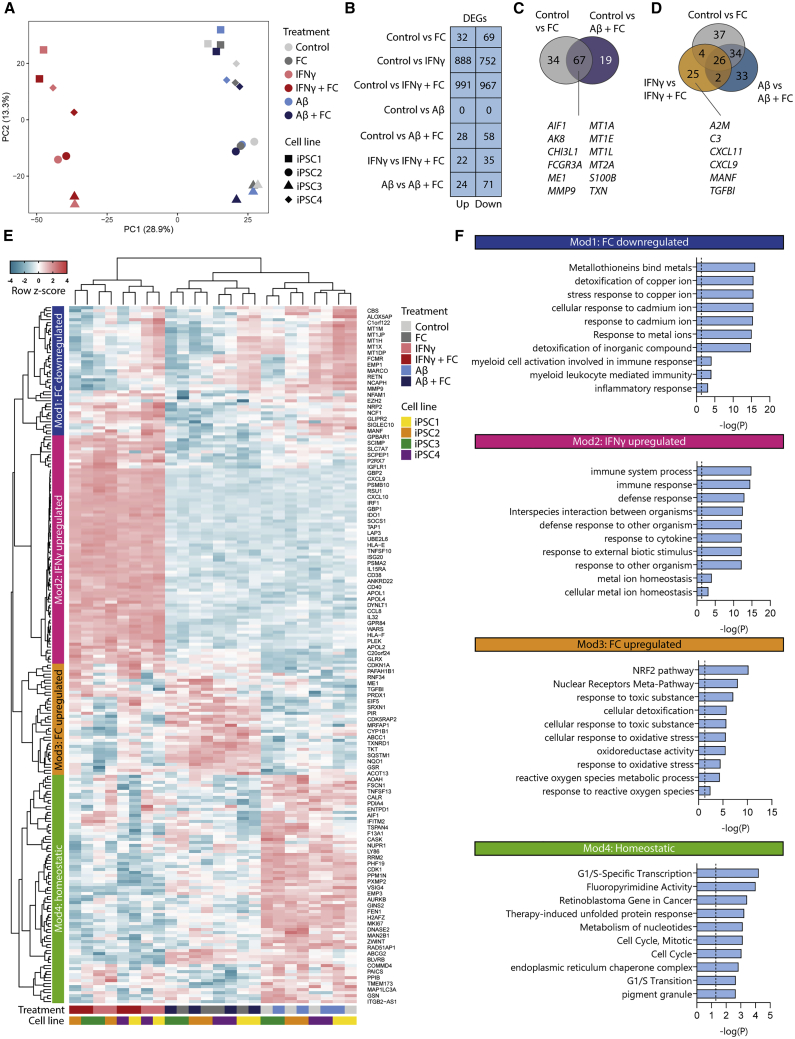
Transcriptomic analysis shows oxidative stress in iron-loaded iPSC-MG (A) Principal-component analysis shows clustering of samples based on IFN-γ treatment (PC1) and iPSC line (PC2) (n = 4 per treatment, 1 independent experiment for 4 iPSC lines). (B) Differentially expressed gene (DEGs) counts for each treatment. Statistical testing was performed using the Fisher’s exact test, adapted for overdispersed data with additive fitted model to correct for iPSC line variance. p values were corrected for false discovery rate (FDR), and a statistical threshold of FDR < 0.05 was applied. (C) Overlap of DEGs for FC- and Aβ + FC-treated cells. (D) Overlap of DEGs for all FC^−^- versus FC^+^-treated groups (D). Genes mentioned in the Venn diagram are included based on interest. (E) Heatmap with *Z* scores for the (top 60) DEGs of all of the treated groups. Hierarchical clustering identified 4 modules: FC downregulated (dark blue), IFN-γ upregulated (pink), FC upregulated (yellow), and homeostatic (green). (F) Gene Ontology enrichment analysis of the identified modules showed consistent immune response activation in IFN-γ-treated samples (Mod2; pink), but evidence of increased cellular and more specifically oxidative stress in FC-treated samples (Mod3; yellow). In addition, cellular response to heavy metals appeared downregulated in FC-treated samples (Mod1; dark blue), whereas homeostatic processes involving cell-cycle regulation were downregulated in all FC and IFN-γ-treated samples (Mod4; green). The dotted line indicates a significance threshold of p *<* 0.05.

As FC did not induce classic pro-inflammatory activation as was previously published, but rather depressed pro- and anti-inflammatory activation, we performed whole-transcriptome RNA-seq, to elucidate which pathways were activated following iron loading. Principal-component analysis (PCA) of all of the samples showed that the first principal component (PC1) defined stimulation with IFN-γ (Figure 5A, PC1), whereas PCA2 showed the clustering of iPSC lines, underscoring the heterogeneity between the iPSC lines (Figure 5A, PC2). Correspondingly, differential gene expression (DGE) analysis showed the greatest number of differentially expressed genes (DEGs) between control and IFN-γ or IFN-γ + FC-treated iPSC-MG, followed by control versus FC and control versus Aβ + FC (Figure 5B). All of the groups that included FC treatment showed considerable overlap in the identified DEGs (Figures 5C and 5D), which underscored the reliability of the identified DEGs. The DEGs included *MEI*, which is involved in regulating metabolic oxidative activity, and several genes of the metallothionein family (*MT1A*, *MT1E*, *MT1L*, and *MT2A)*, encoding cysteine-rich proteins that bind divalent heavy metal ions. In addition, we performed DGE analysis on all FC^−^ versus FC^+^ groups (Figure 5B) and again found considerable overlap between the observed DEGs (Figure 5D). Some DEGs were only observed in the IFN-γ versus IFN-γ + FC groups, which may suggest a possible synergistic effect between proinflammatory activation and iron loading; a variety of genes involved in immune response (*A2M*, *C3*), chemokine regulation (*CXCL* family), and cell adhesion (*TGFBI*) were observed. Hierarchical clustering based on all DEGs from the iron-treated groups (N = 57–101) and the top 60 DEGs from IFN-γ-treated groups led to the identification of 4 modules, which appeared coordinately regulated across treatments (Figure 5E and Table S4 for all treatment-enriched DEGs). Hierarchical clustering using the previously identified DEGs showed FC-treated samples (FC and FC + Aβ) to be highly similar and to separate from IFN-γ and IFN-γ + FC- and control and Aβ-treated samples. Module 1 (Mod1; dark blue) consisted of genes that were downregulated upon iron treatment and included the previously mentioned metallothionein family. Gene Ontology (GO) enrichment analysis confirmed that processes involved in the binding of metals or other responses to metal ions were most significantly downregulated (Figure 5F). Moreover, in line with the targeted gene expression analysis, several processes relating to immune activation of myeloid cells also appeared significantly downregulated. Genes from the second module, Mod2, were upregulated in IFN-γ-treated samples (Figure 5E, pink) and corresponded to pro-inflammatory/M1-associated pathways such as defense and immune responses against foreign organisms and cytokine response processes (Figure 5F, pink). Also, upregulated processes of metal ion homeostasis were observed, which corresponded to previously observed findings of increased labile iron influx following IFN-γ treatment (Figure 2E). The third module, Mod3, contained genes upregulated in FC-, Aβ + FC-, and IFNγ + FC-treated samples (Figure 5F, yellow). GO enrichment analysis revealed upregulated processes of response to toxic substances, oxidative stress, and ROS (Figure 5E, yellow). Likewise, NRF2 was the most significantly upregulated pathway, which is activated under oxidative stress conditions and activates antioxidative genes and proteins. The upregulation of NRF2 and cellular stress response pathways, as well as the downregulation of metal ion responses, were also confirmed with independent gene set Enrichment analysis (GSEA) on the log fold change of all genes between treatments (Figure S3). The full list of significant pathways identified with GO and GSEA, can be found in Tables S5 and S6, respectively. Finally, genes from the fourth module, Mod4, were downregulated in both IFN-γ- and FC-treated samples and corresponded to homeostatic processes such as cell-cycle regulation. Using whole-transcriptome RNA-seq, we could confirm that iPSC-MG treated with FC show a disparate activation pattern from the one induced by the pro-inflammatory cytokine IFN-γ. Moreover, we now identified pathways of cellular detoxification, oxidative stress, and downregulated homeostatic function in iron-loaded iPSC-MG.

### Iron affects phagocytosis in iPSC-MG

**Figure 6:**
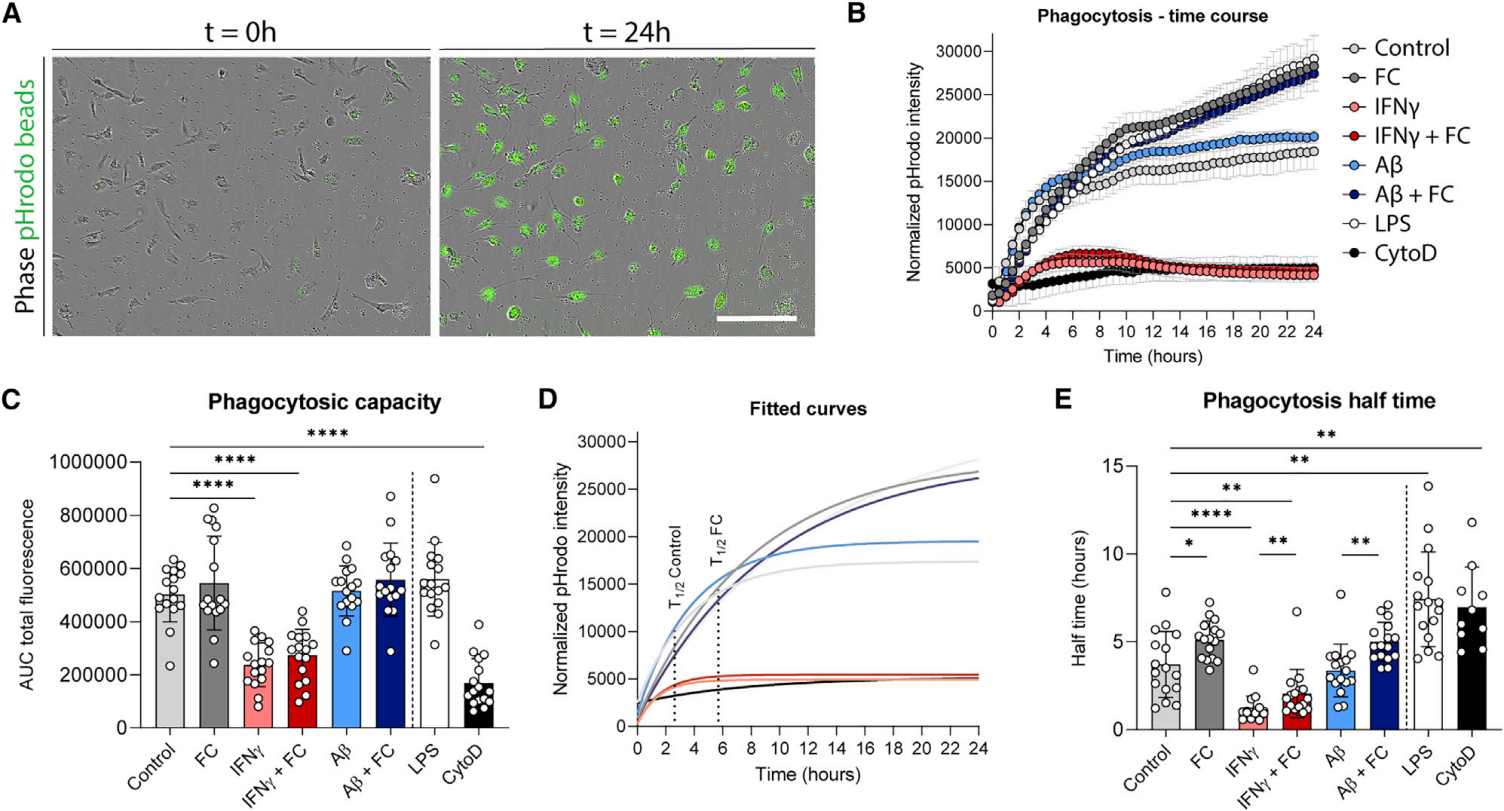
Slower phagocytosis following iron loading in iPSC-MG (A) Representative images of phagocytosed pHrodo zymosan beads at initial seeding and after 24 h. (B) Time course of phagocytosis across the 6 different conditions, and with a positive (LPS) and negative (cytochalasin D) control. Mean ± SD of 1 iPSC-MG line with 6 technical replicates. (C) Significant reduction of total phagocytic capacity following IFN-γ exposure, and a small, non-significant increase was observed following FC or LPS exposure. (D) Estimated slope of phagocytosis time course in (B), from which half-time, the time it takes to reach half their maximum phagocytic capacity, can be calculated. Shown for control and FC. (E) Phagocytosis half-time is significantly increased in each condition with FC and decreased after IFN-γ. (C andE) n = 16 per condition, 3–5 independent experiments for 4 iPSC lines. Statistical analysis was performed using a mixed-effects model with Geisser-Greenhouse correction for matched data and Sidak post hoc correction. Scale bars, 200 μm. All of the bar graphs indicate means ± SDs. ^∗^p < 0.05, ^∗∗^p < 0.01, ^∗∗∗^p < 0.001, ^∗∗∗∗^p < 0.0001.

In addition to transcriptomic and protein changes, we explored the effect of iron on phagocytosis, which is one of the key functions of innate immune cells such as microglia. After treatment with our six previously defined interventions and an additional positive and negative control (lipopolysaccharide [LPS] and cytochalasin D, respectively), we added pHrodo zymosan beads to the media and performed live cell imaging every 30 min for 24 h to assess the phagocytic speed and maximum capacity of the iPSC-MG (Figure 6A). IFN-γ greatly reduced the total phagocytic capacity of iPSC-MG, almost to the level of our negative control cytochalasin D (Figure 6B). FC, however, induced a slight increase in total phagocytic capacity, almost similar to that of LPS (Figure 6B). However, the initial speed of phagocytosis was reduced in all FC-treated groups (Figure 6B). We quantified the half-time by first performing a nonlinear regression curve fitting with an exponential one-phase association and subsequently calculating the time it takes for the iPSC-MG to reach half of the maximum phagocytic capacity based on this fitted curve (Figure 6D). This showed that FC treatment significantly increased phagocytosis half-time (Figure 6E).

### Increased iron impairs metabolic activity of iPSC-MG

**Figure 7:**
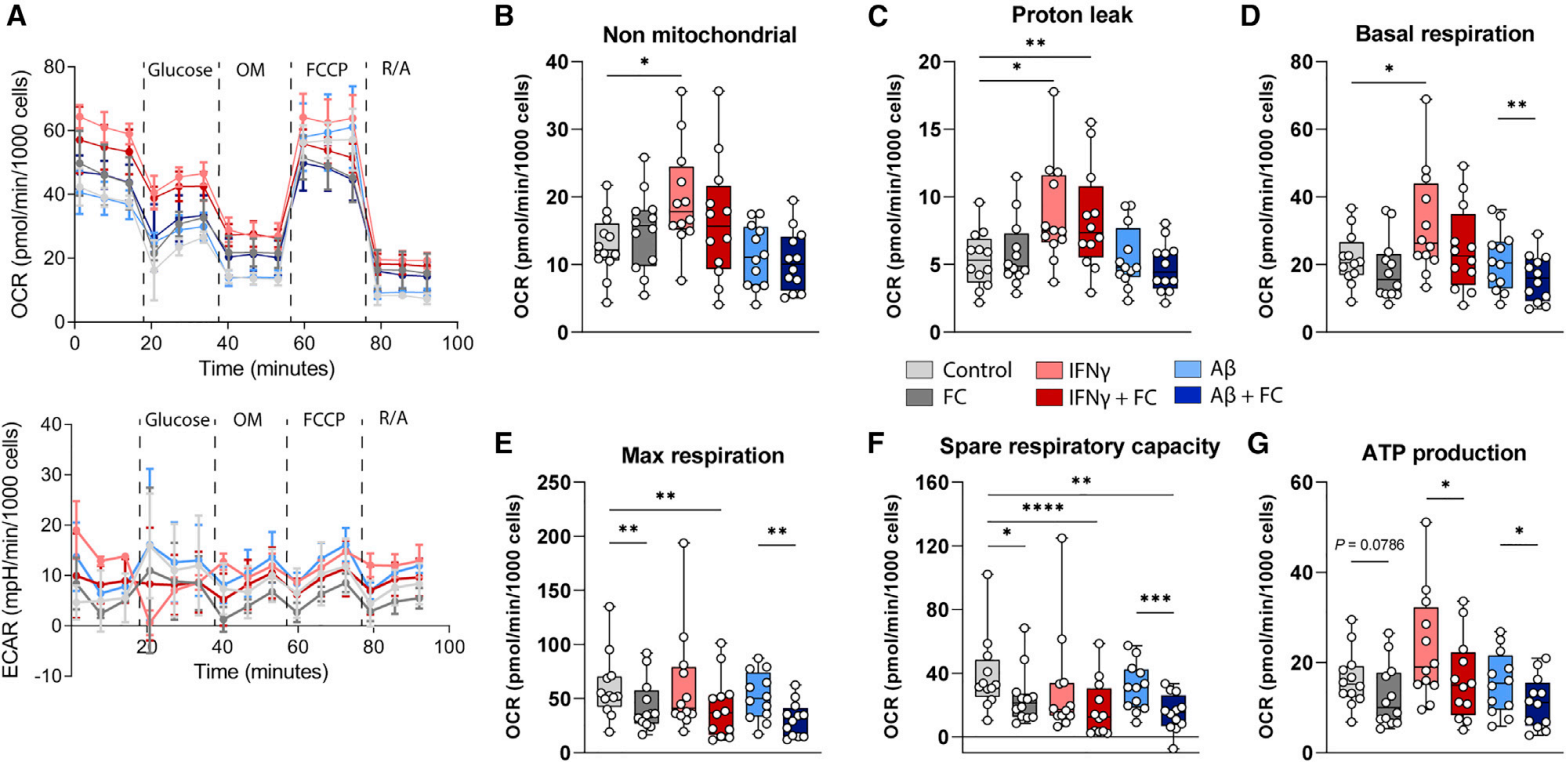
Iron loading affects metabolism of iPSC-MG (A) Representative oxygen consumption rate (OCR; top) and extracellular acidification rate (ECAR; bottom) curves following treatments show proper oxidative respiration (OCR), but minimal anaerobic glycolysis (ECAR). Mean values ±SDs are plotted (n = 4 technical replicates). (B–D) IFN-γ-treated iPSC-MG showed increased non-mitochondrial respiration, proton leak, and basal respiration, whereas FC treatment resulted in minimal changes. (E–G) Treatment with FC resulted in decreased maximum respiration, spare respiratory capacity, and ATP production, for all FC^+^ vs FC^−^ groups. (B–G) Statistical testing using repeated measures (RM) 1-way ANOVA with Geisser-Greenhouse correction was performed for matched data, and a post hoc Sidak correction was performed. n = 12 per condition, 2–4 independent experiments for 4 iPSC lines. Boxplots display median with interquartile ranges and maximum and minimum. ^∗^p < 0.05, ^∗∗^p < 0.01, ^∗∗∗^p < 0.001, ^∗∗∗∗^p < 0.0001.

To assess the effect of iron loading on the bioenergetics of iPSC-MG, we measured both the oxygen consumption rate (OCR) and extracellular acidification rate (ECAR) to assess mitochondrial metabolism and glycolysis, respectively, after treatment with our six groups of compounds. The iPSC-MG showed the expected response to electron transport chain modulators and showed proper oxidative phosphorylation, as measured with OCR, but only very little glycolysis, as measured with ECAR (Figure 7A). The quantification of the OCR curves showed a significant increase in non-mitochondrial respiration, proton leak, and basal respiration following IFN-γ exposure (Figures 7B–6D). FC treatment led to reduced maximum respiration, spare respiratory capacity, and subsequent ATP production when comparing FC^+^ versus FC^−^ conditions (Figures 7E–7G). This indicates that IFN-γ treatment induces a switch from oxidative respiration toward anaerobic glycolysis, often associated with an M1 pro-inflammatory phenotype, which is in line with transcriptomic data (Figures 4A and 5F). Iron appeared to influence electron transport chain function, resulting in lower maximum and spare respiratory capacity rates following treatment with the uncoupler carbonyl cyanide 4-(trifluoromethoxy)phenylhydrazone (FCCP). Although neither indicate cellular dysfunction per se, it has been suggested that both higher maximum and spare respiratory capacity exist so that cells can appropriately respond to increases in demand and withstand periods of stress (Divakaruni et al., 2014). Decreased levels would therefore indicate cellular stress and increased susceptibility to cell death.

## Discussion

In this study, we showed that exposure to an increasing concentration of iron results in ferritin iron loading in iPSC-MG, independent of inflammatory activation by IFN-γ or Aβ. Iron-loaded iPSC-MG showed both morphological and transcriptomic activation, but dampened both classic pro- or anti-inflammatory activation patterns. Instead, the NRF2 and other oxidative stress pathways were activated. Functionally, the rate of phagocytosis was reduced, and iron treatment resulted in decreased maximum and spare respiratory capacity of mitochondrial metabolism.

We showed that treatment of iPSC-MG with FC resulted in significantly increased labile iron, as did treatment with IFN-γ, while Aβ had no effect. Conversely, only FC treatment and not IFN-γ treatment resulted in the increased expression of the iron-storage protein ferritin. These results are in line with previous results in murine immortalized microglia, in which treatment with FAC induced significant iron uptake and increased ferritin expression (McCarthy et al., 2018). However, contrary to our findings, they observed an increase in divalent metal transporter 1 (DMT1) and heavy-chain ferritin expression in this cell line in response to pro-inflammatory activation with LPS, which we did not observe in iPSC-MG following pro-inflammatory activation with IFN-γ stimulation. Correspondingly, light-chain ferritin levels remained unaltered after inflammatory activation. Therefore, although the expression of ferritin is often considered the result of pro-inflammatory activation, our results in iPSC-MG indicate that the ferritin response is predominantly mediated by iron rather than by inflammatory activation.

There have been conflicting results regarding the activation status of microglia following iron treatment. A separate study using a murine immortalized microglia cell line found activation of the NF-κB pathway with downstream pro-IL-1β production following treatment with iron and Aβ (Nnah et al., 2020). Similarly, FAC treatment induced IL-1β production in murine isolated peripheral monocytes (Nakamura et al., 2016). In contrast, other studies found that iron inhibited the polarization toward pro-inflammatory M1 macrophages induced by either LPS or IFN-γ, but instead induced the anti-inflammatory M2 phenotype (Agoro et al., 2018; Gan et al., 2017). Our study is the first to test the response to iron using human iPSC-MG, and we found that iron treatment depresses both pro- and anti-inflammatory activation. Targeted gene expression analysis showed that treatment with FC (alone or in combination with IFN-γ) dampened the M1 phenotype, with downregulated NF-κB pathway genes *NLRP3*, *IL1B*, *CASP1*, and *PYCARD* and downregulated M1 genes tumor necrosis factor (TNF), *IL6*, and *HLA-DRA*. However, we also observed the downregulation of the M2 genes *CD163* and *CHI3L1*. RNA-seq analysis confirmed the downregulation of immune activation in iron-treated human iPSC-MG. All in all, the homeostatic function of iPSC-MG is clearly affected by iron treatment, but rather than classic pro- or anti-inflammatory activation, microglia appeared oxidatively stressed. In line with this, our bioenergetics analysis data did not indicate a switch toward glycolysis, as would be expected under pro-inflammatory conditions and observed after IFN-γ treatment, but rather suggested lower metabolic capacity, which is indicative of cellular stress. These findings are in agreement with a study by Yauger et al. (2019), in which they used immortalized rat instead of murine microglia, and showed increased ROS production following iron treatment, without an accompanying alteration in pro-inflammatory/M1 polarization markers. This study also highlights the discordance between species.

We also investigated the effect of Aβ, alone and in combination with iron, but found no direct effect of Aβ. To date, conflicting results exist on the effect of Aβ, which appears to depend on the model in which the results were obtained. Previous studies in murine MG showed the activation of NLRP3 and increased IL-1β production following treatment with oligomeric Aβ via Toll-like receptors (TLRs), which was exacerbated by iron (Burm et al., 2015; Nnah et al., 2020). Conversely, a recent study investigating TLR-meditated NLRP3 inflammasome activation in human iPSC-MG showed that contrary to other proteins, soluble oligomeric Aβ did not induce NLRP3 activation and subsequent IL-1β production (Trudler et al., 2021). Also in line with our results, another study using a human iPSC-MG model found almost no difference after treatment with oligomeric Aβ in either chemokinesis or phagocytic function (Konttinen et al., 2019). Therefore, murine and human microglia may differ too much to allow for valid comparisons. However, considering that Aβ is a notoriously difficult protein to study due to its self-aggregating properties and function that heavily depends on its conformation (Ladiwala et al., 2012), the opposing findings could also be due to technical differences across studies. Moreover, as mentioned briefly in the Results section, no effect was seen of the *APOE* genotype, despite previous reports showing a clear effect of *APOE* genotype on the function of iPSC-MG (Konttinen et al., 2019; Lin et al., 2018). However, in our case, we were likely underpowered to detect an effect, as we used only 4 non-isogenic lines harboring the *APOE3/3* and *APOE3/4* genotypes, as opposed to the isogenic *APOE3/3* and *APOE4/4* pairs as used in the previous studies.

From clinical *in vivo* MRI and post-mortem liquid crystal polymer-mass spectrometry studies, it has been suggested that iron levels correlate with accelerated cognitive decline, not only in AD (Ayton et al., 2017, 2020, 2021) but also in PD and amyotrophic lateral sclerosis (ALS) (Devos et al., 2019; Thomas et al., 2020), which increases the likelihood of a pathology-independent common pathway being responsible for the observed clinical effect. A previous study showed that iron could induce a dose-dependent increase in ROS production via nicotinamide adenine dinucleotide phosphate (NADPH) oxidases, which affected neuronal survival in a co-culture (Yauger et al., 2019). Similarly, we also found the NADPH pathway to be among the most significantly upregulated pathways in the iron-treated iPSC-MG. In addition, iron accumulation may contribute to neurodegeneration via ferroptosis, the iron-dependent cell-death pathway (Dixon et al., 2012), which has been found to play an important role in MG (Kapralov et al., 2020).

Although homogeneous cultures of iPSC-MG are a powerful and suitable model to study the cell-intrinsic effect of single or different combinations of stimuli, there are also several limitations. First, iPSC resemble immature developing microglia rather than mature or even degenerating microglia as associated with NDs. Second, the conditions used in this study cannot reflect the complex mix of cues that MG receive under diseased conditions, and potential synergistic effects will be missed.

Although iron accumulation and microglia activation are considered hallmarks of disease of many NDs, and evidence of altered microglial iron metabolism is found in both immunohistochemistry (IHC) and transcriptomic studies, the direct effect of iron on human MG had not been previously studied. Here, we show that microglial iron metabolism alterations reflect iron levels rather than inflammatory activation. Moreover, treatment with iron resulted in transcriptomic activation with signs of cellular detoxification and oxidative stress, together with impaired metabolic metabolism and altered phagocytic function. Further investigation is required to dissect the effects of these changes on microglial function, the interaction with other neural cell types, and the potential impact on disease progression.

## Experimental procedures

### FC, IFN-γ, and Aβ treatment

FC (Sigma-Aldrich) was dissolved in sterile H_2_O at 10 mM concentration 48 h in advance and put in a spinning rotor. Ascorbate (Sigma-Aldrich) was dissolved in sterile water at 500 mM concentration. A total of 100 μg hexafluoro-2-propanol (HFIP)-prepared Aβ (Bachem) was reconstituted in 10 mL pure DMSO, sonicated in a water bath for 10 min, further diluted in 90.8 μL phenol-red free HAM/F12, and incubated for 24 h at 4°C to obtain 200 μM oligomeric Aβ, as previously described (McCarthy et al., 2016). Ascorbate was added to media containing 50 μM FC to ascertain the reduction of the iron toward Fe^2+^, which can be transported into the cell via DMT1 importers. FC concentration was chosen based on the literature and the clear iron-loading effect seen on western blot and labile iron imaging (Figures S1D and S1E). Similarly, the final media was depleted of N2-supplement, as N2 contains high concentrations of transferrin, the apotransferrin of which can bind to the additional free iron. We tested whether the omission of the N2-supplement for the final 24 h resulted in transcriptomic activation of the iPSC-MG itself, which was not the case (Figure S1C). Aβ and IFN-γ (Peprotech) were added at 1 μM and 20 ng/mL, respectively, to media without N2-supplement, based on concentrations used in Nnah et al. (2020) and Holland et al. (2018), respectively. For all assays, all 6 treatments (control, FC, IFN-γ, IFN-γ + FC, Aβ, Aβ + FC) were performed in parallel via a complete change of media with the appropriate treatment group. iPSC-MG were treated for 24 h before subsequent assays were performed (i.e., lysate collection for WB, paraformaldehyde (PFA) fixation, RNA collection, labile iron imaging, phagocytosis assay, or seahorse metabolism assay). Further details on these assays can be found in the supplemental experimental procedures.

### Statistics

All of the data were inspected for being gaussian distributed. If normally distributed, data plots represent the mean and the standard deviation, while not normally distributed data are shown using the median with corresponding interquartile range, which is stated in each figure legend. A comparison of two continuous variables was performed using a two-tailed unpaired Student’s independent t test (normally distributed). A comparison of data from the 6 treatments, when normally distributed, was performed using a one-way ANOVA or mixed-effects model (in case of missing values) with Geisser-Greenhouse correction to adjust for the lack of sphericity, and followed up with a post hoc Sidak multiple comparisons test. Not normally distributed data were compared using a nonparametric Kruskal-Wallis test, followed with Dunn’s multiple comparisons test. A significance level of p < 0.05 was used. All of the statistical tests were performed using GraphPad Prism (version 8.00). For DGE of the RNA-seq data, we used the edgeR package (version 3.32.1) in R (version 4.0.5). Differential expression was assessed for each gene using an exact test analogous to Fisher’s exact test, but adapted for overdispersed data. We adjusted for any baseline differences between the different iPSC lines by fitting an additive model to make the comparison between treatments more precise.

A detailed description of all of the experimental procedures and all of the reagents, software, and machines that were used can be found in the supplemental information and Table S2).

## Author contributions

Conceptualization, B.K., L.v.d.W., and W.M.C.v.R.-M.; investigation, B.K., M.v.E., D.A.P., and Y.A.; methodology, B.K., D.A.P., and P.B.; data curation, B.K.; formal analysis, B.K.; funding acquisition, B.K., L.v.d.W., and W.M.C.v.R.-M.; supervision, J.P., L.v.d.W., and W.M.C.v.R.-M.; Writing – original draft, B.K., L.v.d.W., and W.M.C.v.R.-M.; Writing – review & editing, all of the authors.

## Conflicts of interests

The authors declare no competing interests. All of the co-authors have seen and agree with the contents of the manuscript.

## Data Availability

The authors confirm that the data supporting the findings of this study are available within the article and/or its supplemental information. Raw RNA-seq data generated in this paper are available through EGAS00001006112. The R script used for the analysis of the RNA-seq data will be shared upon request. Reanalyzed datasets for this study are available through GSE135707 and GSE89189.

## Supplemental information

Document S1. Figures S1–S4, Tables S1–S3, and supplemental experimental procedures

Table S4. DEG result, related to Figure 5. All identified differentially expressed genes (DEGs) between treatment groups reported alongside their log foldchange (logFC), log counts per million (logCPM), F-statistic (F), p-value and false discovery rate (FDR)

Table S5. GO analysis results, related to Figure 5. All identified significantly affected GO terms in the 4 different identified modules, on which Figure 5F is based

Table S6. GSEA analysis results, related to Figure 5. All overrepresented classes of genes identified using gene set enrichment analysis (GSEA), on which Figure S3 is based

Document S2. Article plus supplemental information
