# Supplementary material for: Iron accumulation induces oxidative stress, while depressing inflammatory polarization in human iPSC-derived microglia
Source: Stem Cell Reports. 2022 May 5;17(6):1351–65. doi: 10.1016/j.stemcr.2022.04.006 (PMC9213827; doi:10.1016/j.stemcr.2022.04.006)
Supplement: Document S1. Figures S1–S4, Tables S1–S3, and supplemental experimental procedures [file mmc1.pdf]

Stem Cell Reports, Volume *17*

Supplemental Information

Iron accumulation induces oxidative stress, while depressing inflammatory polarization in human iPSC-derived microglia

Boyd Kenkhuis, Michelle van Eekeren, David A. Parfitt, Yavuz Ariyurek, Poulomi Banerjee, Josef Priller, Louise van der Weerd, and Willeke M.C. van Roon-Mom

Supplementary Information

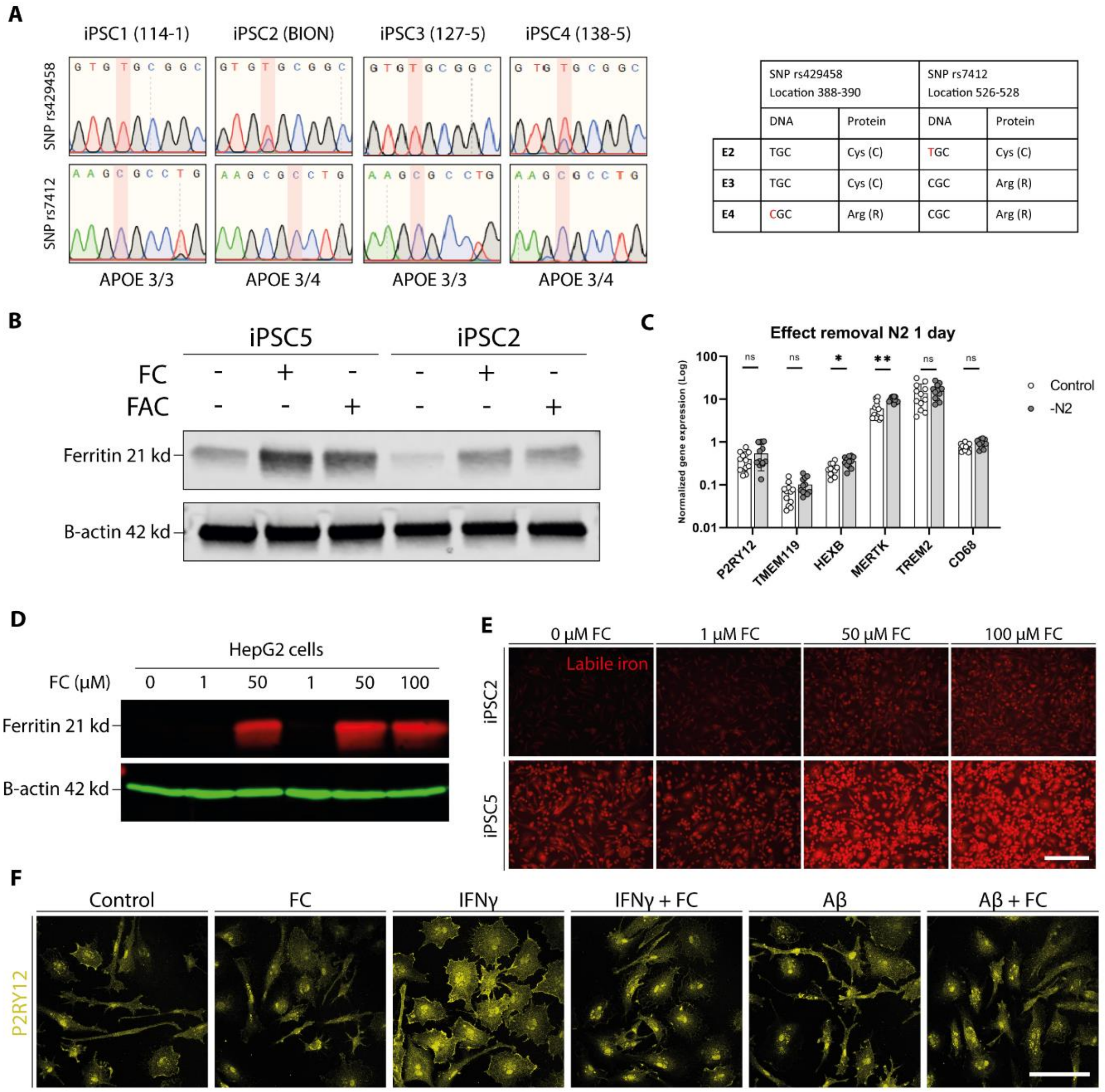

Fig. S1 iPSC and iron loading validation, related to Figure 1 and 2. A *APOE* genotyping of iPSC-lines **B** Western blot for ferritin following ferric citrate (FC) and ferric ammonium citrate (FAC) treatment **C** qPCR analysis of expression of core microglia genes prior to and after 24h N2 removal **D** Western blot for ferritin following FC treatment at different doses with HepG2 cells **E** Representative images of live cell imaging of labile iron showing increased cytosolic iron after 50 and 100 µM, but not after 0 or 1 µM. Scale bar 200 µm **F** P2RY12 expression is increased following IFNγ treatment on immunofluorescence. Scale bar 100 µm

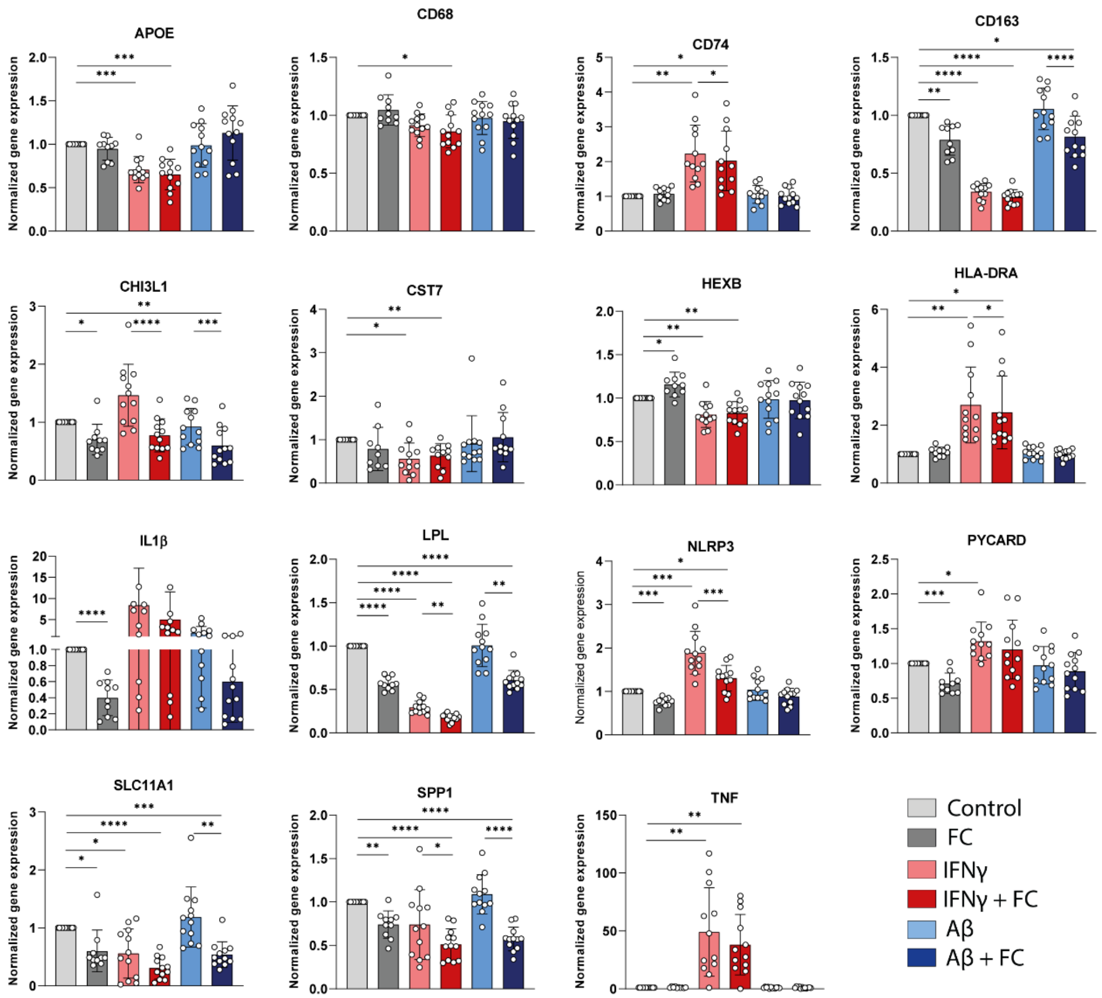

Fig. S2 Bargraphs of targeted gene-expression analysis using the Dynamic Array (Fluidigm), related to Figure 4.

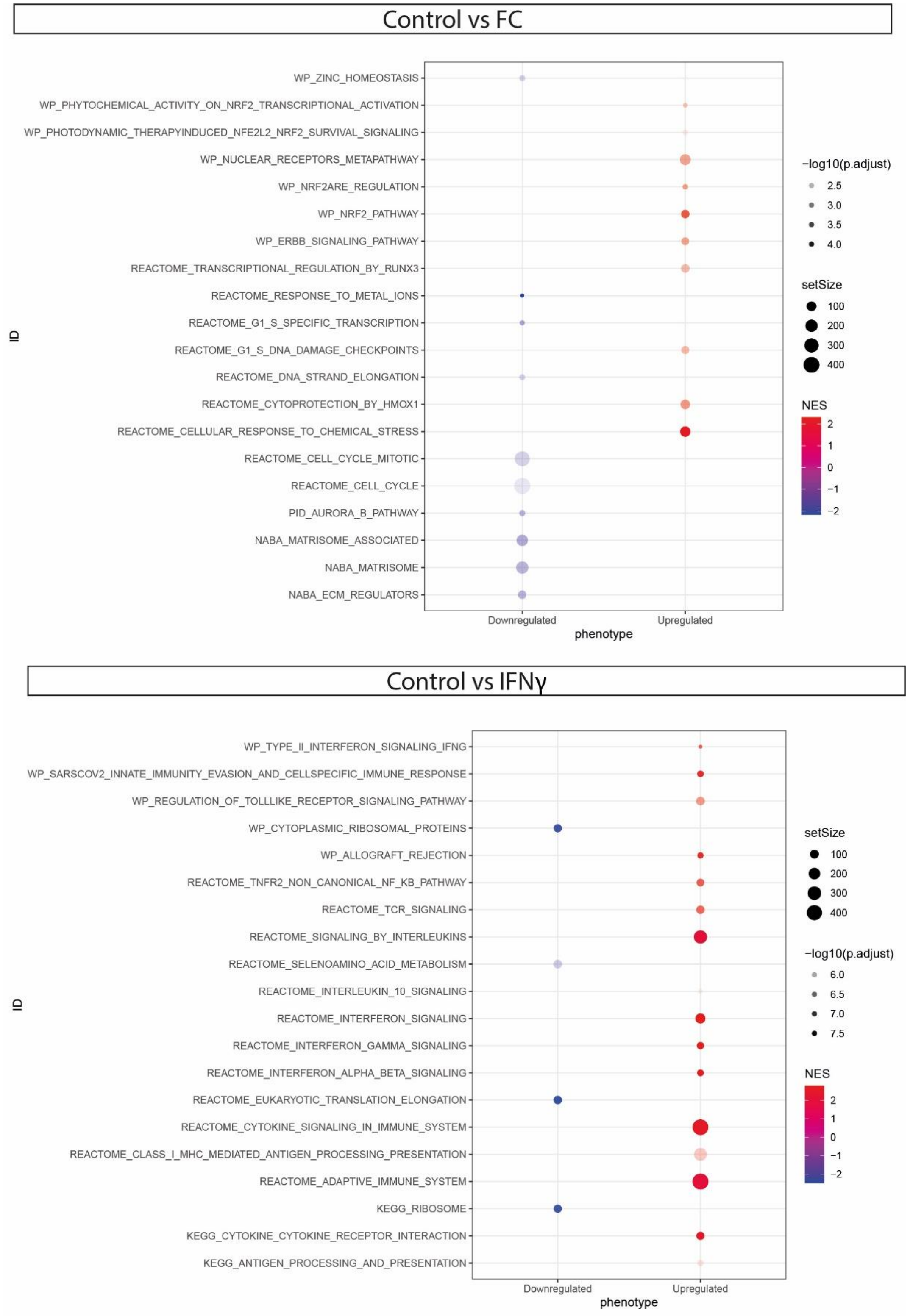

Fig. S3 GSEA analysis results. Legend on next page.

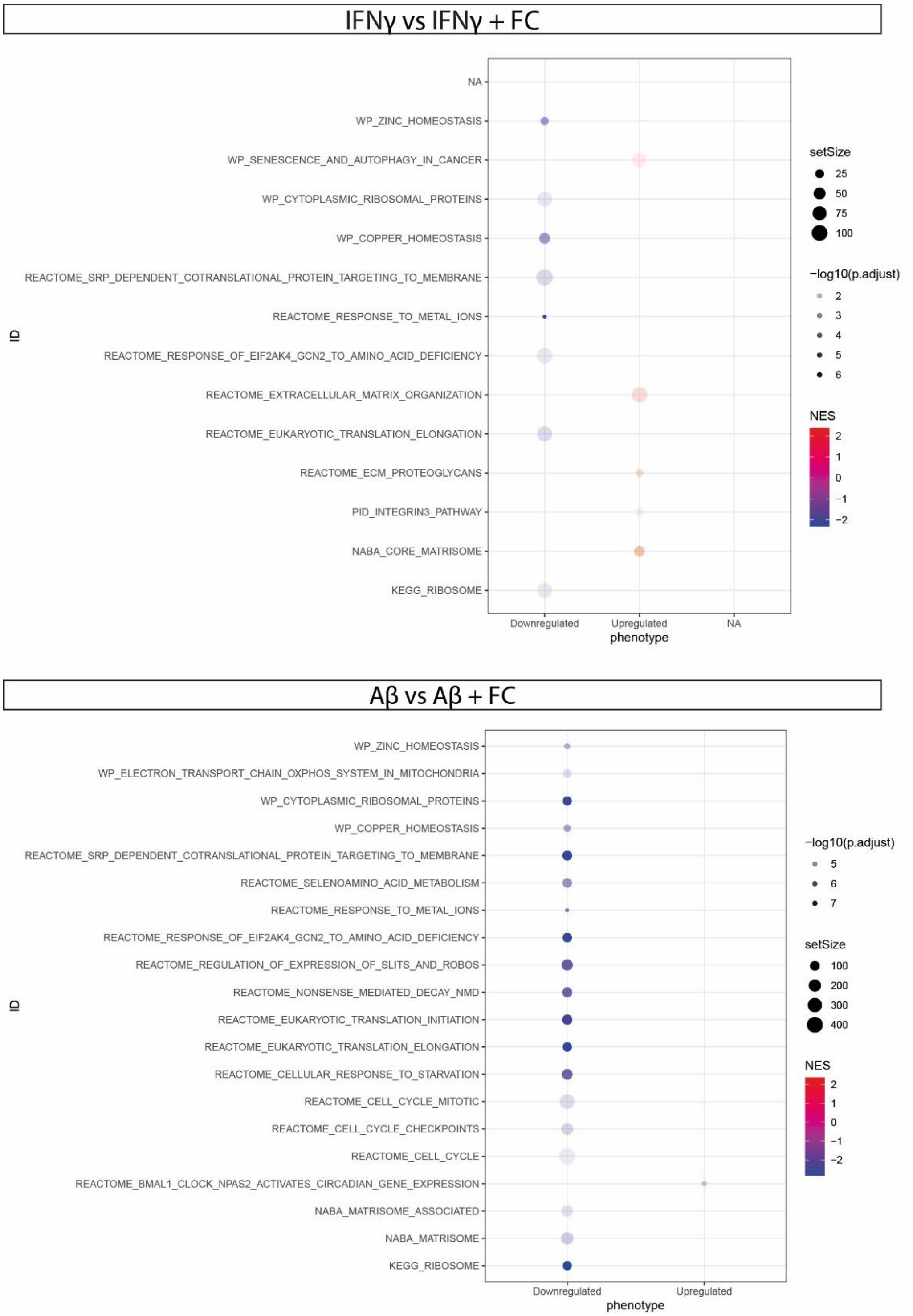

Fig. S3 GSEA analysis results, related to Figure 5. GSEA results from control vs FC and control vs IFNγ show excellent overlap with identified GO terms as was highlighted in the paper. However, although the differentially expressed genes showed considerable overlap between control vs FC and Aβ vs Aβ + FC, considering Aβ had little effect, we do see differences in identified GSEA pathways. These differences are primarily in the omission of the upregulated pathways concerning oxidative stress. The identified downregulated pathways in metal ion metabolism do overlap and appear to be more robust.

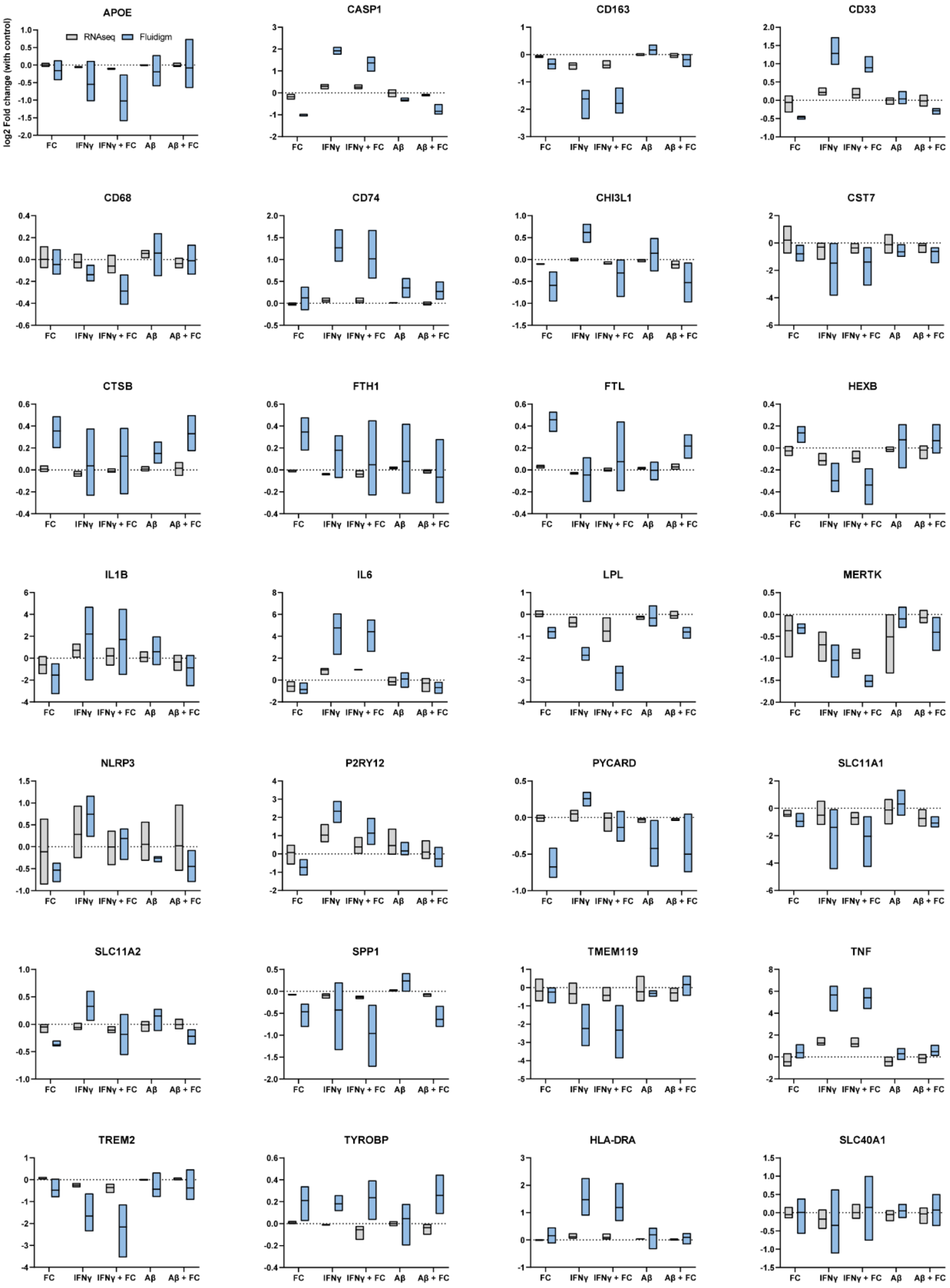

Fig. S4 Comparison log2 fold changes RNAseq (grey) vs fluidigm targeted gene expression analysis (blue), related to Figure 4 and 5

Table S1. Cell line characteristics, related to Figure 1

Alias	Age, y	Sex	Clinical Details	ApoE status	Reprogramming method	Cell line id	Reference
iPSC1	49	F	control	ApoE 3/3	Non-integrating Sendai virus	114-1	Buijsen et al. 2018 https://doi.org/10.1016/j.scr.2018.03.018
iPSC2	15-19	M	control	ApoE 3/4	Non-integrating Episomal	BIONi010-C	Commercial: Ebisc https://cells.ebisc.org/BIONi010-C/
iPSC3	44	F	control	ApoE 3/3	Non-integrating Sendai virus	127-5	In house characterization
iPSC4	45	M	control	ApoE 3/4	Non-integrating Sendai virus	138-5	In house characterization

Table S2. Resources, related to experimental procedures

Product	Source	Identifier
**iPSC culturing**		
Matrigel	Corning	#734-1440
mTESR	STEMCELL Technologies	#85870
37 µm Reversible Strainer, large	STEMCELL Technologies	#27250
Trypan Blue Solution, 0.4%	Invitrogen	#15250-061
AggreWell800	STEMCELL Technologies	#34815
AggreWell Rinsing Solution	STEMCELL Technologies	#07010
Accutase	STEMCELL Technologies	#07920
ReLesR	STEMCELL Technologies	#05872
Glutamax	ThemoFisher	#35050-038
Pen/Strep (100x)	Invitrogen	#15140122
2-mercaptoethanol	ThemoFisher	#31350-010
N2-supplement	ThemoFisher	#17502-048
X-VIVO 15	Lonza	BE02-060F
Advanced DMEM/F12	ThemoFisher	#12634-010
Poly-D-lysine hydrobromide	SIGMA-ALDRICH	#P0899-50MG
2% Gelatin solution	SIGMA-ALDRICH	G1393-100mL
**Growth factors**		
Recombinant Human VEGF	Peprotech	100-20-B
Human SCF Recombinant Protein	ThermoFisher	PHC2115
Human BMP4 Recombinant Protein	ThermoFisher	PHC9534
Recombinant Human M-CSF	Peprotech	#300-25
Recombinant Human IL-3	Peprotech	#200-03
Recombinant human IL-34	Peprotech	#200-34
Recombinant human GM-CSF	Peprotech	#300-03
**Treatment substances**		
Human IFN-γ - 100ug	Peprotech	300-02-B
Ferric Ammonium Citrate	Sigma-Aldrich	RES20400-A702X
Ferric Citrate (FC)	Sigma-Aldrich	F3388-250G
Ascorbate	Sigma-Aldrich	PHR1279-1g
Amyloid β-Protein (1-42) (HFIP-treated)	Bachem	4090148.01
Amyloid β-Protein (42-1) (HFIP-treated)	Bachem	4107743.01
Cytochalasin D - 1 mg	ThermoFisher	PHZ1063
Lipopolysacharide (LPS) (From E. Coli O111:B4)	Sigma-Aldrich	L4391-1MG

**Antibodies**		
MAP2 (Rabbit)	ThermoFisher	PA17646
Iba1 (Goat)	Abcam	ab5076
TMEM119 (Rabbit)	Abcam	ab185333
P2RY12 (Rabbit)	Sigma-Aldrich	HPA014518
Ferritin (rabbit)	Abcam	ab75973
Actin (beta) (AC-15)	Novus biologicals	NB600-501
Donkey anti-Goat IgG (H+L) Highly Cross-Adsorbed Secondary Antibody, Alexa Fluor Plus 488	ThermoFisher	A32814
Donkey anti-Rabbit IgG (H+L) Highly Cross-Adsorbed Secondary Antibody, Alexa Fluor Plus 594	ThermoFisher	A32754
goat anti-Rabbit IRDye 800CW	LI-COR	926-32211
goat anti-Mouse IRDye 680CW	LI-COR	926-68070
**IHC/Western Blot/Live cell assays**		
DAPI (1mg)	Sigma-Aldrich	D9542
prolong diamond	ThermoFisher	P36961
cOmplete™ ULTRA Tablets, Mini, EASYpack Protease Inhibitor Cocktail	Roche	5892970001
Pierce™ BCA Protein Assay Kit	ThermoFisher	23225
4–20% Mini-PROTEAN® TGX™ Precast Protein Gels	BIO-RAD	456-1094
Trans-Blot Turbo RTA Mini PVDF Transfer Kit, for 40 blots	BIO-RAD	1704272
FerroOrange	Goryo Chemical	GC904-01
pHrodo Green Zymosan Bioparticles	ThermoFisher	P35365
Opti-MEM I Reduced Serum Medium - 100 ml	Gibco	31985-062
CELL CULTURE MICROPLATE, 96 WELL, PS, F-BOTTOM (CHIMNEY WELL), µCLEAR	Greiner Bio-One	655090
XFe96 FluxPak	Seahorse Biosciences	102416-100
XF Base medium	Seahorse Biosciences	102353-100
D-Glucose 45%	Sigma-Aldrich	G8769
Rotenone	Sigma-Aldrich	D8375
Antimycin A	Sigma-Aldrich	A8674-25MG
FCCP	Sigma-Aldrich	C2920-10 MG
Oligomycin A	Cayman Chemical	11342-5MG
**DNA/RNA/PCR kits**		
ReliaPrep™ RNA Miniprep Systems	Promega	Z6012
ReliaPrep™ gDNA Tissue Miniprep System	Promega	A2052
Transcriptor First Strand cDNA synthesis Kit	Roche	Cat. No. 04 897 030 001
NucleoSpin PCR clean-up kit	Macherey-Nagel	740609.25
KAPA HiFi HotStart ReadyMix	Roche	7958927001
Qubit dsDNA HS Assay Kit	ThermoFisher	Q32854
KAPA HyperPlus Kit	Roche	07962401001

**Software**		
Graphpad Prism (Version 8.00)	Graphpad	https://www.graphpad.com/
LAS X	Leica	www.leica-microsystems.com
Image studio lite (Version 5.2)	LI-COR	https://www.licor.com/bio/image-studio-lite/
Fiji (is ImageJ)	NIH	https://fiji.sc/
Adobe Illustrator 2021	Adobe	https://www.adobe.com/products/illustrator
R (Version 4.0.3)		https://www.R-project.org/.
Rstudio (Version 1.4.1717)		http://www.rstudio.com/
Nf-core/rnaseq (Version 3.2)	Ewels et al. 2019 (Ewels et al., 2019)	https://nf-co.re/rnaseq/3.2/usage
edgeR (Version 3.32.1)	Robinson et al. 2010 (Robinson et al., 2010)	https://bioconductor.org/packages/release/bioc/html/edgeR.html
Gplots (Version 3.1.1)	Warnes et al. 2020	https://CRAN.R-project.org/package=gplots
GSEABase (Version 1.52.1)	Morgan et al. 2021	https://bioconductor.org/packages/release/bioc/html/GSEABase.html
clusterProfiler (Version 3.18.1)	Yu et al. (2021) (Yu et al., 2012)	https://bioconductor.org/packages/release/bioc/html/clusterProfiler.html
gprofiler2 (Version 0.2.0)	Kolberg and Raudvere	https://cran.r-project.org/web/packages/gprofiler2/index.html
matrixStats (Version 0.58.0)	Bengtsson et al. 2021	https://CRAN.R-project.org/package=matrixStats
Tidyverse (Version 1.3.1)	Wickham et al. 2019 (Wickham et al., 2019)	https://tidyverse.tidyverse.org/
Biobase (Version 2.50.0)	Huber et al. 2015 (Huber et al., 2015)	https://bioconductor.org/packages/release/bioc/html/Biobase.html
**Other**		
ND-1000 Spectrophotometer	NanoDrop	
TCS SP8 confocal microscope	Leica	
CellInsight CX7 High-Content Screening platform	ThermoFisher	
BZ-X800 All-in-one fluorescence microscope	Keyence	
IncuCyte live cell imaging platform	Essen Bioscience	
Odyssey Clx	LI-COR	
Trans-Blot Turbo Transfer system	BIO-RAD	
Bioruptor Pico	Diagenode	
Biomark HD	Fluidigm	
Access Array	Fluidigm	
Seahorse XFe96 Analyzer	Agilent	
Qubit 2.0 Fluoremeter	ThermoFisher	
Agilent 2100 Bioanalyzer (Lab-on-a-Chip)	Agilent	
NovaSeq 6000	Illumina	

Table S3. Primersets, related to figure 2, 3 and 4

human_Gene_Exon_Fw	Sequence	Reverse	Sequence
hACTB_F4	AGCAAGCAGGAGTATGACGA	hACTB_R4	AGAAAGGGTGTAACGCAACTAA
hHMBS F1	GCAACGGCGGAAGAAAA	hHMBS R1	CGAGGCTTTCAATGTTGCC
hHPRT1 F4	ACACTGGCAAAACAATGCA	hHPRT1 R4	GGTCCTTTTCACCAGCAAG
hTMEM119_Qex2_Fw	CTGCTGATGTTCATCGTCTGT	hTMEM119_Qex2_Rev	TCACTCTGGTCCACGTACT
hP2RY12_Qex2_Fw	GGATACATTCAAACCCTCCAG	hP2RY12_Qex3_Rev	GAGGACCTGGGTGATTTTGTAG
hMERTK_Qex15_Fw	GGGACCTGCATACTTACTTACTT	hMERTK_Qex16_Rev	TCCATTCCCAGGGCAATATC
hTREM2_Qex1_Fw	CGGCTGCTCATCTTACTCTTT	hTREM2_Qex2_Rev	CAGTGCTTCATGGAGTCATAGG
hFTL_Qex1_Fw2	CAGCCTGGTCAATTTGTACCT	hFTL_Qex2_Rev2	GCCAATTCGCGGAAGAAGTG
hFTH1_Qex2_Fw	ATGAAGCTGCAGAACCAACG	hFTH1_Qex3_Rev	TGCACACTCCATTGCATTCAG
hSLC40A1_Qex7_Fw	TCTGTCAGTCTGCTGTTTGC	hSLC40A1_Qex8_Rev	TCTTGCAGCAACTGTGTCAC
hSLC11A2_Qex4_Fw	ATTTGCAGTCTGGAGCAGTG	hSLC11A2_Qex5_Rev	ACTGACGGTGACATACTTCAGC
hIL1B_Qex5_Fw_Edi	CATGGGATAACGAGGCTTATGT	hIL1B_Qex5_Rev_Edi	CATATGGACCAGACATCACCAA
hNLRP3_Qex4_Fw	TCATGCTGCCTGTTCTCATG	hNLRP3_Qex5_Rev	GCTGGTGCTCAGAACTGAAAAG
hCHI3L1_Qex6_Fw	ACAGCAGCTATGACATTGCC	hCHI3L1_Qex7_Rev	ATCTGTCAGGACTTGCATCCTC
hCD163_Qex6_Fw	ACTGCAAGAACTGGCAATGG	hCD163_Qex7_Rev	TTCAACACGTCCAGAACAGG
hTNF_Qex2_Fw_Edi	CCAGGGACCTCTCTCTAATCA	hTNF_Qex3/4_Rev_Edi	TCAGCTTGAGGGTTTGCTAC
hIL6_Qex3_Fw_Edi	CACTCACCTCTTCAGAACGAAT	hIL6_Qex4_Rev_Edi	GCTGCTTTCACACATGTTACTC
hRPL13a_Qex2_Fw	GCCATCGTGGCTAAACAGGTA	hRPL13a_Qex4_Rev	GTTGGTGTTCATCCGCTTGC
hHEXB_Qex2_Fw	GTCAGAGTGTGATGCTTTCCC	hHEXB_Qex3_Rev	TAAACCTCGTAATGCTCCCCA
hCD68_Qex4_Fw	TTGTCTACCTGAGCTACATGGC	hCD68_Qex5_Rev	TTGCTGCAACTGAAGCTCTG
hPYCARD_Qex3_Fw	TGGATGCTCTGTACGGGAAG	hPYCARD_Qex3_Rev	CCAGGCTGGTGTGAAACTGAA
hCASP1_Qex2_Fw	CACAGGCATGCCAAATTTGC	hCASP1_Qex3/4_Rev	GAGGAGCTGGAAAGGAAGAAAG
hAPOE_Qex3_Fw2	GGTCGCTTTTGGGATTACCTG	hAPOE_Qex4_Rev2	TCAACTCCTTCATGGTCTCGTC
hCD74_Qex4_Fw	TGACAGAGGACCATGTGATGC	hCD74_Qex5/6_Rev	ATCCAGCTCTCAAAGACCTTCC
hCTSB_Qex7_Fw	TACAGCCCGACCTACAAACAG	hCTSB_Qex8_Rev	AGCAGGAAGTCCGAATACACAG
hTYROBP_Qex4_Fw	ACTGAGACCGAGTCGCCTTAT	hTYROBP_Qex5_Rev	ATACGGCCTCTGTGTGTTGAG
hSPP1_Qex5_Fw	TGCCAGCAACCGAAGTTTTC	hSPP1_Qex6_Rev	TGATGTCCTCGTCTGTAGCATC
hSLC11A1_Qex2_Fw	TCCCCATCCCAGACACAAAAC	hSLC11A1_Qex3_Rev	AGCAATGCTCATGAGGAAGC
hHLA-DRA_Qex1_Fw	AGTCCCTGTGCTAGGATTTTTCA	hHLA-DRA_Qex2_Rev	ACATAAACTCGCCTGATTGGTC
hLPL_Qex2_Fw	TCATTCCCGGAGTAGCAGAGT	hLPL_Qex3_Rev	GGCCACAAGTTTTGGCACC
hCST7_Qex2_Fw	TCAACAACTGCACGAACGAC	hCST7_Qex3_Rev	GCAGGTGCTGGTTTTTCTTG
hCD33_Qex2_Fw	AAATCTCCCCAGCTCTCTGTG	hCD33_Qex3_Rev	AGCAGGTCAGGTTTTTGGAG
**Sanger sequencing**			
hAPOE_ex4_Fw5	AACAACTGACCCCGGTGGCG	hAPOE_ex4_Rev5	ATGGCGCTGAGGCCGCGCTC

Table S4. DEG result (separate file), related to Figure 5. All identified differentially expressed genes (DEGs) between treatment groups reported alongside their log foldchange (logFC), log counts per million (logCPM), F-statistic (F), *P*-Value and false discovery rate (FDR)

Table S5. GO analysis results (separate file), related to Figure 5. All identified significantly affected GO terms in the 4 different identified modules, on which Fig. 5F is based.

Table S6. GSEA analysis results (separate file), related to Figure 5. All overrepresented classes of genes identified using gene set enrichment analysis (GSEA), on which Fig. S3 is based.

Experimental procedures

Differentiation of iPSC-MG

IPSC-MG were differentiated following a protocol by Haenseler *et al* (Haenseler et al., 2017), with minor adaptations for upscaling of the production of iPSC-MG as described by Banerjee *et al* (Banerjee et al., 2020). iPSCs were cultured in mTESR (STEMCELL Technologies) until 80% confluent, after which they were lifted using Accutase (STEMCELL Technologies), counted and plated in an Aggrewell800 (STEMCELL Technologies) at a density of approximately 13.000 cells per miniwell to form embryoid bodies (EBs). Cells were cultured in mTESR supplemented with 50ng/mL VEGF (Peprotech), 50 ng/mL BMP4 (ThermoFisher), and 20 ng/mL SCF (ThermoFisher) for 5 days, after which EBs were lifted, collected over a strainer and plated in a 6-well plate at 12 EBs/well in 4mL X-VIVO 15 (Lonza) with 50ng/mL m-CSF (Peprotech) and 25 ng/mL IL-3 (Peprotech). Media was changed weekly and the development of myeloid precursors was tracked by phase contrast microscopy twice a week. After approximately 4 weeks, myeloid precursors were collected from the supernatant weekly, and plated on 0.01 µg/mL Poly-D-lysine (Sigma-Aldrich) and 0.01% gelatin (Sigma-Aldrich) coated plates at a density of 50.000/cm^2. Cells were cultured in microglia media, consisting of advanced DMEM/F12 (ThermoFisher) supplemented with Glutamax (ThermoFisher), N2-supplement (ThermoFisher), 2-mercaptoethanol (ThermoFisher), 100 ng/mL IL-34 (Peprotech), and 10 ng/mL GM-CSF (Peprotech). Half of the media was changed every other day and iPSC-MG were considered mature after 14 days.

Organoid generation and hiPSC-MG integration

Forebrain organoids were generated from control iPSC (iPSC4) using the STEMdiff Cerebral Organoid kit (StemCell Technologies; based on Lancaster et al. (Lancaster and Knoblich, 2014)), according to the manufacturer’s instructions. Briefly, iPSCs were collected as a single cell suspension using Accutase before being resuspended at 9000 cells (in 100 µL EB formation media) per well in a V-bottom 96 well plate, to form embryoid bodies (EBs; day 0). Media was topped up with additional 100 µL at day 2 and 4. On day 5, single EBs were transferred to a 48 well plate in induction media. On day 7, EBs were embedded in a drop (25-50 µL) of Matrigel and incubated in expansion media (12 EBs per well of a 6 well plate). At day 10, media was swapped for maturation media and organoids were cultured on an orbital shaker at 65rpm at 37, with media changed twice weekly for long-term culture. At day 43, organoids were transferred to a 24 well plate and $2x10^5$ hiPSC-MGs were collected and added to the well. Integration of hiPSC-MG into the organoids was tracked using brightfield imaging, over the next week.

Sanger sequencing

iPSCs were lifted using Accutase (STEMCELL technologies), spun down and a pellet was frozen for DNA isolation. DNA was isolated using the gDNA tissue miniprep system (Reliaprep) and purity of DNA was assessed using a ND-1000 Spectrophotometer (Nanodrop). PCR was performed using primers covering single-nucleotide polymorphisms rs429458 and rs7412, which determine the *APOE* genotype. Subsequently, the primer product was cleaned using the NucleoSpin PCR clean-up kit (Macherey-Nagel) before Sanger sequencing.

Immunofluorescence

For IF stainings, hiPSC-MGs were washed with PBS 24 hours post-intervention and fixed with 4% PFA for 10 min. Subsequently, cells were washed with PBS before permeabilization for 5 min with 0.1% Triton X-100 in PBS and subsequent washing with PBS. Organoids with integrated hiPSC-MG were washed with PBS, fixed in 4% PFA overnight at 4°C, washed with PBS and cryoprotected for 2-3 days in 30% sucrose at 4°C. Organoids were frozen in ethanol cooled with dry ice and stored at -80°C. Frozen organoids were cut on a cryostat (Leica CM3050) at 16 µm. Non-specific antigens were blocked for 1 hour with blocking solution (3% BSA in PBS). Cells were incubated overnight at 4°C with the following primary antibodies diluted in blocking solution: goat anti-Iba1 (1:500, Abcam, ab5076) with rabbit anti-TMEM119 (1:100, Abcam, ab185333), rabbit anti-P2RY12 (1:200, Sigma-Aldrich, HPA014518) or rabbit anti-MAP2 (1:1000, ThermoFisher, PA17646). Cells were washed in PBS and incubated with the secondary

antibodies donkey-anti-goat Alexa 488 (1:500, ThermoFisher, #A32814) and donkey-anti-rabbit (1:500, ThermoFisher, #A32754) in blocking solution for 3 hours at room temperature. Cells were washed with PBS and incubated for 5 min with 0.1 µg/mL DAPI (Sigma Aldrich) in PBS to label nuclei. Finally, cells on coverslips were mounted with ProLong diamond Antifade Mountant (ThermoFisher), while cells in 96-well plates were covered with PBS and imaged within 1 week. All PBS wash steps were performed three times for five minutes.

Imaging

Coverslips were imaged either using a BZ-X800 All-in-one fluorescence microscope (Keyence) with 10x objective for low magnification whole overview images (1460 x 1095 µm, at 960x720 pixels), or a TCS SP8 confocal microscope (Leica) with 40x objective for high-magnification images (385 x 385 µm, at 2048 x 2048 pixels) . All imaging conditions were identical for all experimental conditions for different stains (e.g. exposure time and laser power). HiPSC-MGs cultured and stained in 96-well flat bottom plates were imaged using the CellInsight CX7 High-Content Screening platform (ThermoFisher). Nuclei were identified using DAPI, and all identified DAPI+ nuclei were assessed for positivity of the cells for Iba1 (threshold determined based on negative control in which primary anti-Iba1 antibody was omitted).

Labile iron imaging

HiPSC-MG were cultured in 96-well flat bottom plates and following 24 h treatment with FC/IFNγ/AB hiPSC-MG were washed twice with PBS and incubated with 200uM FerroOrange (Goryo chemical) in HBSS for 30 min. Subsequently, hiPSC-MG were washed with PBS and Opti-MEM (Gibco) was added for live cell imaging BZ-X800 All-in-one fluorescence microscope (Keyence). Images were obtained with a 10x objective (1460 x 1095 µm, at 960x720 pixels). Labile iron quantity was quantified using Fiji (NIH), by firstly applying automated thresholding for intracellular iron using a iterative self-organzing data analysis (Isodata) algorithm (Ridler and Calvard, 1978), and subsequently measuring mean fluorescent intensity.

Western Blot

HiPSC-MGs cultured in a 12-well plate were washed with and frozen at -20 °C. After 24 hours, radioimmunoprecipitation assay buffer, supplemented with a protease inhibitor cocktail (Roche), was used lyse the cells. Subsequently, cells were sonicated using a Bioruptor Pico water bath sonicator (Diagenode) for 10 min. Protein concentrations were determined using Pierce BCA Protein Assay Kit (Thermo Fisher). 10 µg total protein was separated on a 4-20 % Mini-PROTEAN TGX Precast Protein Gel (BIO-RAD) under reducing conditions, and transferred to polyvinylidene difluoride (PVDF) membranes using a Trans-Blot Turbo Transfer system (BIO-RAD). PVDF membranes were blocked for 1 h with 5% milk in Tris buffered saline with 0.1% Tween 20 (hereafter TBST), and probed with rabbit anti-ferritin (1:1000, Abcam, ab75973) and mouse anti-β-actin (1:5000, Novus biologicals, NB600-501) overnight at 4 °C. Membranes were washed three times with TBST and incubated with goat anti-Rabbit IRDye 800CW (1:10000, LI-COR, 926-32211) and goat anti-Mouse IRDye 680CW (1:1000, LI-COR, 926-68070) and imaged using an Odyssey CLx infrared imaging system (LI-COR). Bands were quantified using Image Studio (Version 5.2, LI-COR), and normalized to β-actin signal.

Gene-expression analysis

RNA from hiPSC-MGs was collected using ReliaPrep RNA Miniprep system (Promega). Briefly, hiPSC-MGs cultured in a 12-well plate were washed with PBS and lysis buffer was added directly to the plate. Cells were pipetted repeatedly to shear the DNA and buffer was collected in an Eppendorf tube after which the protocol was followed as instructed by the manufacturer. Concentration and purity of RNA was assessed using a ND-1000 Spectrophotometer (Nanodrop).

Targeted gene expression analysis

200ng of RNA was converted into cDNA using the Transcriptor First Strand cDNA synthesis Kit (Roche). Gene expression was assessed using a 96x96 Dynamic Array (Fluidigm), which allows for testing of 96 samples and 32 primer sets in a single assay. A total list of primer sets used can be found in table S3. The Dynamic array was performed following manufacturer's guidelines. In brief, cDNA was pre-amplified for 11 cycles using a mixture of all primer sets, following protocol PN 100-5875 (Fluidigm). Subsequently, following protocol PN100-9792 (Fluidigm), a 96x96

dynamic array with integrated fluidic circuit was loaded with all 96 samples on one side and the 32 primersets (each 3 times) on the other side. Using an Access Array machine (Fluidigm), the cDNA samples and primers are mixed so that each well of the 96x96 contains one cDNA samples and 1 primerset (total of 9216 wells). The 96x96 dynamic array was then loaded in a Biomark HD (Fluidigm), which performs a real-time quantitative PCR reaction. Cq values were exported, and were normalized by substracting the Cq values of reference genes *ACTB* and *RPL13a* for highly expressed genes (low Cq values) or of reference genes *HMBS* and *HPRT1* for lowly expressed genes (high Cq values). Cq values were converted into relative expression counts and normalized to control treatment for each biological replicate.

RNAseq and analysis

For the generation of the full-length cDNA of the RNA-seq data, we used the method that was described as mcSCRB-seq (Bagnoli et al., 2018), with the following modifications to the workflow: instead of single cells, we used 100 ng isolated total RNA. Further, the PEG8000 was replaced with Polyvinylpyrrolidone (PVP) to reduce the viscosity of the reaction. We noticed that 0.5% PVP gave similar results as 7.5% PEG8000. The oligodT primer design was also different because it only contains the unique molecular index (UMI) with a PCR handle, which is partially Read 2 sequence primer from the Illumina platform (GAGTTCAGACGTGTGCTCTTCCGATCTNNNYRNNNYRNNNYRNNNTTTTTTTTTTTTTTTTTTTTTTTTTTTTTVN). The samples were uniquely barcoded using the PCR handle in the oligodT primer which completed the Read2 sequence primer, added the i7 Illumina barcode and the p7 sequence (CAAGCAGAAGACGGCATACGAGAT[i7_barcode]GTGACTGGAGTTCAGACGTGTGCTCTTCCGATCT). The i7 Illumina barcode served as the sample barcode. For the PCR reaction (9x cycle) we used the 2x KAPA HiFi HotStart ReadyMix (Roche) to generate the full-length cDNA libraries. After quantification of each sample using a Qubit 2.0 fluoremeter (ThermoFisher), the cDNA libraries were pooled together. The pooled cDNA libraries were subjected to an Illumina library prep. For this step, we used the KAPA HyperPlus kit (Roche) following the instruction of the manufacturer. Briefly, the pool of cDNA samples was fragmented for 5 min using the fragmentation enzyme from the kit followed by an End-Repair and A-tailing step. The fragmented cDNA was subjected to a double SPRI purification round (0.6x followed by 0.8x SPRI cleanup). The custom indexed adapter barcoded the pool of samples using the i5 Illumina barcode and introduced the p5 sequence to complete the Illumina library (top_adapter: AATGATACGGCGACCACCGAGATCTACAC[i5]ACACTCTTTCCCTACACGACGCTCTTCCGATC*T, bot_adapter GATCGGAAGAGCGT/3InvdT/). The ligated products were purified using 0.8x Ampure beads and the PCR with 2x Kapa ReadyMix (Roche) with the p5 and p7 primers was performed (9x cycle) to enrich for the correct molecules. After an additional double SPRI size selection, the Illumina library was quantified using a Qubit 2.0 fluoremeter (ThermoFisher) and checked on an Agilent 2100 Bioanalyzer (Lab-on-a-chip) for size distribution. Illumina paired-end sequencing of the library was done on a NovaSeq 6000 (Illumina) using manufacterer's instructions. After sequencing, Read 1 contained the cDNA information and read 2 only the UMI. The 3 prime RNA-seq data were analyzed using the RNA-seq module of nf-core (Version 3.2). First the data was mapped against the human genome (hg38) using STAR for alignment and Salmon for quantification, adopting default settings. For the pipeline to run effortlessly, the UMI in the read2 fastq file was added to the beginning of the same read id in the read1 fastq file. The modified Read1 fastq file then starts with the UMI and is followed by the cDNA sequence, which was used in the RNA-seq pipeline.

All subsequent analyses were conducted with the R/Bioconductor platform using R (Version 4.0.5). Raw countmatrix was converted in to counts per million (CPM) using the "cpm" function of edgeR (Version 3.32.1) (Robinson et al., 2010) and filtered for lowly expressed genes with (CPM > 10, for at least 4 samples). Hierarchical clustering was performed together with published data for iPSC-derived microglia (Abud et al., 2017; Kontinnen et al) using the hclust function with Euclidean distance on FPKM expression data for a subset of microglia genes published by Kontinnen et al. Filtered CPM counts were normalized using the trimmed mean of M values (TMM) approach using the calcNormFactors function of the edgeR package to normalize for batch effect. TMM uses raw data to estimate the appropriate scaling factors for different samples,

accounting for the different experimental conditions of the samples (Robinson and Oshlack, 2010). As only the final 200 basepairs of the 3' end were prepared in library prep, genecounts were not normalized for transcript length. Principal component analysis was performed on filtered normalized data with the prcomp function. Following, differential gene expression analysis was performed using the glmQLFTest function from edgeR. Differential expression was assessed for each gene using an exact text analogous to Fisher's exact test, but adapted for overdispersed data. We adjusted for any baseline differences between the different iPSC lines by fitting an additive model, to make comparison between treatments more precise, as was described in section 3.4.2 of the edgeR vignette (Chen et al.). Heatmaps were produced using the heatmap.2 function of gplots (Version 3.1.1) based on hierarchically clustered data of the top 60 differentially expressed genes (DEGs) for IFNγ treated samples and all DEGs for all other groups. Modules were extracted and functional enrichment analysis was performed using both Gene Ontology (GO) enrichment analysis and Gene Set Enrichment Analysis (GSEA) using the gost function of gprofiler2 (Version 0.2.0) and the GSEA function of the clusterProfiler (Version 3.18.1) package respectively. Results from RNAseq analysis were validated via comparison with results from targeted gene expression analysis using the Dynamic Array (Fluidigm). Although the log2 fold changes differ, the direction of log2 fold changes between different treatment groups is the same in both targeted gene expression analysis with a Dynamic Array and unbiased RNAseq analysis (Fig. S4).

Phagocytosis assay
Phagocytosis assay was performed using pHrodo Zymosan A particles (ThermoFisher) and imaged using an IncuCyte S3 Live-Cell Analysis System (Essen Bioscience). HiPSC-MGs were treated with previously defined conditions for 24 h, and additionally separate wells were treated with 100 ng/mL LPS (Sigma-Aldrich) for 24h or 10 Cytochalasin D (ThermoFisher) for 30 min. Media was replaced with Opti-MEM (Gibco) containing 25 µg/mL pHrodo particles and subsequently live-cell imaged every 30 min with a FITC filter (Exposure: 400 ms) and phase imaging for 24 h. Total fluorescence was quantified using Incucyte S3 software (Essen Bioscience), and normalized to cell confluence, which was quantified on the phase images using Incucyte software. Data was exported and area under the curve (AUC) and half time for max phagocytosis were calculated using Graphpad Prism (Version 8.00, La Jolla, San Diego, CA, USA).

Seahorse bioenergetics assay
Bio-energetic properties of the hiPSC-MGs were assessed using Seahorse XFe96 Analyzer (Agilent) for real-time cell metabolic analysis. HiPSC-MGs were cultured in 96-well Seahorse XF cell culture plate (Agilent) for 14 days as normal, before treatments as described. After treatment, both glycolysis and mitochondrial functional were assessed simultaneously, following a previously described protocol (Van den Bossche et al., 2015). In brief, treatment media was replaced with XF assay medium supplemented with 200 mM Glutamax (ThermoFisher) and cells were incubated in a 0% CO2 incubator for approximately 1h. Electron transport chain modulators were sequentially injected in a Seahorse XF96 analyzer: 25 mM glucose (to induce glycolysis); 1 µM oligomycin (to inhibit ATP synthase); 1 µM carbonyl cyanide-4-phenylhydrazone (FCCP; to uncouple mitochondrial respiration and assess maximal and spare respiratory capacity); 1 µM rotenone combined with 1 µM antimycin A (RA; to block mitochondrial complex I and III, and assess non-mitochondrial oxygen consumption; all Sigma-Aldrich). Both extracellular acidification rate (ECAR; mpH/min) and oxygen consumption rate (OCR; pMoles O_2/min) were assessed in real time, which were used to calculate glycolytic and mitochondrial respiration parameters, according to manufacturer's instructions. After the assay, cells were stained using 0.1 µg/mL DAPI (Sigma-Aldrich), and number of nuclei were counted using the CellInsight CX7 High-Content Screening platform (ThermoFisher), and used to normalized the results.

Replicates
Optimal reproducibility was attempted by studying all conditions simultaneously three times for each individual iPSC lines. Most assays have an n = 12, meaning 3 independent experiments were performed with 4 iPSC-lines. An experiment was considered independent when it was performed with a batch of iPSC-MG that was differentiated separately from precursor myeloid cells to microglia. A technical replicate was considered as another well from the same differentiation from myeloid cells to microglia. Technical replicates were used for the, labile iron imaging, phagocytosis assay and seahorse metabolism assay, and were averaged to obtain 1 datapoint.

References

Bagnoli, J.W., Ziegenhain, C., Janjic, A., Wange, L.E., Vieth, B., Parekh, S., Geuder, J., Hellmann, I., and Enard, W. (2018). Sensitive and powerful single-cell RNA sequencing using mcSCRB-seq. Nat. Commun. 2018 91 *9*, 1–8.

Banerjee, P., Paza, E., Perkins, E.M., James, O.G., Kenkhuis, B., Lloyd, A.F., Burr, K., Story, D., Yusuf, D., He, X., et al. (2020). Generation of pure monocultures of human microglia-like cells from induced pluripotent stem cells. Stem Cell Res. *49*.

Van den Bossche, J., Baardman, J., and de Winther, M.P.J. (2015). Metabolic characterization of polarized M1 and M2 bone marrow-derived macrophages using real-time extracellular flux analysis. J. Vis. Exp. *2015*, 53424.

Chen, Y., McCarthy, D., Ritchie, M., Robinson, M., Smyth, G., and Hall, E. edgeR: differential analysis of sequence read count data User's Guide.

Ewels, P., Hammarén, R., Peltzer, A., Moreno, D., Garcia, M., rfenouil, marchoeppner, Panneerselvam, S., F., S., jun-wan, et al. (2019). nf-core/rnaseq: nf-core/rnaseq version 1.4.2.

Haenseler, W., Sansom, S.N., Buchrieser, J., Newey, S.E., Moore, C.S., Nicholls, F.J., Chintawar, S., Schnell, C., Antel, J.P., Allen, N.D., et al. (2017). A Highly Efficient Human Pluripotent Stem Cell Microglia Model Displays a Neuronal-Co-culture-Specific Expression Profile and Inflammatory Response. Stem Cell Reports *8*, 1727–1742.

Huber, W., Carey, V.J., Gentleman, R., Anders, S., Carlson, M., Carvalho, B.S., Bravo, H.C., Davis, S., Gatto, L., Girke, T., et al. (2015). Orchestrating high-throughput genomic analysis with Bioconductor. Nat. Methods 2015 122 *12*, 115–121.

Lancaster, M.A., and Knoblich, J.A. (2014). Generation of cerebral organoids from human pluripotent stem cells. Nat. Protoc. 2014 910 *9*, 2329–2340.

Ridler, T.W., and Calvard, S. (1978). PICTURE THRESHOLDING USING AN ITERATIVE SLECTION METHOD. IEEE Trans. Syst. Man Cybern. *SMC-8*, 630–632.

Robinson, M.D., and Oshlack, A. (2010). A scaling normalization method for differential expression analysis of RNA-seq data. Genome Biol. 2010 113 *11*, 1–9.

Robinson, M.D., McCarthy, D.J., and Smyth, G.K. (2010). edgeR: a Bioconductor package for differential expression analysis of digital gene expression data. Bioinformatics *26*, 139–140.

Wickham, H., Averick, M., Bryan, J., Chang, W., McGowan, L.D., François, R., Grolemund, G., Hayes, A., Henry, L., Hester, J., et al. (2019). Welcome to the Tidyverse. J. Open Source Softw. *4*, 1686.

Yu, G., Wang, L.-G., Han, Y., and He, Q.-Y. (2012). clusterProfiler: an R Package for Comparing Biological Themes Among Gene Clusters. Https://Home.Liebertpub.Com/Omi *16*, 284–287.
