# Supplementary material for: Iron accumulation induces oxidative stress, while depressing inflammatory polarization in human iPSC-derived microglia
Source: Stem Cell Reports. 2022 May 5;17(6):1351–65. doi: 10.1016/j.stemcr.2022.04.006 (PMC9213827; doi:10.1016/j.stemcr.2022.04.006)
Supplement: Document S2. Article plus supplemental information [file mmc5.pdf]

Stem Cell Reports
Article
ISSCR
OPEN ACCESS

Iron accumulation induces oxidative stress, while depressing inflammatory polarization in human iPSC-derived microglia

Boyd Kenkhuis,[1,2,3,*] Michelle van Eekeren,[1] David A. Parfitt,[1] Yavuz Ariyurek,[4] Poulomi Banerjee,[2,3] Josef Priller,[2,3,5,6] Louise van der Weerd,[1,7] and Willeke M.C. van Roon-Mom[1]
[1]Department of Human Genetics, Leiden University Medical Center, Postzone S4-0P, P.O. Box 9600, 2300RC Leiden, the Netherlands
[2]Centre for Clinical Brain Sciences, University of Edinburgh, Edinburgh, UK
[3]UK Dementia Research Institute at University of Edinburgh, Edinburgh, UK
[4]Leiden Genome Technology Center, Leiden University Medical Center, Leiden, the Netherlands
[5]Department of Psychiatry and Psychotherapy, School of Medicine, Technical University of Munich, Munich, Germany
[6]Neuropsychiatry and Laboratory of Molecular Psychiatry, Charité, Universitätsmedizin Berlin, BIH and DZNE, Berlin, Germany
[7]Department of Radiology, Leiden University Medical Center, Leiden, the Netherlands
*Correspondence: b.kenkhuis@lumc.nl
https://doi.org/10.1016/j.stemcr.2022.04.006

SUMMARY

Iron accumulation in microglia has been observed in Alzheimer's disease and other neurodegenerative disorders and is thought to contribute to disease progression through various mechanisms, including neuroinflammation. To study this interaction, we treated human induced pluripotent stem cell-derived microglia (iPSC-MG) with iron, in combination with inflammatory stimuli such as interferon gamma (IFN-γ) and amyloid β. Both IFN-γ and iron treatment increased labile iron levels, but only iron treatment led to a consistent increase of ferritin levels, reflecting long-term iron storage. Therefore, in iPSC-MG, ferritin appeared to be regulated by iron revels rather than inflammation. Further investigation showed that while IFN-γ induced pro-inflammatory activation, iron treatment dampened both classic pro- and anti-inflammatory activation on a transcriptomic level. Notably, iron-loaded microglia showed strong upregulation of cellular stress response pathways, the *NRF2* pathway, and other oxidative stress pathways. Functionally, iPSC-MG exhibited altered phagocytosis and impaired mitochondrial metabolism following iron treatment. Collectively, these data suggest that in MG, in contrast to current hypotheses, iron treatment does not result in pro-inflammatory activation, but rather dampens it and induces oxidative stress.

INTRODUCTION

Iron accumulation is a feature of many different neurodegenerative disorders (NDs) including Alzheimer's disease (AD), Parkinson's disease (PD), Huntington's disease (HD), and multiple sclerosis (MS) (Bulk et al., 2020; Damulina et al., 2020; Popescu et al., 2017; Wang et al., 2016; Ward et al., 2014). Although iron is an essential element for processes such as myelination, neurotransmitter synthesis, and oxidative metabolism (Rouault, 2013), excessive iron is hypothesized to partake in Fenton's reaction, resulting in an accumulation of toxic reactive oxygen species (ROS) (Smith et al., 1997). Changes in both iron levels and distribution are specifically identified in disease-affected areas of the brain, and excess iron is found to accumulate both extra- and intracellularly. One cell type that has been shown to accumulate iron across different neurological conditions is microglia (Bagnato et al., 2011; Kenkhuis et al., 2021).

Microglia are the resident innate immune cells of the brain and are known to be essential for both brain development and brain plasticity (Paolicelli et al., 2011). As macrophages of the brain, they are also the first responders to changes in brain homeostasis (Prinz et al., 2019). In recent years, microglia have increasingly been implicated in NDs (Salter and Stevens, 2017), primarily in AD. Genome-wide association studies (GWAS) identified the majority of AD risk loci to be primarily or even exclusively expressed by microglia (Efthymiou and Goate, 2017). With regard to iron, microglia are considered the primary cells to sequester excessive amounts of iron in response to an acute insult such as observed in MS lesions (Bagnato et al., 2011). In addition, microglial iron accumulation was also identified in dystrophic microglia surrounding the pathological amyloid β (Aβ) plaques in AD, which was also accompanied by increased expression of the iron-storage protein ferritin (Kenkhuis et al., 2021). Ferritin-positive microglia have been observed repeatedly not only in AD but also in HD and PD (Jellinger et al., 1990; Lopes et al., 2008; Simmons et al., 2007). Moreover, signatures of altered iron metabolism were identified in disease-associated microglia (DAM) in APP/PS1 mice (Keren-Shaul et al., 2017), in human post-mortem AD tissue (Mathys et al., 2019), and in MG from normal-appearing gray matter in MS (van der Poel et al., 2019).

Although there is ample evidence that disrupted microglial iron homeostasis could contribute to disease, there are still several remaining questions. First, as both iron accumulation and neuroinflammation are observed concurrently in disease, it is still unclear whether the iron

© 2022 The Authors.
This is an open access article under the CC BY license (http://creativecommons.org/licenses/by/4.0/).

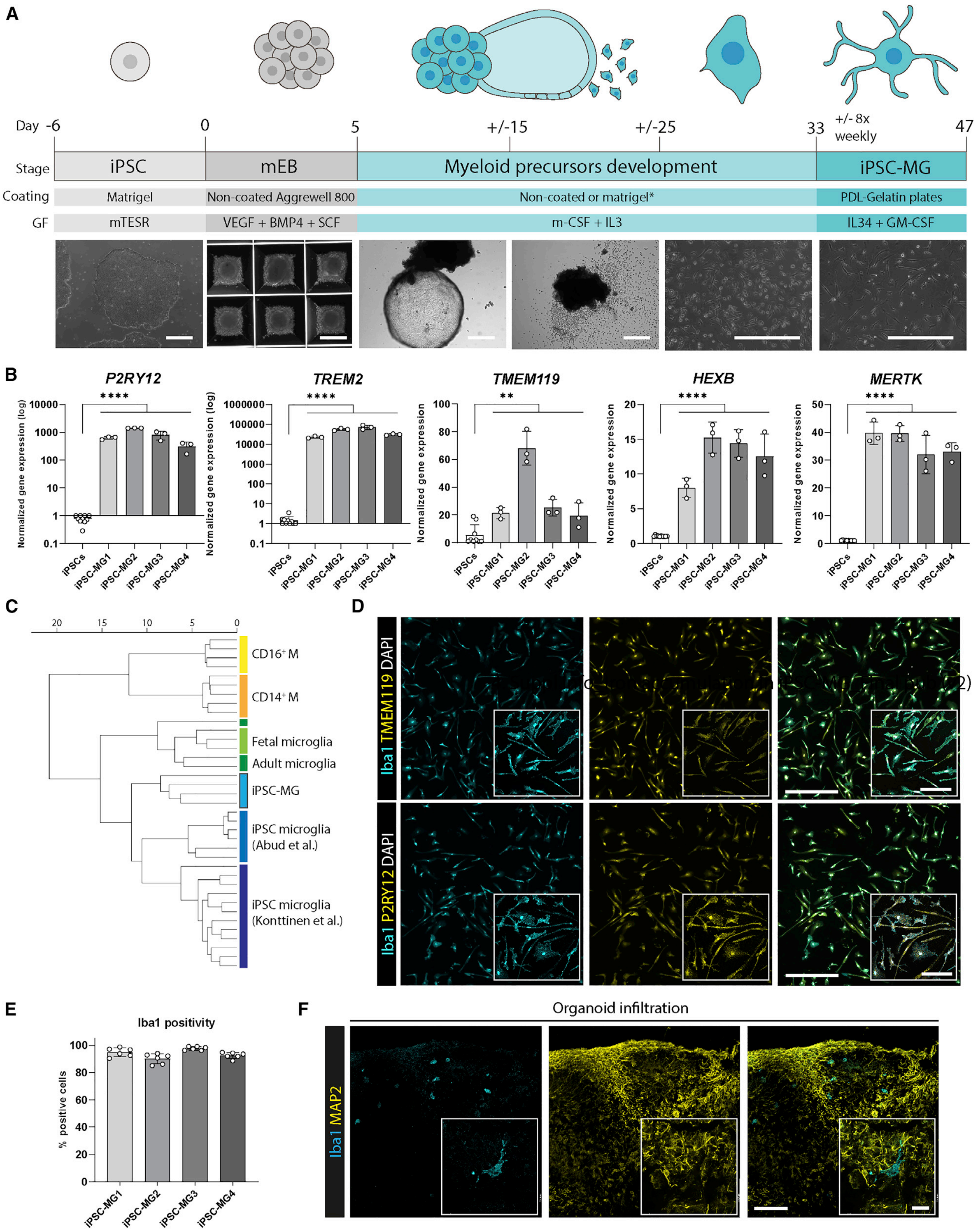

(legend on next page)

accumulation and ferritin expression observed in microglia in different NDs are a consequence of increased iron concentrations and/or iron depositions, a result of inflammatory activation, or a combination of both. Evidence from murine microglia and macrophages does suggest that pro-inflammatory activation promotes labile iron uptake both *in vitro* and *in vivo*, but it does not induce protein expression of light-chain ferritin, which is responsible for long-term iron storage (Holland et al., 2018; McCarthy et al., 2018). Second, it is unknown how iron affects microglial activation and function. Iron was found to be capable of potentiating pro-inflammatory interleukin (IL)-1β secretion induced by Aβ via nuclear factor (NF)-κB pathway activation in murine microglia (Nnah et al., 2020), and non-ferritin stored labile iron induced IL-1β production in peripheral mononuclear cells (Nakamura et al., 2016). Conversely, others found that iron decreases polarization toward M1 macrophages and inhibits the pro-inflammatory response (Agoro et al., 2018; Gan et al., 2017). Nonetheless, all of these data were obtained for murine microglia or even murine macrophages, and further investigation using human-derived microglia-like cells is warranted, considering there are significant differences between human and murine microglia and macrophages (Smith and Dragunow, 2014).

In this study, we used human induced pluripotent stem cell-derived microglia (iPSC-MG) to examine the effect of increased iron levels on Mmicroglia under normal and inflammatory conditions. Although iPSC-MG do not fully recapitulate the complete transcriptomic, morphological, and functional profile of microglia in the human brain, they are considered suitable to study the mechanisms involved in the processing of external stimuli. We treated iPSC-MG with the iron compound ferric citrate (FC) with or without the pro-inflammatory type II class cytokine interferon gamma (IFN-γ) or Aβ. Increasing concentrations of iron led to consistent increases in intracellular labile iron and ferritin expression. Treatment with IFN-γ only increased intracellular labile iron but not ferritin expression, while Aβ treatment had no effect on either ferritin expression or intracellular labile iron concentrations. On a transcriptomic level, iron-treated microglia showed disparate activation from the IFN-γ-induced pro-inflammatory pattern; iron even inhibited the activation of the NF-κB pathway, instead inducing cellular detoxification and oxidative stress. In addition, functional changes in both phagocytosis and mitochondrial metabolism were observed in response to iron treatment. Our study shows that high iron levels induce iron sequestration and ferritin storage in human iPSC-MG, which causes oxidative stress without polarization toward classic pro- or anti-inflammatory activation.

RESULTS

Generation and characterization of hiPSC-derived MG-like cells

We adapted a protocol by Haenseler et al. (2017) to differentiate human iPSCs into iPSC-MG. The generation of embryoid bodies (EBs) from iPSCs and successful patterning toward mesodermal lineage is shown in Figure 1A. Subsequently, mesodermal EBs (mEBs) were plated in media containing macrophage colony-stimulating factor (m-CSF) and IL-3 to induce the development of erythroid myeloid precursors. In the final step, myeloid precursors were replated and matured into iPSC-MG with the CSF1R ligand IL-34 and granulocyte macrophage (GM)-CSF. Morphological changes were assessed for each stage, and the finally generated iPSC-MG were assessed for expression of core MG markers on both gene and protein levels (Figures 1B–1E). Because of the importance of the *APOE* genotype for microglial function (Lin et al., 2018), iPSC-lines were genotyped for *APOE*, and two lines with the *APOE3/3* genotype and two with the *APOE3/4* genotype were selected (Figure S1A; Table S1). All of the results were compared for the *APOE* allele, but for none of the assays described in the following sections differences were

Figure 1. Generation and characterization of human iPSC-derived microglia (iPSC-MG)

(A) Schematic representation of the differentiation protocol (supplemental experimental procedures). Scale bar, 500 μm. *Coating dependent on iPSC-line.

(B) iPSC-MG showed significant upregulation of microglia signature genes P2RY12, *TREM2*, *TMEM119*, *HEXB*, and *MERTK* via qPCR (n = 12, 3 independent experiments for 4 iPSC lines; Student's independent t test).

(C) Hierarchical clustering of RNA-seq data shows iPSC-MG to cluster with other published iPSC-derived microglia and distinct from $CD14^+$ and $CD16^+$ monocytes (M).

(D) Using immunofluorescent staining, the majority of cells were positive for Iba1, P2RY12, and TMEM119. Scale bar, 400 μm; inset scale bar, 100 μm.

(E) Quantification of (D).

(F) Functionally, iPSC-MG could infiltrate and integrate in human brain organoids (representative images, n = 3, 1–2 independent experiments for 2 iPSC lines). Scale bar, 100 μm; inset scale bar, 20 μm. All of the bar graphs indicate means ± SDs. *p < 0.05, **p < 0.01, ***p < 0.001, ****p < 0.0001.

observed depending on the *APOE* allele. Using quantitative real-time PCR (qPCR), iPSC-MG showed a significant upregulation of between 1,000- and 10,000-fold for the core MG signature genes P2RY12 and *TREM2*, and a 10- to 60-fold increase in *TMEM119, HEXB, and MERTK* mRNA expression (Butovsky et al., 2014) (Figure 1B). Furthermore, comparison of RNA sequencing (RNA-seq) gene expression profiles using microglia and macrophage genes with the published datasets GSE89189 (Abud et al., 2017) and GSE135707 (Konttinen et al., 2019) revealed that our iPSC-MG hierarchically cluster together with other generated iPSC-MG, but are distinct from blood-derived $CD14^{+}$ and $CD16^{+}$ monocytes (Figure 1C). Using immunofluorescence, iPSC-MG showed clear expression of TMEM119, P2Y12, and Iba1 (Figure 1D). Quantification showed that 85%–99% of cells were positive for Iba1 in each of the 4 iPSC lines (Figure 1E). Finally, we tested the engraftment capacity of our generated iPSC-MG into human brain organoids, as has been demonstrated previously (Abud et al., 2017; Konttinen et al., 2019). We found that iPSC-MG infiltrated the organoids within 24 h after addition to the media, and remained viable close to microtubule-associated protein 2 $(MAP2)^{+}$ neurons (Figure 1F).

Iron loading in iPSC-MG occurs in response to increased iron concentrations but not inflammatory stimuli

Following the characterization of our iPSC-MG model, we wanted to assess whether iron accumulation and increased ferritin expression are a consequence of exposure to an increased concentration of iron, a result of inflammatory activation, or a combination of both. In murine microglia, ferritin expression was found to increase following inflammatory stimulation with IFN-γ and/or Aβ (McIntosh et al., 2019). Therefore, we treated our iPSC-MG with an increasing concentration of iron, the inflammatory stimulus IFN-γ, Aβ (1–42), or a combination of iron with IFN-γ or Aβ. Both ferric ammonium citrate (FAC) and FC induced considerable ferritin upregulation (Figure S1B), but we chose FC for further experiments, considering that citrate is also a physiological chelator of ferrous iron (Fe^{2+}) in the human brain (Ward et al., 2014). There are multiple genes and proteins involved, a schematic representation of which can be found in Figure 2A. We assessed levels of the iron storage protein ferritin and found a significant increase solely following stimulation with FC and independent of inflammatory activation with IFN-γ or Aβ (Figures 2B and 2C). In addition, using an iron-specific fluorescent tag that assesses non-ferritin bound labile iron, we found both FC and IFN-γ treatment to increase the uptake of iron into the cytosol of iPSC-MG (Figures 2D and 2E). Finally, we assessed the response of the different iron-metabolism genes, responsible for the maintenance of homeostatic iron concentrations. *FTL* mRNA expression was in line with the already observed increase in ferritin protein expression following FC treatment (Figure 2F). We were surprised to find that *FTH1* mRNA expression was only upregulated after FC treatment, but not in combination with IFN-γ or Aβ (Figure 2G). Gene expression of *SLC11A2* (import) was downregulated following FC treatment, likely to prevent further uptake of iron, but slightly upregulated after IFN-γ exposure (Figure 2H). Expression of the transferrin receptor, the alternative importer to *SLC11A2*, could not be detected in our iPSC-MG. Finally, the gene expression of *SLC40A1* (export) was increased only after a combined treatment with FC and IFN-γ, although expression levels varied greatly (Figure 2I). These results show that both FC and the inflammatory stimulus IFN-γ induce an influx of iron into the cytosol of iPSC-MG. However, only FC induces ferritin expression and long-term iron storage.

Iron loading induces iPSC-MG activation

Following the characterization of the iron-loading properties of iPSC-MG, we assessed the activation patterns of these cells. We assessed the morphological appearance of iPSC-MG by phase images and after immunohistochemical staining with the actin-cross-linking protein Iba1. IPSC-MG under control conditions showed small cell bodies with long, irregular cell processes (Figure 3A, arrowheads). After FC treatment, the long cell processes were partially retracted and the soma size increased (arrows), although not as much as after IFN-γ stimulation (asterisks). In addition to the increased soma size, IFN-γ stimulation induced a star-like morphology of cells with many shorter processes (asterisks and arrows). Exposure to Aβ induced few morphological alterations compared to control treatment (arrowheads). Next, we assessed the expression of core signature microglia genes, found to be downregulated following microglial activation in AD (Krasemann et al., 2017). Following IFN-γ exposure, all of the genes were found to be downregulated, except for *P2RY12,* which was upregulated both on qPCR (Figure 3B) and immunofluorescence (Figure S1F). Compared to IFN-γ, the gene expression signature was not as strong after FC treatment. FC induced no effect on *TMEM119*, but induced a significant downregulation of the homeostatic gene P2RY12 in all FC^{+} conditions (Figures 3C–3E). In line with the morphological evaluation, Aβ did not induce any significant transcriptomic changes in iPSC-MG (Figure 3B). All in all, iron loading induced the activation of iPSC-MG on both morphological and transcriptomic levels, although disparate from activation following IFN-γ treatment.

Iron loading depresses both pro- and anti-inflammatory activation patterns in iPSC-MG

In murine models, there have been conflicting reports of iron either inducing pro-inflammatory activation via

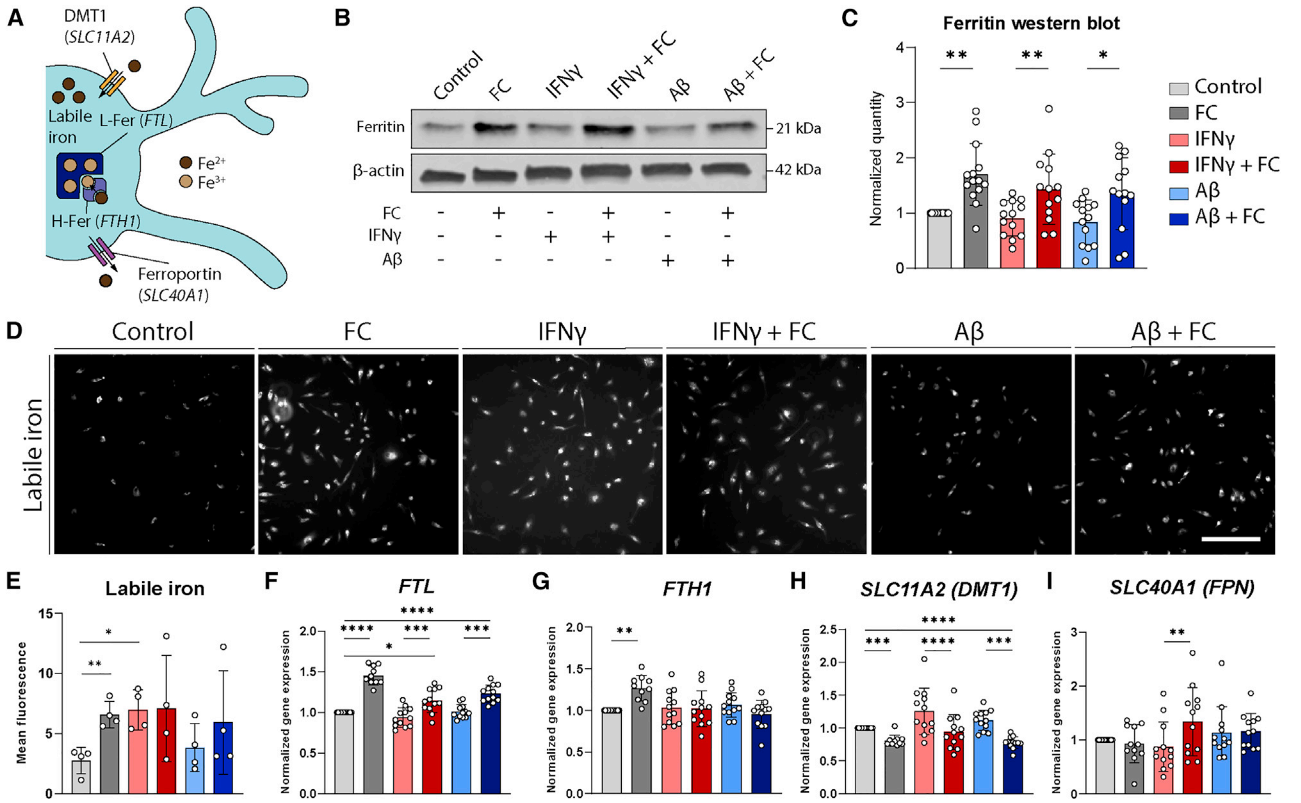

Figure 2. Increased iron concentrations, but not inflammatory activation, lead to microglial iron loading

(A) Schematic representation of iron metabolism in MG. Ferrous iron (Fe^{2+}) can be taken up by the cell via DMT1 (encoded by *SLC11A2*) and enter the labile iron pool. From here, ferrous iron can be oxidized into ferric iron (Fe^{3+}) via ferritin heavy chains (H-Fer, encoded by *FTH1*) and stored in ferritin light chains (L-Fer, encoded by *FTL*). Iron can be transported out of the MG via ferroportin (FPN, encoded by *SLC40A1*).

(B) Treatment of human iPSC-MG with 6 different conditions: control, ferric citrate (FC), interferon gamma (IFN-γ), IFN-γ + FC, amyloid β (Aβ), and Aβ + FC. Only FC treatment induces the increased expression of ferritin on western blot.

(C) Quantification of (B).

(D) Representative images of live cell imaging of labile iron showing increased cytosolic iron after exposure to FC, IFN-γ, or IFN-γ + FC.

(E) Quantification of fluorescent intensity in (D) (n = 4, 2 independent experiments for 2 iPSC lines).

(F–I) qPCR analysis of iron-metabolism genes shows upregulation of genes *FTL* and *FTH1,* responsible for iron storage; downregulation of *SLC11A2*, coding the iron-importer DMT1; and no difference in *SLC40A1*, coding the iron-exporter FPN, following FC exposure. Neither IFN-γ nor Aβ induces significant changes in iron-metabolism genes.

(B) and (F–I) n = 12–14 per condition, 2–4 independent experiments for 4 iPSC lines. Statistical analysis was performed using a mixed-effects model with Geisser-Greenhouse correction and Sidak post hoc correction. Scale bar, 200 μm. All of the bar graphs indicate means ± SDs. *$p < 0.05$, **$p < 0.01$, ***$p < 0.001$, ****$p < 0.0001$.

the NF-κB pathway (Nnah et al., 2020) or inhibiting the pro-inflammatory response (Agoro et al., 2018; Gan et al., 2017). Therefore, using targeted gene expression profiling, we examined genes associated with the classic paradigm of pro-inflammatory (M1) and anti-inflammatory (M2) MG and the NF-κB pathway, with downstream NLR family pyrin domain containing 3 (NLRP3) inflammasome activation. As expected, IFN-γ induced a strong pro-inflammatory transcriptional signature, with increased gene expression of M1 markers *TNF, IL6, and HLA-DRA* and decreased *CD163* mRNA expression (Figures 4A and 4B). Conversely, iron treatment depressed both pro- and anti-inflammatory profiles, with significant downregulation of *IL6*, *CD163*, and *CHI3L1* mRNA expression (Figures 4A and 4B). This effect was also observed when FC was added in combination with IFN-γ or Aβ. Similarly, individual genes of the NF-κB pathway (*NLRP3*, *IL1B*, *CASP1*, PYCARD) were upregulated following IFN-γ, but decreased after FC exposure (Figures 4C and 4D). Since there was evidence of iron dyshomeostasis in the microglial DAM signature and the iron-metabolism genes *FTL* and *FTH1* have been

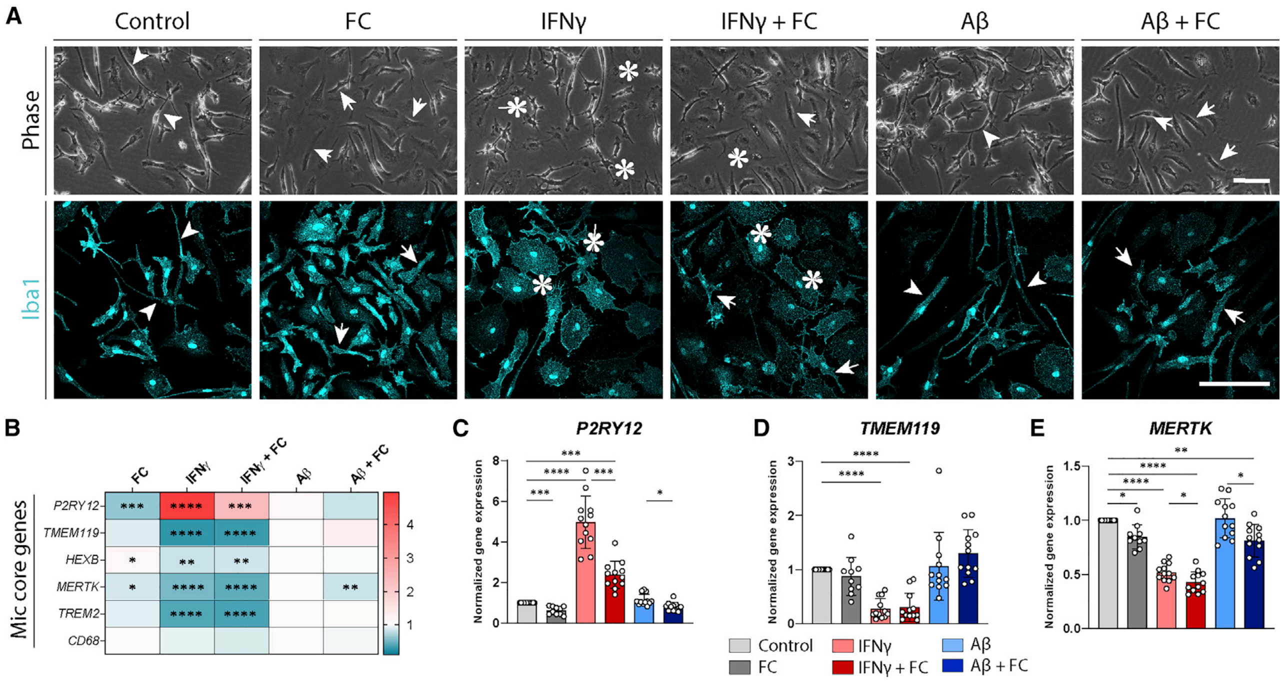

Figure 3. Morphological and transcriptomic activation following iron loading

(A) Representative phase and Iba1 immunohistochemical images of human iPSC-derived microglia, showing activation following FC and IFN-γ treatment (total phase images assessed n = 32 per condition, 8 independent differentiations from 4 lines).

(B–E) qPCR evaluation of microglia core genes. After IFN-γ treatment, P2RY12 showed strong upregulation, while *TMEM119* and *MERTK* showed strong downregulation. FC induced downregulation of P2RY12 and *MERTK*, albeit less than IFN-γ (heatmap, fold change normalized to control). n = 12 per condition, 3 independent experiments for 4 iPSC lines. Statistical analysis was performed using a mixed-effects model with Geisser-Greenhouse correction for matched data and Sidak post hoc correction. Scale bars, 100 μm. All of the bar graphs indicate means ± SDs. $^{*}p < 0.05$, $^{**}p < 0.01$, $^{***}p < 0.001$, $^{****}p < 0.0001$.

considered as core genes in DAM MG (Keren-Shaul et al., 2017; Mathys et al., 2019), we explored whether FC treatment would affect DAM signature genes in iPSC-MG. We selected 8 upregulated genes (*CD74*, *CTSB*, *TYROBP*, *APOE*, *SPP1*, *SLC11A1*, *LPL*, *CST7*) and 3 downregulated genes (*CD33* and previously shown P2RY12 and *TMEM119*) of this DAM signature. None of our different treatments resulted in consistent gene expression profiles resembling DAM (Figure 4E). Interestingly, exclusively FC induced expression of the *CTSB* and *TYROBP* genes (Figures 4F and 4G), both relevant in AD. While some overlap with changes in the expression of DAM genes was present (*CTSB*, *TREM2*, *CD33*, *P2RY12)*, iron treatment did not lead to the characteristic DAM signature. Bar graphs of all of the genes displayed in the heatmaps can be found in Figure S2.

Transcriptomic analysis shows oxidative stress in iron-loaded iPSC-MG

As FC did not induce classic pro-inflammatory activation as was previously published, but rather depressed pro- and anti-inflammatory activation, we performed whole-transcriptome RNA-seq, to elucidate which pathways were activated following iron loading. Principal-component analysis (PCA) of all of the samples showed that the first principal component (PC1) defined stimulation with IFN-γ (Figure 5A, PC1), whereas PCA2 showed the clustering of iPSC lines, underscoring the heterogeneity between the iPSC lines (Figure 5A, PC2). Correspondingly, differential gene expression (DGE) analysis showed the greatest number of differentially expressed genes (DEGs) between control and IFN-γ or IFN-γ + FC-treated iPSC-MG, followed by control versus FC and control versus Aβ + FC (Figure 5B). All of the groups that included FC treatment showed considerable overlap in the identified DEGs (Figures 5C and 5D), which underscored the reliability of the identified DEGs. The DEGs included *MEI*, which is involved in regulating metabolic oxidative activity, and several genes of the metallothionein family (*MT1A*, *MT1E*, *MT1L*, and *MT2A)*, encoding cysteine-rich proteins that bind divalent heavy metal ions. In addition, we performed DGE analysis on all FC^{-} versus FC^{+} groups (Figure 5B) and again found considerable overlap between the observed DEGs (Figure 5D). Some DEGs were only observed in the IFN-γ versus

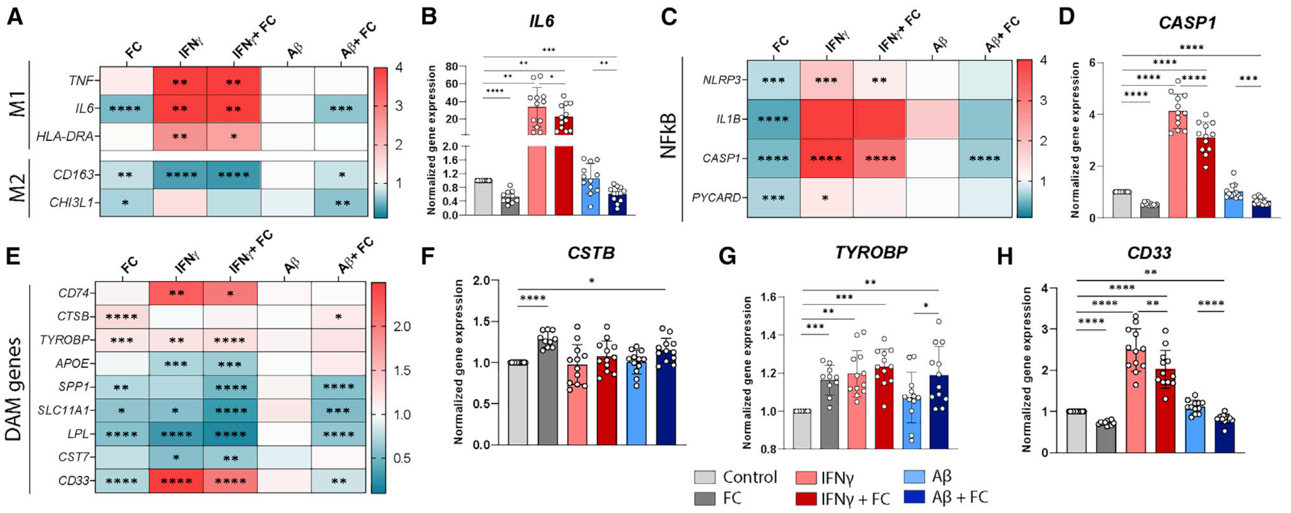

Figure 4. Iron treatment dampens pro- and anti-inflammatory activation

(A) Heatmap of changes in expression of classic M1 and M2 genes assessed with qPCR showed classic M1 genes upregulated by IFN-γ, but no change or downregulation of both M1 (*TNF*, *IL6* , and *HLA-DRA*) and M2 genes (*CD163* and *CHI3L1)* after FC treatment. Aβ showed no significant changes.

(B) Bar graph of *IL6*.

(C) FC treatment also leads to downregulation of genes involved in NF-κB pathway *NLRP3*, *IL1B*, *CASP1* and *PYCARD*, whereas IFN-γ leads to significant upregulation of this immune response-regulating pathway.

(D) Bar graph of *CASP1*.

(E) Both IFN-γ and FC exposure, or a combination of the two, leads to mixed results in up- and downregulation of core DAM genes.

(F–H) *CTSB* and *TYROBP* are significantly upregulated following FC exposure (F and G), whereas *SPP1*, *SLC11A1*, *LPL*, and *CD33* (H) are significantly downregulated. *APOE*, *CD74*, and *CST7* are affected by IFN-γ treatment.

n = 12 per condition, 3 independent experiments for 4 iPSC lines. Statistical analysis was performed using a mixed-effects model with Geisser-Greenhouse correction for matched data and Sidak post hoc correction. All of the bar graphs indicate means ± SDs. *$p < 0.05$, **$p < 0.01$, ***$p < 0.001$, ****$p < 0.0001$.

IFN-γ + FC groups, which may suggest a possible synergistic effect between proinflammatory activation and iron loading; a variety of genes involved in immune response (*A2M*, *C3*), chemokine regulation (*CXCL* family), and cell adhesion (*TGFBI*) were observed. Hierarchical clustering based on all DEGs from the iron-treated groups (N = 57–101) and the top 60 DEGs from IFN-γ-treated groups led to the identification of 4 modules, which appeared coordinately regulated across treatments (Figure 5E and Table S4 for all treatment-enriched DEGs). Hierarchical clustering using the previously identified DEGs showed FC-treated samples (FC and FC + Aβ) to be highly similar and to separate from IFN-γ and IFN-γ + FC- and control and Aβ-treated samples. Module 1 (Mod1; dark blue) consisted of genes that were downregulated upon iron treatment and included the previously mentioned metallothionein family. Gene Ontology (GO) enrichment analysis confirmed that processes involved in the binding of metals or other responses to metal ions were most significantly downregulated (Figure 5F). Moreover, in line with the targeted gene expression analysis, several processes relating to immune activation of myeloid cells also appeared significantly downregulated. Genes from the second module, Mod2, were upregulated in IFN-γ-treated samples (Figure 5E, pink) and corresponded to pro-inflammatory/M1-associated pathways such as defense and immune responses against foreign organisms and cytokine response processes (Figure 5F, pink). Also, upregulated processes of metal ion homeostasis were observed, which corresponded to previously observed findings of increased labile iron influx following IFN-γ treatment (Figure 2E). The third module, Mod3, contained genes upregulated in FC-, Aβ + FC-, and IFNγ + FC-treated samples (Figure 5F, yellow). GO enrichment analysis revealed upregulated processes of response to toxic substances, oxidative stress, and ROS (Figure 5E, yellow). Likewise, NRF2 was the most significantly upregulated pathway, which is activated under oxidative stress conditions and activates antioxidative genes and proteins. The upregulation of NRF2 and cellular stress response pathways, as well as the downregulation of metal ion responses, were also confirmed with independent gene set Enrichment analysis (GSEA) on the log fold change of all genes between treatments (Figure S3). The full list of significant pathways identified with GO and GSEA, can be found in

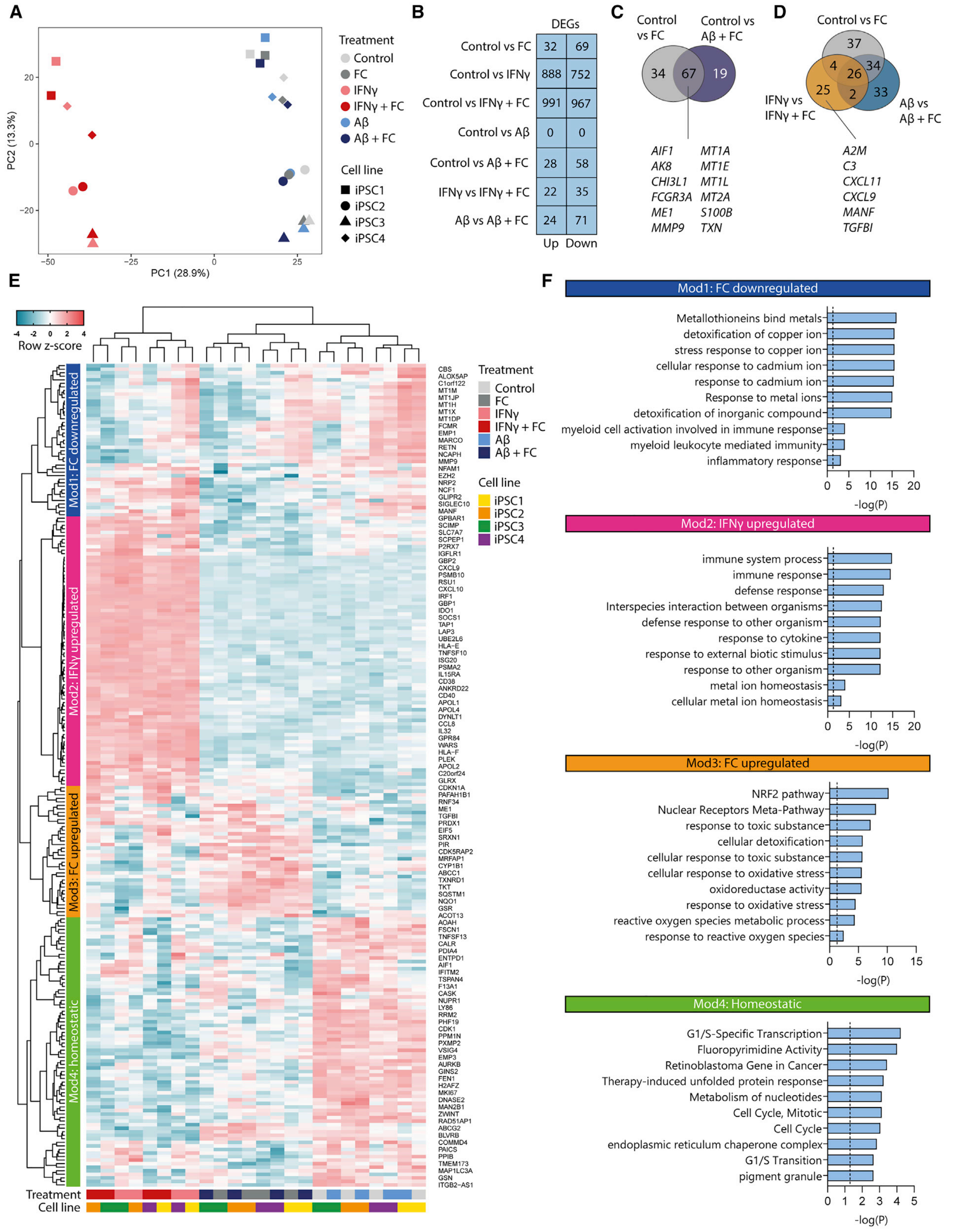

(legend on next page)

Tables S5 and S6, respectively. Finally, genes from the fourth module, Mod4, were downregulated in both IFN-γ- and FC-treated samples and corresponded to homeostatic processes such as cell-cycle regulation. Using whole-transcriptome RNA-seq, we could confirm that iPSC-MG treated with FC show a disparate activation pattern from the one induced by the pro-inflammatory cytokine IFN-γ. Moreover, we now identified pathways of cellular detoxification, oxidative stress, and downregulated homeostatic function in iron-loaded iPSC-MG.

Iron affects phagocytosis in iPSC-MG

In addition to transcriptomic and protein changes, we explored the effect of iron on phagocytosis, which is one of the key functions of innate immune cells such as microglia. After treatment with our six previously defined interventions and an additional positive and negative control (lipopolysaccharide [LPS] and cytochalasin D, respectively), we added pHrodo zymosan beads to the media and performed live cell imaging every 30 min for 24 h to assess the phagocytic speed and maximum capacity of the iPSC-MG (Figure 6A). IFN-γ greatly reduced the total phagocytic capacity of iPSC-MG, almost to the level of our negative control cytochalasin D (Figure 6B). FC, however, induced a slight increase in total phagocytic capacity, almost similar to that of LPS (Figure 6B). However, the initial speed of phagocytosis was reduced in all FC-treated groups (Figure 6B). We quantified the half-time by first performing a nonlinear regression curve fitting with an exponential one-phase association and subsequently calculating the time it takes for the iPSC-MG to reach half of the maximum phagocytic capacity based on this fitted curve (Figure 6D). This showed that FC treatment significantly increased phagocytosis half-time (Figure 6E).

Increased iron impairs metabolic activity of iPSC-MG

To assess the effect of iron loading on the bioenergetics of iPSC-MG, we measured both the oxygen consumption rate (OCR) and extracellular acidification rate (ECAR) to assess mitochondrial metabolism and glycolysis, respectively, after treatment with our six groups of compounds. The iPSC-MG showed the expected response to electron transport chain modulators and showed proper oxidative phosphorylation, as measured with OCR, but only very little glycolysis, as measured with ECAR (Figure 7A). The quantification of the OCR curves showed a significant increase in non-mitochondrial respiration, proton leak, and basal respiration following IFN-γ exposure (Figures 7B–6D). FC treatment led to reduced maximum respiration, spare respiratory capacity, and subsequent ATP production when comparing FC^+ versus FC^- conditions (Figures 7E–7G). This indicates that IFN-γ treatment induces a switch from oxidative respiration toward anaerobic glycolysis, often associated with an M1 pro-inflammatory phenotype, which is in line with transcriptomic data (Figures 4A and 5F). Iron appeared to influence electron transport chain function, resulting in lower maximum and spare respiratory capacity rates following treatment with the uncoupler carbonyl cyanide 4-(trifluoromethoxy) phenylhydrazone (FCCP). Although neither indicate cellular dysfunction per se, it has been suggested that both higher maximum and spare respiratory capacity exist so that cells can appropriately respond to increases in demand and withstand periods of stress (Divakaruni et al., 2014). Decreased levels would therefore indicate cellular stress and increased susceptibility to cell death.

DISCUSSION

In this study, we showed that exposure to an increasing concentration of iron results in ferritin iron loading in iPSC-MG, independent of inflammatory activation by IFN-γ or Aβ. Iron-loaded iPSC-MG showed both morphological and transcriptomic activation, but dampened both classic pro- or anti-inflammatory activation patterns. Instead, the NRF2 and other oxidative stress pathways were activated.

Figure 5. Transcriptomic analysis shows oxidative stress in iron-loaded iPSC-MG

(A) Principal-component analysis shows clustering of samples based on IFN-γ treatment (PC1) and iPSC line (PC2) (n = 4 per treatment, 1 independent experiment for 4 iPSC lines).

(B) Differentially expressed gene (DEGs) counts for each treatment. Statistical testing was performed using the Fisher's exact test, adapted for overdispersed data with additive fitted model to correct for iPSC line variance. p values were corrected for false discovery rate (FDR), and a statistical threshold of FDR < 0.05 was applied.

(C) Overlap of DEGs for FC- and Aβ + FC-treated cells.

(D) Overlap of DEGs for all FC^-- versus FC^+-treated groups (D). Genes mentioned in the Venn diagram are included based on interest. (E) Heatmap with *Z* scores for the (top 60) DEGs of all of the treated groups. Hierarchical clustering identified 4 modules: FC downregulated (dark blue), IFN-γ upregulated (pink), FC upregulated (yellow), and homeostatic (green).

(F) Gene Ontology enrichment analysis of the identified modules showed consistent immune response activation in IFN-γ-treated samples (Mod2; pink), but evidence of increased cellular and more specifically oxidative stress in FC-treated samples (Mod3; yellow). In addition, cellular response to heavy metals appeared downregulated in FC-treated samples (Mod1; dark blue), whereas homeostatic processes involving cell-cycle regulation were downregulated in all FC and IFN-γ-treated samples (Mod4; green). The dotted line indicates a significance threshold of $p < 0.05$.

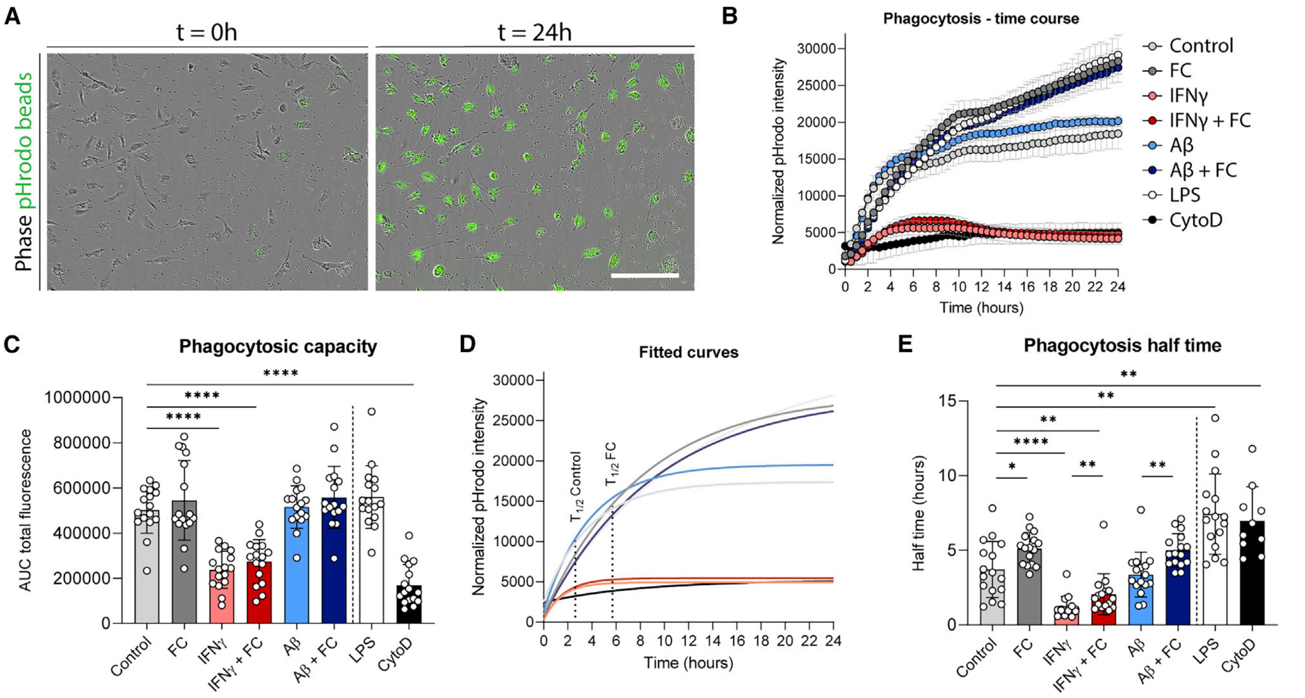

Figure 6. Slower phagocytosis following iron loading in iPSC-MG

(A) Representative images of phagocytosed pHrodo zymosan beads at initial seeding and after 24 h.

(B) Time course of phagocytosis across the 6 different conditions, and with a positive (LPS) and negative (cytochalasin D) control. Mean ± SD of 1 iPSC-MG line with 6 technical replicates.

(C) Significant reduction of total phagocytic capacity following IFN-γ exposure, and a small, non-significant increase was observed following FC or LPS exposure.

(D) Estimated slope of phagocytosis time course in (B), from which half-time, the time it takes to reach half their maximum phagocytic capacity, can be calculated. Shown for control and FC.

(E) Phagocytosis half-time is significantly increased in each condition with FC and decreased after IFN-γ. (C andE) n = 16 per condition, 3–5 independent experiments for 4 iPSC lines. Statistical analysis was performed using a mixed-effects model with Geisser-Greenhouse correction for matched data and Sidak post hoc correction. Scale bars, 200 μm. All of the bar graphs indicate means ± SDs. *$p < 0.05$, **$p < 0.01$, ***$p < 0.001$, ****$p < 0.0001$.

Functionally, the rate of phagocytosis was reduced, and iron treatment resulted in decreased maximum and spare respiratory capacity of mitochondrial metabolism.

We showed that treatment of iPSC-MG with FC resulted in significantly increased labile iron, as did treatment with IFN-γ, while Aβ had no effect. Conversely, only FC treatment and not IFN-γ treatment resulted in the increased expression of the iron-storage protein ferritin. These results are in line with previous results in murine immortalized microglia, in which treatment with FAC induced significant iron uptake and increased ferritin expression (McCarthy et al., 2018). However, contrary to our findings, they observed an increase in divalent metal transporter 1 (DMT1) and heavy-chain ferritin expression in this cell line in response to pro-inflammatory activation with LPS, which we did not observe in iPSC-MG following pro-inflammatory activation with IFN-γ stimulation. Correspondingly, light-chain ferritin levels remained unaltered after inflammatory activation. Therefore, although the expression of ferritin is often considered the result of pro-inflammatory activation, our results in iPSC-MG indicate that the ferritin response is predominantly mediated by iron rather than by inflammatory activation.

There have been conflicting results regarding the activation status of microglia following iron treatment. A separate study using a murine immortalized microglia cell line found activation of the NF-κB pathway with downstream pro-IL-1β production following treatment with iron and Aβ (Nnah et al., 2020). Similarly, FAC treatment induced IL-1β production in murine isolated peripheral monocytes (Nakamura et al., 2016). In contrast, other studies found that iron inhibited the polarization toward pro-inflammatory M1 macrophages induced by either LPS or IFN-γ, but instead induced the anti-inflammatory M2 phenotype (Agoro et al., 2018; Gan et al., 2017). Our study is the first to test the response to iron

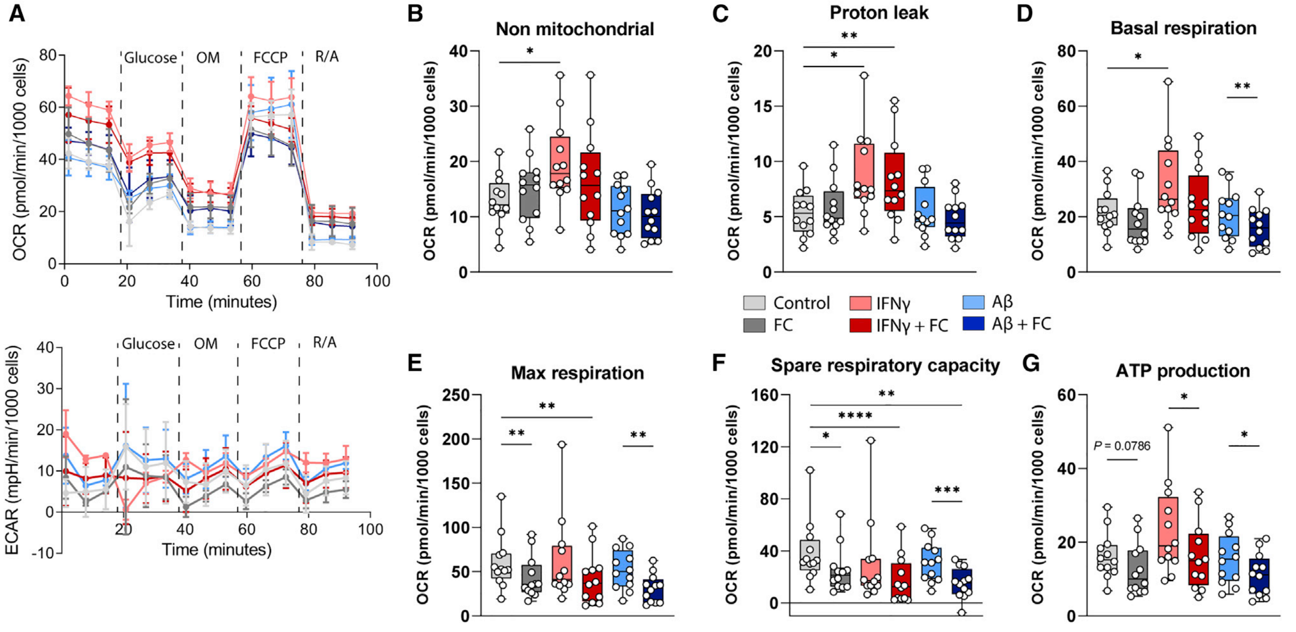

Figure 7. Iron loading affects metabolism of iPSC-MG

(A) Representative oxygen consumption rate (OCR; top) and extracellular acidification rate (ECAR; bottom) curves following treatments show proper oxidative respiration (OCR), but minimal anaerobic glycolysis (ECAR). Mean values ±SDs are plotted (n = 4 technical replicates).

(B–D) IFN-γ-treated iPSC-MG showed increased non-mitochondrial respiration, proton leak, and basal respiration, whereas FC treatment resulted in minimal changes.

(E–G) Treatment with FC resulted in decreased maximum respiration, spare respiratory capacity, and ATP production, for all FC^{+} vs FC^{-} groups.

(B–G) Statistical testing using repeated measures (RM) 1-way ANOVA with Geisser-Greenhouse correction was performed for matched data, and a post hoc Sidak correction was performed. n = 12 per condition, 2–4 independent experiments for 4 iPSC lines. Boxplots display median with interquartile ranges and maximum and minimum. *p < 0.05, **p < 0.01, ***p < 0.001, ****p < 0.0001.

using human iPSC-MG, and we found that iron treatment depresses both pro- and anti-inflammatory activation. Targeted gene expression analysis showed that treatment with FC (alone or in combination with IFN-γ) dampened the M1 phenotype, with downregulated NF-κB pathway genes *NLRP3*, *IL1B*, *CASP1*, and *PYCARD* and downregulated M1 genes tumor necrosis factor (TNF), *IL6*, and *HLA-DRA*. However, we also observed the downregulation of the M2 genes *CD163* and *CHI3L1*. RNA-seq analysis confirmed the downregulation of immune activation in iron-treated human iPSC-MG. All in all, the homeostatic function of iPSC-MG is clearly affected by iron treatment, but rather than classic pro- or anti-inflammatory activation, microglia appeared oxidatively stressed. In line with this, our bioenergetics analysis data did not indicate a switch toward glycolysis, as would be expected under pro-inflammatory conditions and observed after IFN-γ treatment, but rather suggested lower metabolic capacity, which is indicative of cellular stress. These findings are in agreement with a study by Yauger et al. (2019), in which they used immortalized rat instead of murine microglia, and showed increased ROS production following iron treatment, without an accompanying alteration in pro-inflammatory/M1 polarization markers. This study also highlights the discordance between species.

We also investigated the effect of Aβ, alone and in combination with iron, but found no direct effect of Aβ. To date, conflicting results exist on the effect of Aβ, which appears to depend on the model in which the results were obtained. Previous studies in murine MG showed the activation of NLRP3 and increased IL-1β production following treatment with oligomeric Aβ via Toll-like receptors (TLRs), which was exacerbated by iron (Burm et al., 2015; Nnah et al., 2020). Conversely, a recent study investigating TLR-meditated NLRP3 inflammasome activation in human iPSC-MG showed that contrary to other proteins, soluble oligomeric Aβ did not induce NLRP3 activation and subsequent IL-1β production (Trudler et al., 2021). Also in line with our results, another study using a human iPSC-MG model found almost no difference after treatment with oligomeric Aβ in either chemokinesis or phagocytic

function (Konttinen et al., 2019). Therefore, murine and human microglia may differ too much to allow for valid comparisons. However, considering that Aβ is a notoriously difficult protein to study due to its self-aggregating properties and function that heavily depends on its conformation (Ladiwala et al., 2012), the opposing findings could also be due to technical differences across studies. Moreover, as mentioned briefly in the Results section, no effect was seen of the *APOE* genotype, despite previous reports showing a clear effect of *APOE* genotype on the function of iPSC-MG (Konttinen et al., 2019; Lin et al., 2018). However, in our case, we were likely underpowered to detect an effect, as we used only 4 non-isogenic lines harboring the *APOE3/3* and *APOE3/4* genotypes, as opposed to the isogenic *APOE3/3* and *APOE4/4* pairs as used in the previous studies.

From clinical *in vivo* MRI and post-mortem liquid crystal polymer-mass spectrometry studies, it has been suggested that iron levels correlate with accelerated cognitive decline, not only in AD (Ayton et al., 2017, 2020, 2021) but also in PD and amyotrophic lateral sclerosis (ALS) (Devos et al., 2019; Thomas et al., 2020), which increases the likelihood of a pathology-independent common pathway being responsible for the observed clinical effect. A previous study showed that iron could induce a dose-dependent increase in ROS production via nicotinamide adenine dinucleotide phosphate (NADPH) oxidases, which affected neuronal survival in a co-culture (Yauger et al., 2019). Similarly, we also found the NADPH pathway to be among the most significantly upregulated pathways in the iron-treated iPSC-MG. In addition, iron accumulation may contribute to neurodegeneration via ferroptosis, the iron-dependent cell-death pathway (Dixon et al., 2012), which has been found to play an important role in MG (Kapralov et al., 2020).

Although homogeneous cultures of iPSC-MG are a powerful and suitable model to study the cell-intrinsic effect of single or different combinations of stimuli, there are also several limitations. First, iPSC resemble immature developing microglia rather than mature or even degenerating microglia as associated with NDs. Second, the conditions used in this study cannot reflect the complex mix of cues that MG receive under diseased conditions, and potential synergistic effects will be missed.

Although iron accumulation and microglia activation are considered hallmarks of disease of many NDs, and evidence of altered microglial iron metabolism is found in both immunohistochemistry (IHC) and transcriptomic studies, the direct effect of iron on human MG had not been previously studied. Here, we show that microglial iron metabolism alterations reflect iron levels rather than inflammatory activation. Moreover, treatment with iron resulted in transcriptomic activation with signs of cellular detoxification and oxidative stress, together with impaired metabolic metabolism and altered phagocytic function. Further investigation is required to dissect the effects of these changes on microglial function, the interaction with other neural cell types, and the potential impact on disease progression.

EXPERIMENTAL PROCEDURES

FC, IFN-γ, and Aβ treatment

FC (Sigma-Aldrich) was dissolved in sterile H_2O at 10 mM concentration 48 h in advance and put in a spinning rotor. Ascorbate (Sigma-Aldrich) was dissolved in sterile water at 500 mM concentration. A total of 100 μg hexafluoro-2-propanol (HFIP)-prepared Aβ (Bachem) was reconstituted in 10 mL pure DMSO, sonicated in a water bath for 10 min, further diluted in 90.8 μL phenol-red free HAM/F12, and incubated for 24 h at 4°C to obtain 200 μM oligomeric Aβ, as previously described (McCarthy et al., 2016). Ascorbate was added to media containing 50 μM FC to ascertain the reduction of the iron toward Fe^{2+}, which can be transported into the cell via DMT1 importers. FC concentration was chosen based on the literature and the clear iron-loading effect seen on western blot and labile iron imaging (Figures S1D and S1E). Similarly, the final media was depleted of N2-supplement, as N2 contains high concentrations of transferrin, the apo-transferrin of which can bind to the additional free iron. We tested whether the omission of the N2-supplement for the final 24 h resulted in transcriptomic activation of the iPSC-MG itself, which was not the case (Figure S1C). Aβ and IFN-γ (Peprotech) were added at 1 μM and 20 ng/mL, respectively, to media without N2-supplement, based on concentrations used in Nnah et al. (2020) and Holland et al. (2018), respectively. For all assays, all 6 treatments (control, FC, IFN-γ, IFN-γ + FC, Aβ, Aβ + FC) were performed in parallel via a complete change of media with the appropriate treatment group. iPSC-MG were treated for 24 h before subsequent assays were performed (i.e., lysate collection for WB, paraformaldehyde (PFA) fixation, RNA collection, labile iron imaging, phagocytosis assay, or seahorse metabolism assay). Further details on these assays can be found in the supplemental experimental procedures.

Statistics

All of the data were inspected for being gaussian distributed. If normally distributed, data plots represent the mean and the standard deviation, while not normally distributed data are shown using the median with corresponding interquartile range, which is stated in each figure legend. A comparison of two continuous variables was performed using a two-tailed unpaired Student's independent t test (normally distributed). A comparison of data from the 6 treatments, when normally distributed, was performed using a one-way ANOVA or mixed-effects model (in case of missing values) with Geisser-Greenhouse correction to adjust for the lack of sphericity, and followed up with a post hoc Sidak multiple comparisons test. Not normally distributed data were compared using a nonparametric Kruskal-Wallis test, followed with Dunn's multiple comparisons test. A significance level of $p < 0.05$ was used. All of

the statistical tests were performed using GraphPad Prism (version 8.00). For DGE of the RNA-seq data, we used the edgeR package (version 3.32.1) in R (version 4.0.5). Differential expression was assessed for each gene using an exact test analogous to Fisher's exact test, but adapted for overdispersed data. We adjusted for any baseline differences between the different iPSC lines by fitting an additive model to make the comparison between treatments more precise.

A detailed description of all of the experimental procedures and all of the reagents, software, and machines that were used can be found in the supplemental information and Table S2).

Data and code availability

The authors confirm that the data supporting the findings of this study are available within the article and/or its supplemental information. Raw RNA-seq data generated in this paper are available through EGAS00001006112. The R script used for the analysis of the RNA-seq data will be shared upon request. Reanalyzed datasets for this study are available through GSE135707 and GSE89189.

SUPPLEMENTAL INFORMATION

Supplemental information can be found online at https://doi.org/10.1016/j.stemcr.2022.04.006.

AUTHOR CONTRIBUTIONS

Conceptualization, B.K., L.v.d.W., and W.M.C.v.R.-M.; investigation, B.K., M.v.E., D.A.P., and Y.A.; methodology, B.K., D.A.P., and P.B.; data curation, B.K.; formal analysis, B.K.; funding acquisition, B.K., L.v.d.W., and W.M.C.v.R.-M.; supervision, J.P., L.v.d.W., and W.M.C.v.R.-M.; Writing – original draft, B.K., L.v.d.W., and W.M.C.v.R.-M.; Writing – review & editing, all of the authors.

CONFLICTS OF INTERESTS

The authors declare no competing interests. All of the co-authors have seen and agree with the contents of the manuscript.

ACKNOWLEDGMENTS

We thank L. van der Graaf for management of the iPSC laboratory. This work was funded by W.M.C.v.R.-M. B.K. is supported by an MD/PhD grant from the Leiden University Medical Center. In addition, he has received funding from an early career fellowship from Alzheimer Nederland (WE.15-2018-13) and a Eurolife Scholarship for Early Career Researchers.

Received: December 8, 2021
Revised: April 9, 2022
Accepted: April 11, 2022
Published: May 5, 2022

REFERENCES

Abud, E.M., Ramirez, R.N., Martinez, E.S., Healy, L.M., Nguyen, C.H.H., Newman, S.A., Yeromin, A.V., Scarfone, V.M., Marsh, S.E., Fimbres, C., et al. (2017). iPSC-derived human microglia-like cells to study neurological diseases. Neuron *94*, 278–293.e9. https://doi.org/10.1016/j.neuron.2017.03.042.

Agoro, R., Taleb, M., Quesniaux, V.F.J., and Mura, C. (2018). Cell iron status influences macrophage polarization. PLoS One *13*, e0196921. https://doi.org/10.1371/journal.pone.0196921.

Ayton, S., Fazlollahi, A., Bourgeat, P., Raniga, P., Ng, A., Lim, Y.Y., Diouf, I., Farquharson, S., Fripp, J., Ames, D., et al.; the Australian Imaging Biomarkers and Lifestyle AIBL Research Group (2017). Cerebral quantitative susceptibility mapping predicts amyloid-β-related cognitive decline. Brain *140*, 2112–2119. https://doi.org/10.1093/brain/awx137.

Ayton, S., Wang, Y., Diouf, I., Schneider, J.A., Brockman, J., Morris, M.C., and Bush, A.I. (2020). Brain iron is associated with accelerated cognitive decline in people with Alzheimer pathology. Mol. Psychiatry *25*, 2932–2941. https://doi.org/10.1038/s41380-019-0375-7.

Ayton, S., Portbury, S., Kalinowski, P., Agarwal, P., Diouf, I., Schneider, J.A., Morris, M.C., and Bush, A.I. (2021). Regional brain iron associated with deterioration in Alzheimer's disease: a large cohort study and theoretical significance. Alzheimer's Dement. *17*, 1244–1256. https://doi.org/10.1002/alz.12282.

Bagnato, F., Hametner, S., Yao, B., Van Gelderen, P., Merkle, H., Cantor, F.K., Lassmann, H., and Duyn, J.H. (2011). Tracking iron in multiple sclerosis: a combined imaging and histopathological study at 7 Tesla. In Brain, pp. 3599–3612.

Bulk, M., Hegeman-Kleinn, I., Kenkhuis, B., Suidgeest, E., van Roon-Mom, W., Lewerenz, J., van Duinen, S., Ronen, I., and van der Weerd, L. (2020). Pathological characterization of T2*-weighted MRI contrast in the striatum of Huntington's disease patients. NeuroImage Clin. *28*, 102498. https://doi.org/10.1016/j.nicl.2020.102498.

Burm, S.M., Zuiderwijk-Sick, E.A., T Jong, A.E.J., Van Der Putten, C., Veth, J., Kondova, I., and Bajramovic, J.J. (2015). Inflammasome-induced IL-1β secretion in microglia is characterized by delayed kinetics and is only partially dependent on inflammatory caspases. J. Neurosci. *35*, 678–687. https://doi.org/10.1523/jneurosci.2510-14.2015.

Butovsky, O., Jedrychowski, M.P., Moore, C.S., Cialic, R., Lanser, A.J., Gabriely, G., Koeglsperger, T., Dake, B., Wu, P.M., Doykan, C.E., et al. (2014). Identification of a unique TGF-β-dependent molecular and functional signature in microglia. Nat. Neurosci. *17*, 131–143. https://doi.org/10.1038/nn.3599.

Damulina, A., Pirpamer, L., Soellradl, M., Sackl, M., Tinauer, C., Hofer, E., Enzinger, C., Gesierich, B., Duering, M., Ropele, S., et al. (2020). Cross-sectional and longitudinal assessment of brain iron level in Alzheimer disease using 3-T MRI. Radiology *296*, 619–626. https://doi.org/10.1148/radiol.2020192541.

Devos, D., Moreau, C., Kyheng, M., Garçon, G., Rolland, A.S., Blasco, H., Gelé, P., Timothée Lenglet, T., Veyrat-Durebex, C., Corcia, P., et al. (2019). A ferroptosis–based panel of prognostic biomarkers for Amyotrophic Lateral Sclerosis. Sci. Rep. *9*, 2918. https://doi.org/10.1038/s41598-019-39739-5.

Divakaruni, A.S., Paradyse, A., Ferrick, D.A., Murphy, A.N., and Jastroch, M. (2014). Analysis and interpretation of microplate-based oxygen consumption and pH data. In Methods in Enzymology (Academic Press Inc.), pp. 309–354.

Dixon, S.J., Lemberg, K.M., Lamprecht, M.R., Skouta, R., Zaitsev, E.M., Gleason, C.E., Patel, D.N., Bauer, A.J., Cantley, A.M., Yang, W.S., et al. (2012). Ferroptosis: an iron-dependent form of nonapoptotic cell death. Cell *149*, 1060–1072. https://doi.org/10.1016/j.cell.2012.03.042.

Efthymiou, A.G., and Goate, A.M. (2017). Late onset Alzheimer's disease genetics implicates microglial pathways in disease risk. Mol. Neurodegener. *12*, 43. https://doi.org/10.1186/s13024-017-0184-x.

Gan, Z.S., Wang, Q.Q., Li, J.H., Wang, X.L., Wang, Y.Z., and Du, H.H. (2017). Iron reduces M1 macrophage polarization in RAW264.7 macrophages associated with inhibition of STAT1. Mediators Inflamm., 1–9. https://doi.org/10.1155/2017/8570818.

Haenseler, W., Sansom, S.N., Buchrieser, J., Newey, S.E., Moore, C.S., Nicholls, F.J., Chintawar, S., Schnell, C., Antel, J.P., Allen, N.D., et al. (2017). A highly efficient human pluripotent stem cell microglia model displays a neuronal-Co-culture-Specific expression profile and inflammatory response. Stem Cell Rep. *8*, 1727–1742. https://doi.org/10.1016/j.stemcr.2017.05.017.

Holland, R., McIntosh, A.L., Finucane, O.M., Mela, V., Rubio-Araiz, A., Timmons, G., McCarthy, S.A., Gun'ko, Y.K., and Lynch, M.A. (2018). Inflammatory microglia are glycolytic and iron retentive and typify the microglia in APP/PS1 mice. Brain Behav. Immun. *68*, 183–196. https://doi.org/10.1016/j.bbi.2017.10.017.

Jellinger, K., Paulus, W., Grundke-Iqbal, I., Riederer, P., and Youdim, M.B.H. (1990). Brain iron and ferritin in Parkinson's and Alzheimer's diseases. J. Neural Transm. - Park. Dis. Dement. Sect. *2*, 327–340. https://doi.org/10.1007/bf02252926.

Kapralov, A.A., Yang, Q., Dar, H.H., Tyurina, Y.Y., Anthonymuthu, T.S., Kim, R., St. Croix, C.M., Mikulska-Ruminska, K., Liu, B., Shrivastava, I.H., et al. (2020). Redox lipid reprogramming commands susceptibility of macrophages and microglia to ferroptotic death. Nat. Chem. Biol. *16*, 278–290. https://doi.org/10.1038/s41589-019-0462-8.

Kenkhuis, B., Somarakis, A., de Haan, L., Dzyubachyk, O., IJsselsteijn, M.E., de Miranda, N.F.C.C., Lelieveldt, B.P.F., Dijkstra, J., van Roon-Mom, W.M.C., Höllt, T., and van der Weerd, L. (2021). Iron loading is a prominent feature of activated microglia in Alzheimer's disease patients. Acta Neuropathol. Commun. *9*, 27. https://doi.org/10.1186/s40478-021-01126-5.

Keren-Shaul, H., Spinrad, A., Weiner, A., Matcovitch-Natan, O., Dvir-Szternfeld, R., Ulland, T.K., David, E., Baruch, K., Lara-Astaiso, D., Toth, B., et al. (2017). A unique microglia type Associated with restricting development of Alzheimer's disease. Cell *169*, 1276–1290.e17. https://doi.org/10.1016/j.cell.2017.05.018.

Konttinen, H., Cabral-da-Silva, M.e.C., Ohtonen, S., Wojciechowski, S., Shakirzyanova, A., Caligola, S., Giugno, R., Ishchenko, Y., Hernández, D., Fazaludeen, M.F., et al. (2019). PSEN1ΔE9, APPswe, and APOE4 confer disparate phenotypes in human iPSC-derived microglia. Stem Cell Rep. *13*, 669–683. https://doi.org/10.1016/j.stemcr.2019.08.004.

Krasemann, S., Madore, C., Cialic, R., Baufeld, C., Calcagno, N., El Fatimy, R., Beckers, L., O'Loughlin, E., Xu, Y., Fanek, Z., et al. (2017). The TREM2-APOE pathway drives the transcriptional phenotype of dysfunctional microglia in neurodegenerative diseases. Immunity *47*, 566–581.e9. https://doi.org/10.1016/j.immuni.2017.08.008.

Ladiwala, A.R.A., Litt, J., Kane, R.S., Aucoin, D.S., Smith, S.O., Ranjan, S., Davis, J., Van Nostrand, W.E., and Tessier, P.M. (2012). Conformational differences between two amyloid βoligomers of similar size and dissimilar toxicity. J. Biol. Chem. *287*, 24765–24773. https://doi.org/10.1074/jbc.m111.329763.

Lin, Y.T., Seo, J., Gao, F., Feldman, H.M., Wen, H.L., Penney, J., Cam, H.P., Gjoneska, E., Raja, W.K., Cheng, J., et al. (2018). APOE4 causes widespread molecular and cellular alterations associated with Alzheimer's disease phenotypes in human iPSC-derived brain cell types. Neuron *98*, 1141–1154.e7. https://doi.org/10.1016/j.neuron.2018.05.008.

Lopes, K.O., Sparks, D.L., and Streit, W.J. (2008). Microglial dystrophy in the aged and Alzheimer's disease brain is associated with ferritin immunoreactivity. Glia *56*, 1048–1060. https://doi.org/10.1002/glia.20678.

Mathys, H., Davila-Velderrain, J., Peng, Z., Gao, F., Mohammadi, S., Young, J.Z., Menon, M., He, L., Abdurrob, F., Jiang, X., et al. (2019). Single-cell transcriptomic analysis of Alzheimer's disease. Nature *570*, 332–337. https://doi.org/10.1038/s41586-019-1195-2.

McCarthy, R.C., Lu, D.Y., Alkhateeb, A., Gardeck, A.M., Lee, C.H., and Wessling-Resnick, M. (2016). Characterization of a novel adult murine immortalized microglial cell line and its activation by amyloid-beta. J. Neuroinflammation *13*, 21. https://doi.org/10.1186/s12974-016-0484-z.

McCarthy, R.C., Sosa, J.C., Gardeck, A.M., Baez, A.S., Lee, C.H., and Wessling-Resnick, M. (2018). Inflammation-induced iron transport and metabolism by brain microglia. J. Biol. Chem. *293*, 7853–7863. https://doi.org/10.1074/jbc.ra118.001949.

McIntosh, A., Mela, V., Harty, C., Minogue, A.M., Costello, D.A., Kerskens, C., and Lynch, M.A. (2019). Iron accumulation in microglia triggers a cascade of events that leads to altered metabolism and compromised function in APP/PS1 mice. Brain Pathol. *29*, 606–621. https://doi.org/10.1111/bpa.12704.

Nakamura, K., Kawakami, T., Yamamoto, N., Tomizawa, M., Fujiwara, T., Ishii, T., Harigae, H., and Ogasawara, K. (2016). Activation of the NLRP3 inflammasome by cellular labile iron. Exp. Hematol. *44*, 116–124. https://doi.org/10.1016/j.exphem.2015.11.002.

Nnah, I.C., Lee, C.H., and Wessling-Resnick, M. (2020). Iron potentiates microglial interleukin-1β secretion induced by amyloid-β. J. Neurochem. *154*, 177–189. https://doi.org/10.1111/jnc.14906.

Paolicelli, R.C., Bolasco, G., Pagani, F., Maggi, L., Scianni, M., Panzanelli, P., Giustetto, M., Ferreira, T.A., Guiducci, E., Dumas, L., et al. (2011). Synaptic pruning by microglia is necessary for normal brain development. Science *333*, 1456–1458. https://doi.org/10.1126/science.1202529.

van der Poel, M., Ulas, T., Mizee, M.R., Hsiao, C.-C., Miedema, S.S.M., Adelia Schuurman, K.G., Helder, B., Tas, S.W., Schultze, J.L., Hamann, J., and Huitinga, I. (2019). Transcriptional profiling of human microglia reveals grey–white matter heterogeneity and multiple sclerosis-associated changes. Nat. Commun. *10*, 1139. https://doi.org/10.1038/s41467-019-08976-7.

Popescu, B.F., Frischer, J.M., Webb, S.M., Tham, M., Adiele, R.C., Robinson, C.A., Fitz-Gibbon, P.D., Weigand, S.D., Metz, I., Nehzati, S., et al. (2017). Pathogenic implications of distinct patterns of iron and zinc in chronic MS lesions. Acta Neuropathol. *134*, 45–64. https://doi.org/10.1007/s00401-017-1696-8.

Prinz, M., Jung, S., and Priller, J. (2019). Microglia biology: one century of evolving concepts. Cell *179*, 292–311. https://doi.org/10.1016/j.cell.2019.08.053.

Rouault, T.A. (2013). Iron metabolism in the CNS: implications for neurodegenerative diseases. Nat. Rev. Neurosci. *14*, 551–564. https://doi.org/10.1038/nrn3453.

Salter, M.W., and Stevens, B. (2017). Microglia emerge as central players in brain disease. Nat. Med. *23*, 1018–1027. https://doi.org/10.1038/nm.4397.

Simmons, D.A., Casale, M., Alcon, B., Pham, N., Narayan, N., and Lynch, G. (2007). Ferritin accumulation in dystrophic microglia is an early event in the development of Huntington's disease. Glia *55*, 1074–1084. https://doi.org/10.1002/glia.20526.

Smith, A.M., and Dragunow, M. (2014). The human side of microglia. Trends Neurosci. *37*, 125–135. https://doi.org/10.1016/j.tins.2013.12.001.

Smith, M.A., Harris, P.L.R., Sayre, L.M., and Perry, G. (1997). Iron accumulation in Alzheimer disease is a source of redox-generated free radicals. Proc. Natl. Acad. Sci. U S A *94*, 9866–9868. https://doi.org/10.1073/pnas.94.18.9866.

Thomas, G.E.C., Leyland, L.A., Schrag, A.E., Lees, A.J., Acosta-Cabronero, J., and Weil, R.S. (2020). Brain iron deposition is linked with cognitive severity in Parkinson's disease. J. Neurol. Neurosurg. Psychiatr. *91*, 418–425. https://doi.org/10.1136/jnnp-2019-322042.

Trudler, D., Nazor, K.L., Eisele, Y.S., Grabauskas, T., Dolatabadi, N., Parker, J., Sultan, A., Zhong, Z., Goodwin, M.S., Levites, Y., et al. (2021). Soluble α-synuclein-antibody complexes activate the NLRP3 inflammasome in hiPSC-derived microglia. Proc. Natl. Acad. Sci. U S A *118*. e2025847118. https://doi.org/10.1073/pnas.2025847118.

Wang, J.-Y., Zhuang, Q.-Q., Zhu, L.-B., Zhu, H., Li, T., Li, R., Chen, S.-F., Huang, C.-P., Zhang, X., and Zhu, J.-H. (2016). Meta-analysis of brain iron levels of Parkinson's disease patients determined by postmortem and MRI measurements. Sci. Rep. *6*, 36669. https://doi.org/10.1038/srep36669.

Ward, R.J., Zucca, F.A., Duyn, J.H., Crichton, R.R., and Zecca, L. (2014). The role of iron in brain ageing and neurodegenerative disorders. Lancet Neurol. *13*, 1045–1060. https://doi.org/10.1016/s1474-4422(14)70117-6.

Yauger, Y.J., Bermudez, S., Moritz, K.E., Glaser, E., Stoica, B., and Byrnes, K.R. (2019). Iron accentuated reactive oxygen species release by NADPH oxidase in activated microglia contributes to oxidative stress in vitro. J. Neuroinflammation *16*, 41. https://doi.org/10.1186/s12974-019-1430-7.

Stem Cell Reports, Volume *17*

Supplemental Information

Iron accumulation induces oxidative stress, while depressing inflammatory polarization in human iPSC-derived microglia

Boyd Kenkhuis, Michelle van Eekeren, David A. Parfitt, Yavuz Ariyurek, Poulomi Banerjee, Josef Priller, Louise van der Weerd, and Willeke M.C. van Roon-Mom

Supplementary Information

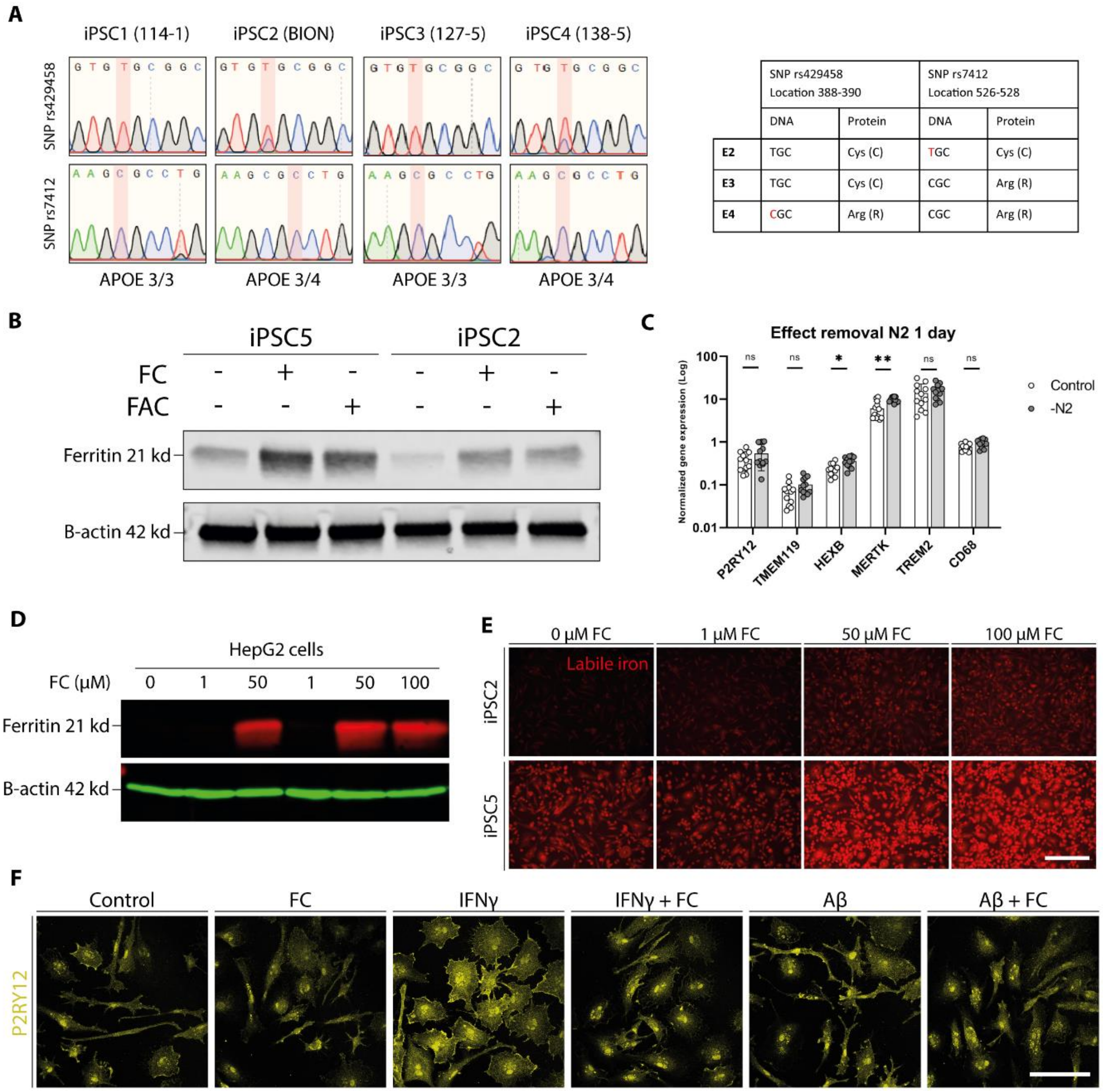

Fig. S1 iPSC and iron loading validation, related to Figure 1 and 2. A *APOE* genotyping of iPSC-lines **B** Western blot for ferritin following ferric citrate (FC) and ferric ammonium citrate (FAC) treatment **C** qPCR analysis of expression of core microglia genes prior to and after 24h N2 removal **D** Western blot for ferritin following FC treatment at different doses with HepG2 cells **E** Representative images of live cell imaging of labile iron showing increased cytosolic iron after 50 and 100 µM, but not after 0 or 1 µM. Scale bar 200 µm **F** P2RY12 expression is increased following IFNγ treatment on immunofluorescence. Scale bar 100 µm

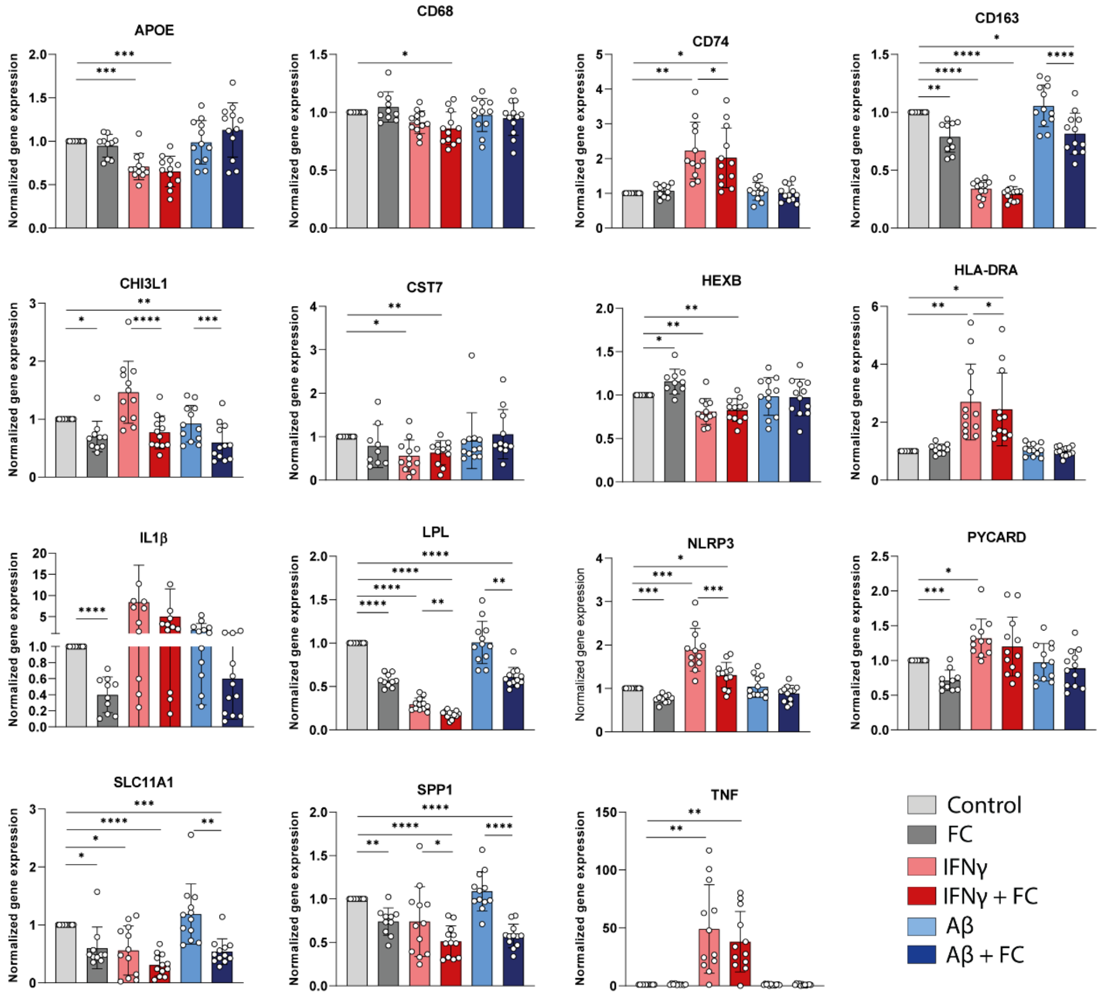

Fig. S2 Bargraphs of targeted gene-expression analysis using the Dynamic Array (Fluidigm), related to Figure 4.

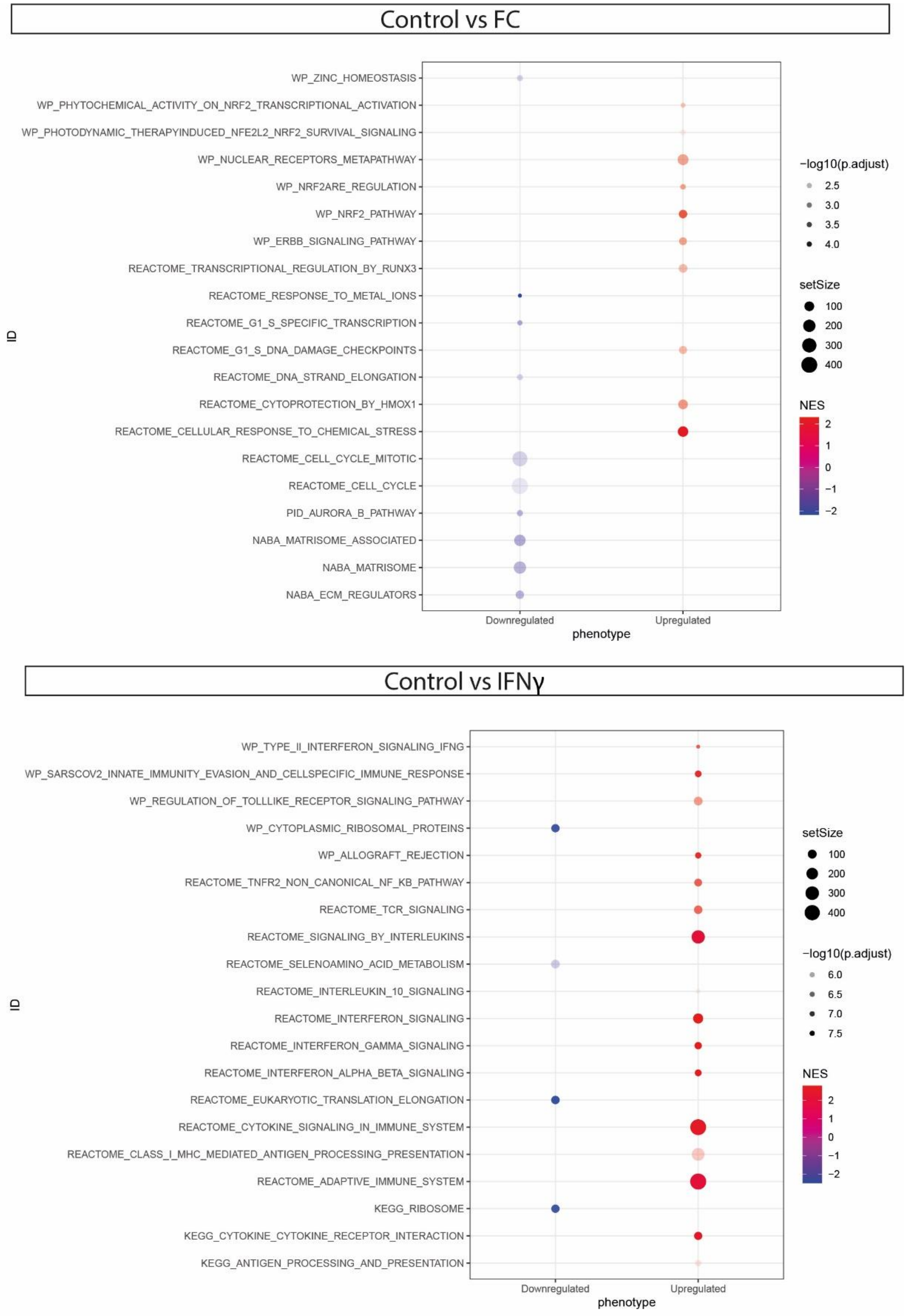

Fig. S3 GSEA analysis results. Legend on next page.

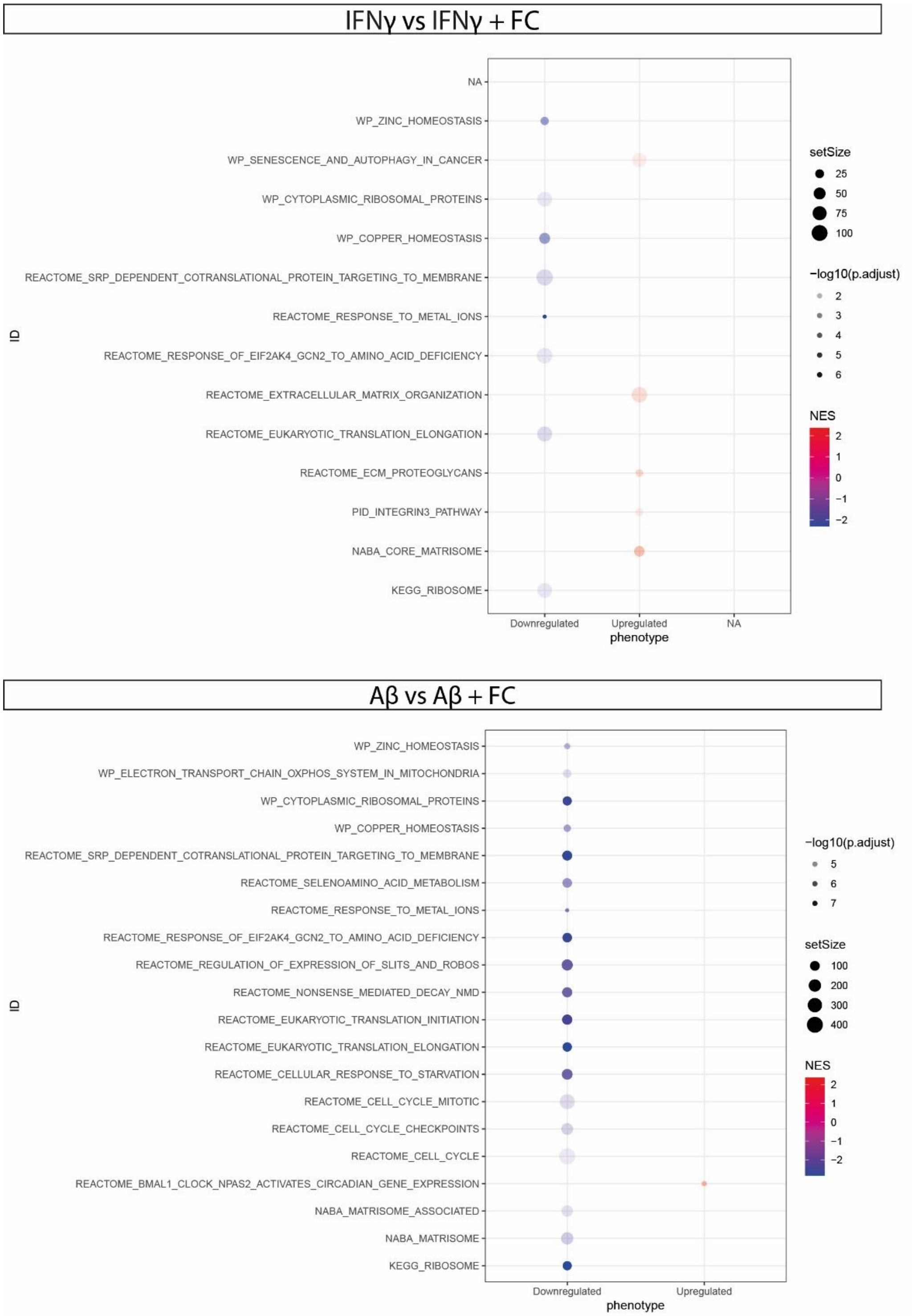

Fig. S3 GSEA analysis results, related to Figure 5. GSEA results from control vs FC and control vs IFNγ show excellent overlap with identified GO terms as was highlighted in the paper. However, although the differentially expressed genes showed considerable overlap between control vs FC and Aβ vs Aβ + FC, considering Aβ had little effect, we do see differences in identified GSEA pathways. These differences are primarily in the omission of the upregulated pathways concerning oxidative stress. The identified downregulated pathways in metal ion metabolism do overlap and appear to be more robust.

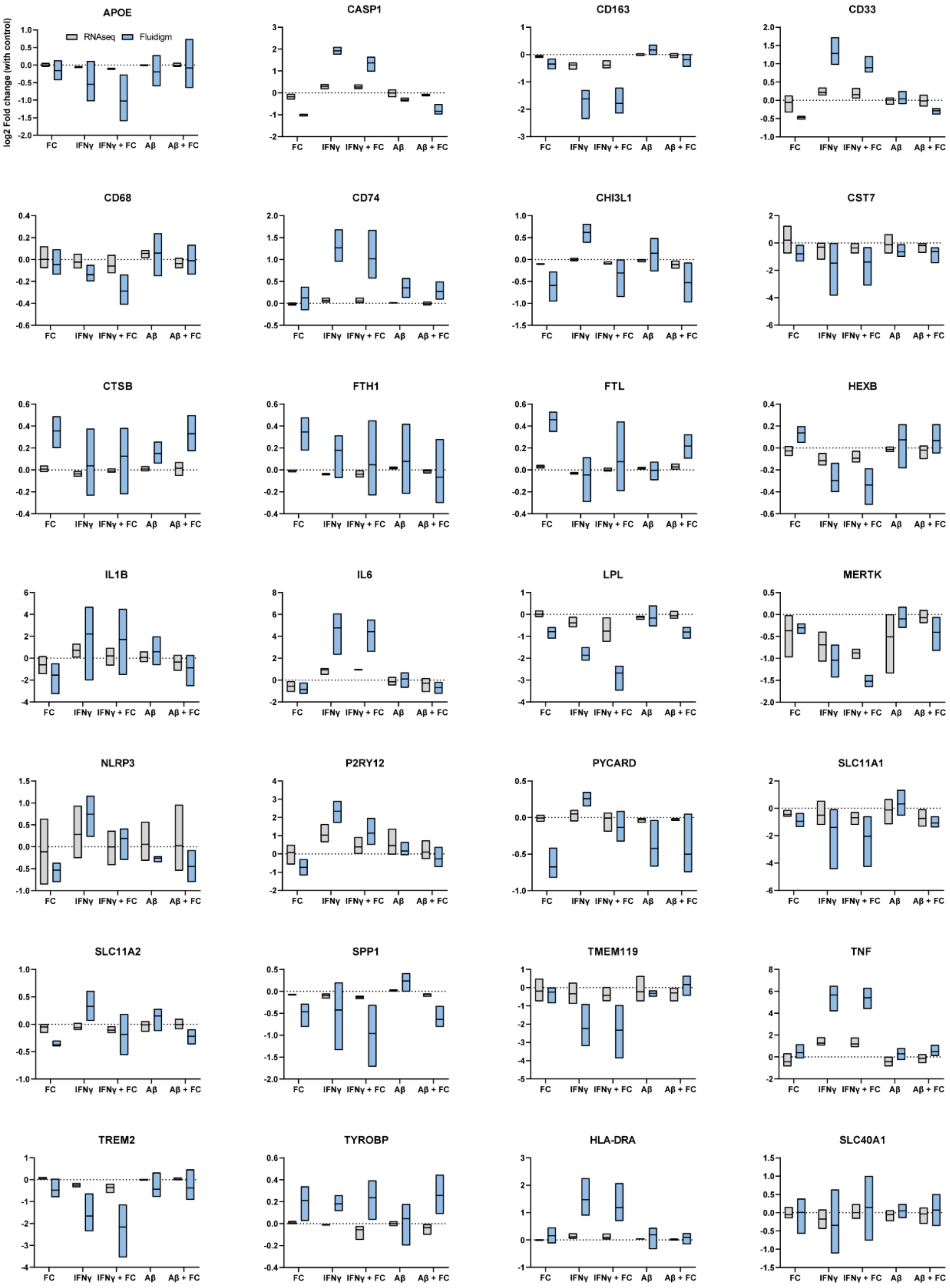

Fig. S4 Comparison log2 fold changes RNAseq (grey) vs fluidigm targeted gene expression analysis (blue), related to Figure 4 and 5

Table S1. Cell line characteristics, related to Figure 1

Alias	Age, y	Sex	Clinical Details	ApoE status	Reprogramming method	Cell line id	Reference
iPSC1	49	F	control	ApoE 3/3	Non-integrating Sendai virus	114-1	Buijsen et al. 2018 https://doi.org/10.1016/j.scr.2018.03.018
iPSC2	15-19	M	control	ApoE 3/4	Non-integrating Episomal	BIONi010-C	Commercial: Ebisc https://cells.ebisc.org/BIONi010-C/
iPSC3	44	F	control	ApoE 3/3	Non-integrating Sendai virus	127-5	In house characterization
iPSC4	45	M	control	ApoE 3/4	Non-integrating Sendai virus	138-5	In house characterization

Table S2. Resources, related to experimental procedures

Product	Source	Identifier
**iPSC culturing**		
Matrigel	Corning	#734-1440
mTESR	STEMCELL Technologies	#85870
37 µm Reversible Strainer, large	STEMCELL Technologies	#27250
Trypan Blue Solution, 0.4%	Invitrogen	#15250-061
AggreWell800	STEMCELL Technologies	#34815
AggreWell Rinsing Solution	STEMCELL Technologies	#07010
Accutase	STEMCELL Technologies	#07920
ReLesR	STEMCELL Technologies	#05872
Glutamax	ThemoFisher	#35050-038
Pen/Strep (100x)	Invitrogen	#15140122
2-mercaptoethanol	ThemoFisher	#31350-010
N2-supplement	ThemoFisher	#17502-048
X-VIVO 15	Lonza	BE02-060F
Advanced DMEM/F12	ThemoFisher	#12634-010
Poly-D-lysine hydrobromide	SIGMA-ALDRICH	#P0899-50MG
2% Gelatin solution	SIGMA-ALDRICH	G1393-100mL
**Growth factors**		
Recombinant Human VEGF	Peprotech	100-20-B
Human SCF Recombinant Protein	ThermoFisher	PHC2115
Human BMP4 Recombinant Protein	ThermoFisher	PHC9534
Recombinant Human M-CSF	Peprotech	#300-25
Recombinant Human IL-3	Peprotech	#200-03
Recombinant human IL-34	Peprotech	#200-34
Recombinant human GM-CSF	Peprotech	#300-03
**Treatment substances**		
Human IFN-γ - 100ug	Peprotech	300-02-B
Ferric Ammonium Citrate	Sigma-Aldrich	RES20400-A702X
Ferric Citrate (FC)	Sigma-Aldrich	F3388-250G
Ascorbate	Sigma-Aldrich	PHR1279-1g
Amyloid β-Protein (1-42) (HFIP-treated)	Bachem	4090148.01
Amyloid β-Protein (42-1) (HFIP-treated)	Bachem	4107743.01
Cytochalasin D - 1 mg	ThermoFisher	PHZ1063
Lipopolysacharide (LPS) (From E. Coli O111:B4)	Sigma-Aldrich	L4391-1MG

**Antibodies**		
MAP2 (Rabbit)	ThermoFisher	PA17646
Iba1 (Goat)	Abcam	ab5076
TMEM119 (Rabbit)	Abcam	ab185333
P2RY12 (Rabbit)	Sigma-Aldrich	HPA014518
Ferritin (rabbit)	Abcam	ab75973
Actin (beta) (AC-15)	Novus biologicals	NB600-501
Donkey anti-Goat IgG (H+L) Highly Cross-Adsorbed Secondary Antibody, Alexa Fluor Plus 488	ThermoFisher	A32814
Donkey anti-Rabbit IgG (H+L) Highly Cross-Adsorbed Secondary Antibody, Alexa Fluor Plus 594	ThermoFisher	A32754
goat anti-Rabbit IRDye 800CW	LI-COR	926-32211
goat anti-Mouse IRDye 680CW	LI-COR	926-68070
**IHC/Western Blot/Live cell assays**		
DAPI (1mg)	Sigma-Aldrich	D9542
prolong diamond	ThermoFisher	P36961
cOmplete™ ULTRA Tablets, Mini, EASYpack Protease Inhibitor Cocktail	Roche	5892970001
Pierce™ BCA Protein Assay Kit	ThermoFisher	23225
4–20% Mini-PROTEAN® TGX™ Precast Protein Gels	BIO-RAD	456-1094
Trans-Blot Turbo RTA Mini PVDF Transfer Kit, for 40 blots	BIO-RAD	1704272
FerroOrange	Goryo Chemical	GC904-01
pHrodo Green Zymosan Bioparticles	ThermoFisher	P35365
Opti-MEM I Reduced Serum Medium - 100 ml	Gibco	31985-062
CELL CULTURE MICROPLATE, 96 WELL, PS, F-BOTTOM (CHIMNEY WELL), µCLEAR	Greiner Bio-One	655090
XFe96 FluxPak	Seahorse Biosciences	102416-100
XF Base medium	Seahorse Biosciences	102353-100
D-Glucose 45%	Sigma-Aldrich	G8769
Rotenone	Sigma-Aldrich	D8375
Antimycin A	Sigma-Aldrich	A8674-25MG
FCCP	Sigma-Aldrich	C2920-10 MG
Oligomycin A	Cayman Chemical	11342-5MG
**DNA/RNA/PCR kits**		
ReliaPrep™ RNA Miniprep Systems	Promega	Z6012
ReliaPrep™ gDNA Tissue Miniprep System	Promega	A2052
Transcriptor First Strand cDNA synthesis Kit	Roche	Cat. No. 04 897 030 001
NucleoSpin PCR clean-up kit	Macherey-Nagel	740609.25
KAPA HiFi HotStart ReadyMix	Roche	7958927001
Qubit dsDNA HS Assay Kit	ThermoFisher	Q32854
KAPA HyperPlus Kit	Roche	07962401001

**Software**		
Graphpad Prism (Version 8.00)	Graphpad	https://www.graphpad.com/
LAS X	Leica	www.leica-microsystems.com
Image studio lite (Version 5.2)	LI-COR	https://www.licor.com/bio/image-studio-lite/
Fiji (is ImageJ)	NIH	https://fiji.sc/
Adobe Illustrator 2021	Adobe	https://www.adobe.com/products/illustrator
R (Version 4.0.3)		https://www.R-project.org/.
Rstudio (Version 1.4.1717)		http://www.rstudio.com/
Nf-core/rnaseq (Version 3.2)	Ewels et al. 2019 (Ewels et al., 2019)	https://nf-co.re/rnaseq/3.2/usage
edgeR (Version 3.32.1)	Robinson et al. 2010 (Robinson et al., 2010)	https://bioconductor.org/packages/release/bioc/html/edgeR.html
Gplots (Version 3.1.1)	Warnes et al. 2020	https://CRAN.R-project.org/package=gplots
GSEABase (Version 1.52.1)	Morgan et al. 2021	https://bioconductor.org/packages/release/bioc/html/GSEABase.html
clusterProfiler (Version 3.18.1)	Yu et al. (2021) (Yu et al., 2012)	https://bioconductor.org/packages/release/bioc/html/clusterProfiler.html
gprofiler2 (Version 0.2.0)	Kolberg and Raudvere	https://cran.r-project.org/web/packages/gprofiler2/index.html
matrixStats (Version 0.58.0)	Bengtsson et al. 2021	https://CRAN.R-project.org/package=matrixStats
Tidyverse (Version 1.3.1)	Wickham et al. 2019 (Wickham et al., 2019)	https://tidyverse.tidyverse.org/
Biobase (Version 2.50.0)	Huber et al. 2015 (Huber et al., 2015)	https://bioconductor.org/packages/release/bioc/html/Biobase.html
**Other**		
ND-1000 Spectrophotometer	NanoDrop	
TCS SP8 confocal microscope	Leica	
CellInsight CX7 High-Content Screening platform	ThermoFisher	
BZ-X800 All-in-one fluorescence microscope	Keyence	
IncuCyte live cell imaging platform	Essen Bioscience	
Odyssey Clx	LI-COR	
Trans-Blot Turbo Transfer system	BIO-RAD	
Bioruptor Pico	Diagenode	
Biomark HD	Fluidigm	
Access Array	Fluidigm	
Seahorse XFe96 Analyzer	Agilent	
Qubit 2.0 Fluoremeter	ThermoFisher	
Agilent 2100 Bioanalyzer (Lab-on-a-Chip)	Agilent	
NovaSeq 6000	Illumina	

Table S3. Primersets, related to figure 2, 3 and 4

human_Gene_Exon_Fw	Sequence	Reverse	Sequence
hACTB_F4	AGCAAGCAGGAGTATGACGA	hACTB_R4	AGAAAGGGTGTAACGCAACTAA
hHMBS F1	GCAACGGCGGAAGAAAA	hHMBS R1	CGAGGCTTTCAATGTTGCC
hHPRT1 F4	ACACTGGCAAAACAATGCA	hHPRT1 R4	GGTCCTTTTCACCAGCAAG
hTMEM119_Qex2_Fw	CTGCTGATGTTCATCGTCTGT	hTMEM119_Qex2_Rev	TCACTCTGGTCCACGTACT
hP2RY12_Qex2_Fw	GGATACATTCAAACCCTCCAG	hP2RY12_Qex3_Rev	GAGGACCTGGGTGATTTTGTAG
hMERTK_Qex15_Fw	GGGACCTGCATACTTACTTACTT	hMERTK_Qex16_Rev	TCCATTCCCAGGGCAATATC
hTREM2_Qex1_Fw	CGGCTGCTCATCTTACTCTTT	hTREM2_Qex2_Rev	CAGTGCTTCATGGAGTCATAGG
hFTL_Qex1_Fw2	CAGCCTGGTCAATTTGTACCT	hFTL_Qex2_Rev2	GCCAATTCGCGGAAGAAGTG
hFTH1_Qex2_Fw	ATGAAGCTGCAGAACCAACG	hFTH1_Qex3_Rev	TGCACACTCCATTGCATTCAG
hSLC40A1_Qex7_Fw	TCTGTCAGTCTGCTGTTTGC	hSLC40A1_Qex8_Rev	TCTTGCAGCAACTGTGTCAC
hSLC11A2_Qex4_Fw	ATTTGCAGTCTGGAGCAGTG	hSLC11A2_Qex5_Rev	ACTGACGGTGACATACTTCAGC
hIL1B_Qex5_Fw_Edi	CATGGGATAACGAGGCTTATGT	hIL1B_Qex5_Rev_Edi	CATATGGACCAGACATCACCAA
hNLRP3_Qex4_Fw	TCATGCTGCCTGTTCTCATG	hNLRP3_Qex5_Rev	GCTGGTGCTCAGAACTGAAAAG
hCHI3L1_Qex6_Fw	ACAGCAGCTATGACATTGCC	hCHI3L1_Qex7_Rev	ATCTGTCAGGACTTGCATCCTC
hCD163_Qex6_Fw	ACTGCAAGAACTGGCAATGG	hCD163_Qex7_Rev	TTCAACACGTCCAGAACAGG
hTNF_Qex2_Fw_Edi	CCAGGGACCTCTCTCTAATCA	hTNF_Qex3/4_Rev_Edi	TCAGCTTGAGGGTTTGCTAC
hIL6_Qex3_Fw_Edi	CACTCACCTCTTCAGAACGAAT	hIL6_Qex4_Rev_Edi	GCTGCTTTCACACATGTTACTC
hRPL13a_Qex2_Fw	GCCATCGTGGCTAAACAGGTA	hRPL13a_Qex4_Rev	GTTGGTGTTCATCCGCTTGC
hHEXB_Qex2_Fw	GTCAGAGTGTGATGCTTTCCC	hHEXB_Qex3_Rev	TAAACCTCGTAATGCTCCCCA
hCD68_Qex4_Fw	TTGTCTACCTGAGCTACATGGC	hCD68_Qex5_Rev	TTGCTGCAACTGAAGCTCTG
hPYCARD_Qex3_Fw	TGGATGCTCTGTACGGGAAG	hPYCARD_Qex3_Rev	CCAGGCTGGTGTGAAACTGAA
hCASP1_Qex2_Fw	CACAGGCATGCCAAATTTGC	hCASP1_Qex3/4_Rev	GAGGAGCTGGAAAGGAAGAAAG
hAPOE_Qex3_Fw2	GGTCGCTTTTGGGATTACCTG	hAPOE_Qex4_Rev2	TCAACTCCTTCATGGTCTCGTC
hCD74_Qex4_Fw	TGACAGAGGACCATGTGATGC	hCD74_Qex5/6_Rev	ATCCAGCTCTCAAAGACCTTCC
hCTSB_Qex7_Fw	TACAGCCCGACCTACAAACAG	hCTSB_Qex8_Rev	AGCAGGAAGTCCGAATACACAG
hTYROBP_Qex4_Fw	ACTGAGACCGAGTCGCCTTAT	hTYROBP_Qex5_Rev	ATACGGCCTCTGTGTGTTGAG
hSPP1_Qex5_Fw	TGCCAGCAACCGAAGTTTTC	hSPP1_Qex6_Rev	TGATGTCCTCGTCTGTAGCATC
hSLC11A1_Qex2_Fw	TCCCCATCCCAGACACAAAAC	hSLC11A1_Qex3_Rev	AGCAATGCTCATGAGGAAGC
hHLA-DRA_Qex1_Fw	AGTCCCTGTGCTAGGATTTTTCA	hHLA-DRA_Qex2_Rev	ACATAAACTCGCCTGATTGGTC
hLPL_Qex2_Fw	TCATTCCCGGAGTAGCAGAGT	hLPL_Qex3_Rev	GGCCACAAGTTTTGGCACC
hCST7_Qex2_Fw	TCAACAACTGCACGAACGAC	hCST7_Qex3_Rev	GCAGGTGCTGGTTTTTCTTG
hCD33_Qex2_Fw	AAATCTCCCCAGCTCTCTGTG	hCD33_Qex3_Rev	AGCAGGTCAGGTTTTTGGAG
**Sanger sequencing**			
hAPOE_ex4_Fw5	AACAACTGACCCCGGTGGCG	hAPOE_ex4_Rev5	ATGGCGCTGAGGCCGCGCTC

Table S4. DEG result (separate file), related to Figure 5. All identified differentially expressed genes (DEGs) between treatment groups reported alongside their log foldchange (logFC), log counts per million (logCPM), F-statistic (F), *P*-Value and false discovery rate (FDR)

Table S5. GO analysis results (separate file), related to Figure 5. All identified significantly affected GO terms in the 4 different identified modules, on which Fig. 5F is based.

Table S6. GSEA analysis results (separate file), related to Figure 5. All overrepresented classes of genes identified using gene set enrichment analysis (GSEA), on which Fig. S3 is based.

Experimental procedures

Differentiation of iPSC-MG

IPSC-MG were differentiated following a protocol by Haenseler *et al* (Haenseler et al., 2017), with minor adaptations for upscaling of the production of iPSC-MG as described by Banerjee *et al* (Banerjee et al., 2020). iPSCs were cultured in mTESR (STEMCELL Technologies) until 80% confluent, after which they were lifted using Accutase (STEMCELL Technologies), counted and plated in an Aggrewell800 (STEMCELL Technologies) at a density of approximately 13.000 cells per miniwell to form embryoid bodies (EBs). Cells were cultured in mTESR supplemented with 50ng/mL VEGF (Peprotech), 50 ng/mL BMP4 (ThermoFisher), and 20 ng/mL SCF (ThermoFisher) for 5 days, after which EBs were lifted, collected over a strainer and plated in a 6-well plate at 12 EBs/well in 4mL X-VIVO 15 (Lonza) with 50ng/mL m-CSF (Peprotech) and 25 ng/mL IL-3 (Peprotech). Media was changed weekly and the development of myeloid precursors was tracked by phase contrast microscopy twice a week. After approximately 4 weeks, myeloid precursors were collected from the supernatant weekly, and plated on 0.01 µg/mL Poly-D-lysine (Sigma-Aldrich) and 0.01% gelatin (Sigma-Aldrich) coated plates at a density of 50.000/cm^2. Cells were cultured in microglia media, consisting of advanced DMEM/F12 (ThermoFisher) supplemented with Glutamax (ThermoFisher), N2-supplement (ThermoFisher), 2-mercaptoethanol (ThermoFisher), 100 ng/mL IL-34 (Peprotech), and 10 ng/mL GM-CSF (Peprotech). Half of the media was changed every other day and iPSC-MG were considered mature after 14 days.

Organoid generation and hiPSC-MG integration

Forebrain organoids were generated from control iPSC (iPSC4) using the STEMdiff Cerebral Organoid kit (StemCell Technologies; based on Lancaster et al. (Lancaster and Knoblich, 2014)), according to the manufacturer's instructions. Briefly, iPSCs were collected as a single cell suspension using Accutase before being resuspended at 9000 cells (in 100 µL EB formation media) per well in a V-bottom 96 well plate, to form embryoid bodies (EBs; day 0). Media was topped up with additional 100 µL at day 2 and 4. On day 5, single EBs were transferred to a 48 well plate in induction media. On day 7, EBs were embedded in a drop (25-50 µL) of Matrigel and incubated in expansion media (12 EBs per well of a 6 well plate). At day 10, media was swapped for maturation media and organoids were cultured on an orbital shaker at 65rpm at 37, with media changed twice weekly for long-term culture. At day 43, organoids were transferred to a 24 well plate and $2x10^5$ hiPSC-MGs were collected and added to the well. Integration of hiPSC-MG into the organoids was tracked using brightfield imaging, over the next week.

Sanger sequencing

iPSCs were lifted using Accutase (STEMCELL technologies), spun down and a pellet was frozen for DNA isolation. DNA was isolated using the gDNA tissue miniprep system (Reliaprep) and purity of DNA was assessed using a ND-1000 Spectrophotometer (Nanodrop). PCR was performed using primers covering single-nucleotide polymorphisms rs429458 and rs7412, which determine the *APOE* genotype. Subsequently, the primer product was cleaned using the NucleoSpin PCR clean-up kit (Macherey-Nagel) before Sanger sequencing.

Immunofluorescence

For IF stainings, hiPSC-MGs were washed with PBS 24 hours post-intervention and fixed with 4% PFA for 10 min. Subsequently, cells were washed with PBS before permeabilization for 5 min with 0.1% Triton X-100 in PBS and subsequent washing with PBS. Organoids with integrated hiPSC-MG were washed with PBS, fixed in 4% PFA overnight at 4°C, washed with PBS and cryoprotected for 2-3 days in 30% sucrose at 4°C. Organoids were frozen in ethanol cooled with dry ice and stored at -80°C. Frozen organoids were cut on a cryostat (Leica CM3050) at 16 µm. Non-specific antigens were blocked for 1 hour with blocking solution (3% BSA in PBS). Cells were incubated overnight at 4°C with the following primary antibodies diluted in blocking solution: goat anti-Iba1 (1:500, Abcam, ab5076) with rabbit anti-TMEM119 (1:100, Abcam, ab185333), rabbit anti-P2RY12 (1:200, Sigma-Aldrich, HPA014518) or rabbit anti-MAP2 (1:1000, ThermoFisher, PA17646). Cells were washed in PBS and incubated with the secondary

antibodies donkey-anti-goat Alexa 488 (1:500, ThermoFisher, #A32814) and donkey-anti-rabbit (1:500, ThermoFisher, #A32754) in blocking solution for 3 hours at room temperature. Cells were washed with PBS and incubated for 5 min with 0.1 µg/mL DAPI (Sigma Aldrich) in PBS to label nuclei. Finally, cells on coverslips were mounted with ProLong diamond Antifade Mountant (ThermoFisher), while cells in 96-well plates were covered with PBS and imaged within 1 week. All PBS wash steps were performed three times for five minutes.

Imaging

Coverslips were imaged either using a BZ-X800 All-in-one fluorescence microscope (Keyence) with 10x objective for low magnification whole overview images (1460 x 1095 µm, at 960x720 pixels), or a TCS SP8 confocal microscope (Leica) with 40x objective for high-magnification images (385 x 385 µm, at 2048 x 2048 pixels) . All imaging conditions were identical for all experimental conditions for different stains (e.g. exposure time and laser power). HiPSC-MGs cultured and stained in 96-well flat bottom plates were imaged using the CellInsight CX7 High-Content Screening platform (ThermoFisher). Nuclei were identified using DAPI, and all identified DAPI+ nuclei were assessed for positivity of the cells for Iba1 (threshold determined based on negative control in which primary anti-Iba1 antibody was omitted).

Labile iron imaging

HiPSC-MG were cultured in 96-well flat bottom plates and following 24 h treatment with FC/IFNγ/AB hiPSC-MG were washed twice with PBS and incubated with 200uM FerroOrange (Goryo chemical) in HBSS for 30 min. Subsequently, hiPSC-MG were washed with PBS and Opti-MEM (Gibco) was added for live cell imaging BZ-X800 All-in-one fluorescence microscope (Keyence). Images were obtained with a 10x objective (1460 x 1095 µm, at 960x720 pixels). Labile iron quantity was quantified using Fiji (NIH), by firstly applying automated thresholding for intracellular iron using a iterative self-organzing data analysis (Isodata) algorithm (Ridler and Calvard, 1978), and subsequently measuring mean fluorescent intensity.

Western Blot

HiPSC-MGs cultured in a 12-well plate were washed with and frozen at -20 °C. After 24 hours, radioimmunoprecipitation assay buffer, supplemented with a protease inhibitor cocktail (Roche), was used lyse the cells. Subsequently, cells were sonicated using a Bioruptor Pico water bath sonicator (Diagenode) for 10 min. Protein concentrations were determined using Pierce BCA Protein Assay Kit (Thermo Fisher). 10 µg total protein was separated on a 4-20 % Mini-PROTEAN TGX Precast Protein Gel (BIO-RAD) under reducing conditions, and transferred to polyvinylidene difluoride (PVDF) membranes using a Trans-Blot Turbo Transfer system (BIO-RAD). PVDF membranes were blocked for 1 h with 5% milk in Tris buffered saline with 0.1% Tween 20 (hereafter TBST), and probed with rabbit anti-ferritin (1:1000, Abcam, ab75973) and mouse anti-β-actin (1:5000, Novus biologicals, NB600-501) overnight at 4 °C. Membranes were washed three times with TBST and incubated with goat anti-Rabbit IRDye 800CW (1:10000, LI-COR, 926-32211) and goat anti-Mouse IRDye 680CW (1:1000, LI-COR, 926-68070) and imaged using an Odyssey CLx infrared imaging system (LI-COR). Bands were quantified using Image Studio (Version 5.2, LI-COR), and normalized to β-actin signal.

Gene-expression analysis

RNA from hiPSC-MGs was collected using ReliaPrep RNA Miniprep system (Promega). Briefly, hiPSC-MGs cultured in a 12-well plate were washed with PBS and lysis buffer was added directly to the plate. Cells were pipetted repeatedly to shear the DNA and buffer was collected in an Eppendorf tube after which the protocol was followed as instructed by the manufacturer. Concentration and purity of RNA was assessed using a ND-1000 Spectrophotometer (Nanodrop).

Targeted gene expression analysis

200ng of RNA was converted into cDNA using the Transcriptor First Strand cDNA synthesis Kit (Roche). Gene expression was assessed using a 96x96 Dynamic Array (Fluidigm), which allows for testing of 96 samples and 32 primer sets in a single assay. A total list of primer sets used can be found in table S3. The Dynamic array was performed following manufacturer's guidelines. In brief, cDNA was pre-amplified for 11 cycles using a mixture of all primer sets, following protocol PN 100-5875 (Fluidigm). Subsequently, following protocol PN100-9792 (Fluidigm), a 96x96

dynamic array with integrated fluidic circuit was loaded with all 96 samples on one side and the 32 primersets (each 3 times) on the other side. Using an Access Array machine (Fluidigm), the cDNA samples and primers are mixed so that each well of the 96x96 contains one cDNA samples and 1 primerset (total of 9216 wells). The 96x96 dynamic array was then loaded in a Biomark HD (Fluidigm), which performs a real-time quantitative PCR reaction. Cq values were exported, and were normalized by substracting the Cq values of reference genes *ACTB* and *RPL13a* for highly expressed genes (low Cq values) or of reference genes *HMBS* and *HPRT1* for lowly expressed genes (high Cq values). Cq values were converted into relative expression counts and normalized to control treatment for each biological replicate.

RNAseq and analysis

For the generation of the full-length cDNA of the RNA-seq data, we used the method that was described as mcSCRB-seq (Bagnoli et al., 2018), with the following modifications to the workflow: instead of single cells, we used 100 ng isolated total RNA. Further, the PEG8000 was replaced with Polyvinylpyrrolidone (PVP) to reduce the viscosity of the reaction. We noticed that 0.5% PVP gave similar results as 7.5% PEG8000. The oligodT primer design was also different because it only contains the unique molecular index (UMI) with a PCR handle, which is partially Read 2 sequence primer from the Illumina platform (GAGTTCAGACGTGTGCTCTTCCGATCTNNNYRNNNYRNNNYRNNNTTTTTTTTTTTTTTTTTTTTTTTTTTTTTVN). The samples were uniquely barcoded using the PCR handle in the oligodT primer which completed the Read2 sequence primer, added the i7 Illumina barcode and the p7 sequence (CAAGCAGAAGACGGCATACGAGAT[i7_barcode]GTGACTGGAGTTCAGACGTGTGCTCTTCCGATCT). The i7 Illumina barcode served as the sample barcode. For the PCR reaction (9x cycle) we used the 2x KAPA HiFi HotStart ReadyMix (Roche) to generate the full-length cDNA libraries. After quantification of each sample using a Qubit 2.0 fluoremeter (ThermoFisher), the cDNA libraries were pooled together. The pooled cDNA libraries were subjected to an Illumina library prep. For this step, we used the KAPA HyperPlus kit (Roche) following the instruction of the manufacturer. Briefly, the pool of cDNA samples was fragmented for 5 min using the fragmentation enzyme from the kit followed by an End-Repair and A-tailing step. The fragmented cDNA was subjected to a double SPRI purification round (0.6x followed by 0.8x SPRI cleanup). The custom indexed adapter barcoded the pool of samples using the i5 Illumina barcode and introduced the p5 sequence to complete the Illumina library (top_adapter: AATGATACGGCGACCACCGAGATCTACAC[i5]ACACTCTTTCCCTACACGACGCTCTTCCGATC*T, bot_adapter GATCGGAAGAGCGT/3InvdT/). The ligated products were purified using 0.8x Ampure beads and the PCR with 2x Kapa ReadyMix (Roche) with the p5 and p7 primers was performed (9x cycle) to enrich for the correct molecules. After an additional double SPRI size selection, the Illumina library was quantified using a Qubit 2.0 fluoremeter (ThermoFisher) and checked on an Agilent 2100 Bioanalyzer (Lab-on-a-chip) for size distribution. Illumina paired-end sequencing of the library was done on a NovaSeq 6000 (Illumina) using manufacterer's instructions. After sequencing, Read 1 contained the cDNA information and read 2 only the UMI. The 3 prime RNA-seq data were analyzed using the RNA-seq module of nf-core (Version 3.2). First the data was mapped against the human genome (hg38) using STAR for alignment and Salmon for quantification, adopting default settings. For the pipeline to run effortlessly, the UMI in the read2 fastq file was added to the beginning of the same read id in the read1 fastq file. The modified Read1 fastq file then starts with the UMI and is followed by the cDNA sequence, which was used in the RNA-seq pipeline.

All subsequent analyses were conducted with the R/Bioconductor platform using R (Version 4.0.5). Raw countmatrix was converted in to counts per million (CPM) using the "cpm" function of edgeR (Version 3.32.1) (Robinson et al., 2010) and filtered for lowly expressed genes with (CPM > 10, for at least 4 samples). Hierarchical clustering was performed together with published data for iPSC-derived microglia (Abud et al., 2017; Kontinnen et al) using the hclust function with Euclidean distance on FPKM expression data for a subset of microglia genes published by Kontinnen et al. Filtered CPM counts were normalized using the trimmed mean of M values (TMM) approach using the calcNormFactors function of the edgeR package to normalize for batch effect. TMM uses raw data to estimate the appropriate scaling factors for different samples,

accounting for the different experimental conditions of the samples (Robinson and Oshlack, 2010). As only the final 200 basepairs of the 3’ end were prepared in library prep, genecounts were not normalized for transcript length. Principal component analysis was performed on filtered normalized data with the prcomp function. Following, differential gene expression analysis was performed using the glmQLFTest function from edgeR. Differential expression was assessed for each gene using an exact text analogous to Fisher’s exact test, but adapted for overdispersed data. We adjusted for any baseline differences between the different iPSC lines by fitting an additive model, to make comparison between treatments more precise, as was described in section 3.4.2 of the edgeR vignette (Chen et al.). Heatmaps were produced using the heatmap.2 function of gplots (Version 3.1.1) based on hierarchically clustered data of the top 60 differentially expressed genes (DEGs) for IFNγ treated samples and all DEGs for all other groups. Modules were extracted and functional enrichment analysis was performed using both Gene Ontology (GO) enrichment analysis and Gene Set Enrichment Analysis (GSEA) using the gost function of gprofiler2 (Version 0.2.0) and the GSEA function of the clusterProfiler (Version 3.18.1) package respectively. Results from RNAseq analysis were validated via comparison with results from targeted gene expression analysis using the Dynamic Array (Fluidigm). Although the log2 fold changes differ, the direction of log2 fold changes between different treatment groups is the same in both targeted gene expression analysis with a Dynamic Array and unbiased RNAseq analysis (Fig. S4).

Phagocytosis assay
Phagocytosis assay was performed using pHrodo Zymosan A particles (ThermoFisher) and imaged using an IncuCyte S3 Live-Cell Analysis System (Essen Bioscience). HiPSC-MGs were treated with previously defined conditions for 24 h, and additionally separate wells were treated with 100 ng/mL LPS (Sigma-Aldrich) for 24h or 10 Cytochalasin D (ThermoFisher) for 30 min. Media was replaced with Opti-MEM (Gibco) containing 25 µg/mL pHrodo particles and subsequently live-cell imaged every 30 min with a FITC filter (Exposure: 400 ms) and phase imaging for 24 h. Total fluorescence was quantified using Incucyte S3 software (Essen Bioscience), and normalized to cell confluence, which was quantified on the phase images using Incucyte software. Data was exported and area under the curve (AUC) and half time for max phagocytosis were calculated using Graphpad Prism (Version 8.00, La Jolla, San Diego, CA, USA).

Seahorse bioenergetics assay
Bio-energetic properties of the hiPSC-MGs were assessed using Seahorse XFe96 Analyzer (Agilent) for real-time cell metabolic analysis. HiPSC-MGs were cultured in 96-well Seahorse XF cell culture plate (Agilent) for 14 days as normal, before treatments as described. After treatment, both glycolysis and mitochondrial functional were assessed simultaneously, following a previously described protocol (Van den Bossche et al., 2015). In brief, treatment media was replaced with XF assay medium supplemented with 200 mM Glutamax (ThermoFisher) and cells were incubated in a 0% CO2 incubator for approximately 1h. Electron transport chain modulators were sequentially injected in a Seahorse XF96 analyzer: 25 mM glucose (to induce glycolysis); 1 µM oligomycin (to inhibit ATP synthase); 1 µM carbonyl cyanide-4-phenylhydrazone (FCCP; to uncouple mitochondrial respiration and assess maximal and spare respiratory capacity); 1 µM rotenone combined with 1 µM antimycin A (RA; to block mitochondrial complex I and III, and assess non-mitochondrial oxygen consumption; all Sigma-Aldrich). Both extracellular acidification rate (ECAR; mpH/min) and oxygen consumption rate (OCR; pMoles O_2/min) were assessed in real time, which were used to calculate glycolytic and mitochondrial respiration parameters, according to manufacturer’s instructions. After the assay, cells were stained using 0.1 µg/mL DAPI (Sigma-Aldrich), and number of nuclei were counted using the CellInsight CX7 High-Content Screening platform (ThermoFisher), and used to normalized the results.

Replicates

Optimal reproducibility was attempted by studying all conditions simultaneously three times for each individual iPSC lines. Most assays have an n = 12, meaning 3 independent experiments were performed with 4 iPSC-lines. An experiment was considered independent when it was performed with a batch of iPSC-MG that was differentiated separately from precursor myeloid cells to microglia. A technical replicate was considered as another well from the same differentiation from myeloid cells to microglia. Technical replicates were used for the, labile iron imaging, phagocytosis assay and seahorse metabolism assay, and were averaged to obtain 1 datapoint.

References

Bagnoli, J.W., Ziegenhain, C., Janjic, A., Wange, L.E., Vieth, B., Parekh, S., Geuder, J., Hellmann, I., and Enard, W. (2018). Sensitive and powerful single-cell RNA sequencing using mcSCRB-seq. Nat. Commun. 2018 91 *9*, 1–8.

Banerjee, P., Paza, E., Perkins, E.M., James, O.G., Kenkhuis, B., Lloyd, A.F., Burr, K., Story, D., Yusuf, D., He, X., et al. (2020). Generation of pure monocultures of human microglia-like cells from induced pluripotent stem cells. Stem Cell Res. *49*.

Van den Bossche, J., Baardman, J., and de Winther, M.P.J. (2015). Metabolic characterization of polarized M1 and M2 bone marrow-derived macrophages using real-time extracellular flux analysis. J. Vis. Exp. *2015*, 53424.

Chen, Y., McCarthy, D., Ritchie, M., Robinson, M., Smyth, G., and Hall, E. edgeR: differential analysis of sequence read count data User's Guide.

Ewels, P., Hammarén, R., Peltzer, A., Moreno, D., Garcia, M., rfenouil, marchoeppner, Panneerselvam, S., F., S., jun-wan, et al. (2019). nf-core/rnaseq: nf-core/rnaseq version 1.4.2.

Haenseler, W., Sansom, S.N., Buchrieser, J., Newey, S.E., Moore, C.S., Nicholls, F.J., Chintawar, S., Schnell, C., Antel, J.P., Allen, N.D., et al. (2017). A Highly Efficient Human Pluripotent Stem Cell Microglia Model Displays a Neuronal-Co-culture-Specific Expression Profile and Inflammatory Response. Stem Cell Reports *8*, 1727–1742.

Huber, W., Carey, V.J., Gentleman, R., Anders, S., Carlson, M., Carvalho, B.S., Bravo, H.C., Davis, S., Gatto, L., Girke, T., et al. (2015). Orchestrating high-throughput genomic analysis with Bioconductor. Nat. Methods 2015 122 *12*, 115–121.

Lancaster, M.A., and Knoblich, J.A. (2014). Generation of cerebral organoids from human pluripotent stem cells. Nat. Protoc. 2014 910 *9*, 2329–2340.

Ridler, T.W., and Calvard, S. (1978). PICTURE THRESHOLDING USING AN ITERATIVE SLECTION METHOD. IEEE Trans. Syst. Man Cybern. *SMC-8*, 630–632.

Robinson, M.D., and Oshlack, A. (2010). A scaling normalization method for differential expression analysis of RNA-seq data. Genome Biol. 2010 113 *11*, 1–9.

Robinson, M.D., McCarthy, D.J., and Smyth, G.K. (2010). edgeR: a Bioconductor package for differential expression analysis of digital gene expression data. Bioinformatics *26*, 139–140.

Wickham, H., Averick, M., Bryan, J., Chang, W., McGowan, L.D., François, R., Grolemund, G., Hayes, A., Henry, L., Hester, J., et al. (2019). Welcome to the Tidyverse. J. Open Source Softw. *4*, 1686.

Yu, G., Wang, L.-G., Han, Y., and He, Q.-Y. (2012). clusterProfiler: an R Package for Comparing Biological Themes Among Gene Clusters. Https://Home.Liebertpub.Com/Omi *16*, 284–287.
